# SUMOylation activates ECHS1 for adaptive catabolism in lung cancer

**DOI:** 10.1038/s41419-026-09078-1

**Published:** 2026-07-09

**Authors:** Yalan Chen, Hui Mao, Lili Cai, Qianyu Li, Juan Song, Mingming Zhang, Shanshan Chen, Ziqi Ai, Qiangqiang Xiong, Kexin Liu, Xin Lin, Hongsheng Tan, Tianshi Wang, Qiuju Fan, Yong Zuo, Jianli He, Jinke Cheng, Jun Tu

**Affiliations:** 1 Shanghai Key Laboratory for Tumor Microenvironment and Inflammation, Department of Biochemistry and Molecular Cell Biology, Shanghai Jiao Tong University School of Medicine, Shanghai, China; 2 Core Facility of Basic Medical Sciences, Shanghai Jiao Tong University School of Medicine, Shanghai, China; 3 Cancer Institute, Longhua Hospital, Shanghai University of Traditional Chinese Medicine, Shanghai, China; 4 Experimental Therapeutics Laboratory, Frontiers in Therapeutics Research Center, Ruijin Hospital, Shanghai Jiao Tong University School of Medicine, Shanghai, China; 5 Zhuzhou Hospital Affiliated to Xiangya School of Medicine, Central South University, Zhuzhou, China; 6 Clinical Research Center, Shanghai Jiao Tong University School of Medicine, Shanghai, China; 7 Punan Branch of Renji Hospital, Shanghai Jiao Tong University School of Medicine, Shanghai, China; 8 Hainan Medical University, Hainan Academy of Medical Sciences, Haikou, China

**Keywords:** Cancer metabolism, Sumoylation, Energy metabolism

## Abstract

Cellular metabolism dynamically adapts to nutrient fluctuations, yet the regulatory mechanisms underlying this plasticity remain incompletely understood. In particular, the adaptive activation mechanism of enoyl-CoA hydratase short-chain 1 (ECHS1), a key mitochondrial enzyme for fatty acid and amino acid catabolism, is poorly characterized. Herein, we identify SUMO1 modification at lysine 128 of ECHS1 as an essential activation switch that drives mitochondrial catabolism and restricts growth of lung cancer cells. Nutrients generating crotonyl-CoA (fatty acids, lysine, tryptophan) stimulate this modification, accelerating crotonyl-CoA oxidation to acetyl-CoA. This metabolic adaptation decreases crotonyl-CoA pools, thereby reducing histone crotonylation (e.g., H2BK20cr and H3K27cr). Conversely, SUMOylation deficiency impairs formation of the catalytic ECHS1 homohexamer, increasing crotonyl-CoA accumulation and histone crotonylation. This epigenetically suppresses oxidative phosphorylation and associated ROS generation while activating PI3K-Akt signaling, promoting lung cancer growth in vitro and in vivo. In lung adenocarcinoma patients, H2BK20cr and H3K27cr levels are elevated in tumor tissues and predict poor survival, highlighting their clinical prognostic significance. Collectively, these findings establish ECHS1 SUMOylation as a nutrient-sensitive activation mechanism for adaptive catabolism and reveal mitochondrial SUMOylation as a critical regulator of nuclear epigenetic reprogramming, defining a metabolite-driven paradigm in cancer epigenetics.

**Figure:**
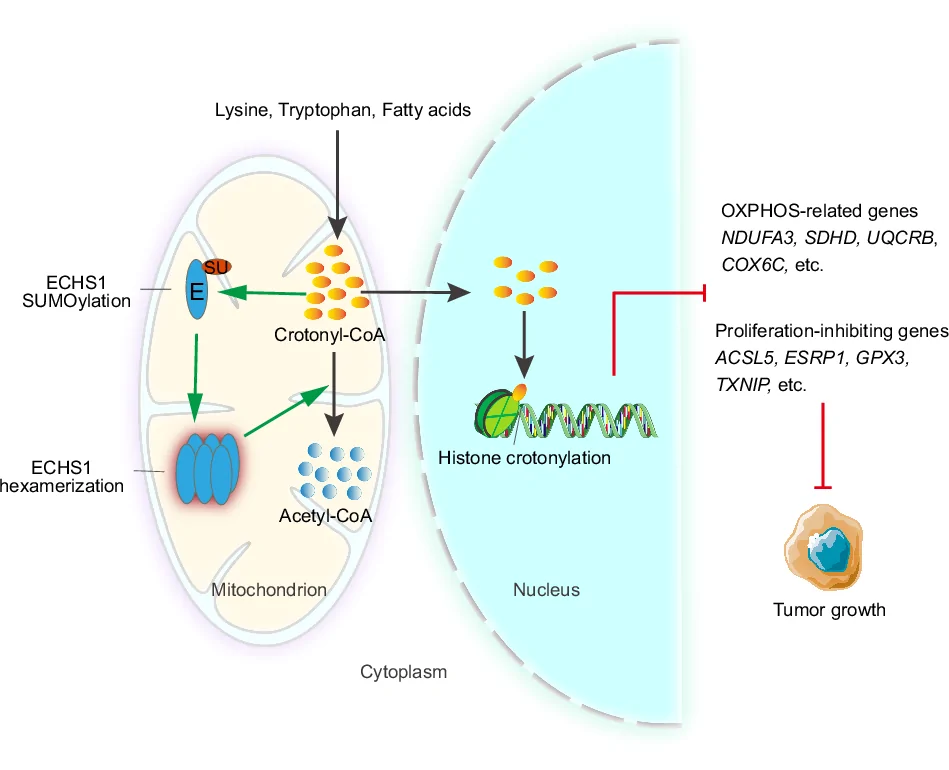

## Introduction

To maintain metabolic homeostasis, cells activate adaptive responses to eliminate nutrient surplus; failure of this process contributes to pathological metabolic reprogramming underlying disorders such as cancer [1, 2]. The oxidative degradation of carbohydrates, fatty acids, and amino acids universally proceeds through a conserved reaction sequence: dehydrogenation-hydration-secondary dehydrogenation. Within this pathway, mitochondrial enoyl-CoA hydratase short-chain 1 (ECHS1) catalyzes the stereospecific hydration of *trans*-Δ^2^-enoyl-CoA to (S)-3-hydroxyacyl-CoA, establishing its fundamental role in oxidizing fatty acids, lysine, tryptophan, and branched-chain amino acids (BCAAs) [3]. ECHS1 translocates to the mitochondrial matrix, where it assembles into a catalytically active homohexamer [4]. It exhibits the highest substrate affinity for crotonyl-CoA, catalyzing its conversion to (S)-3-hydroxybutyryl-CoA, a precursor for acetyl-CoA generation. Both crotonyl-CoA and acetyl-CoA serve as histone modification moieties that exert distinct transcriptional regulations [5]. Given that writers and erasers of crotonylation found to date are largely shared with those for acetylation, intracellular concentrations of these acyl-CoAs probably determine their competitive modification dynamics [6]. While crotonyl-CoA biosynthesis can be mediated by glutaryl-CoA dehydrogenase (GCDH), acyl-CoA synthetase short-chain family member 2 (ACSS2), or short-chain acyl-CoA dehydrogenases (ACADS), its catabolism is primarily governed by ECHS1 [7]. Consequently, ECHS1 emerges as a pivotal regulator of protein crotonylation homeostasis.

Dysregulation of ECHS1 contributes to cellular metabolic dysfunction and tumorigenesis [7, 8]. ECHS1 deficiency (ECHS1D) typically manifests clinically as Leigh syndrome, which is associated with oxidative phosphorylation (OXPHOS) dysfunction [9, 10]. This presentation contrasts with classic fatty acid oxidation disorders, yet the underlying mechanistic basis remains obscure. The activity of ECHS1 is inhibited by acetylation at K101 or K204 under eutrophic conditions, stimulating its further ubiquitination and degradation [3, 11]. Downregulation of ECHS1 level results in accumulation of fatty acids and BCAAs, activating PI3K-Akt-mTOR signaling pathway and promoting growth of renal cell carcinoma, hepatocellular carcinoma, colon cancer, gastric cancer, and glioblastoma cells [3, 7, 12]. However, the mechanism that activates ECHS1 to accommodate nutrient catabolic demands remains unelucidated.

The activity of metabolic enzymes is widely regulated by post-translational modifications, including small ubiquitin-like modification (SUMOylation), discovered three decades ago [13, 14]. SUMOylation entails the covalent conjugation of the C-terminal glycine of SUMO proteins to specific lysine residues on target substrates. This modification predominantly occurs within the canonical ΨKxE/D motif (where Ψ denotes a hydrophobic residue and x denotes any amino acid) [15]. Proteomic analyses of endogenous SUMO1 sites further identified KxΨ as a non-canonical consensus motif [16]. The SUMOylation cascade requires E1 (activating), E2 (conjugating), and E3 (ligating) enzymes, while deconjugation is mediated by SENP proteases [17]. SUMOylation generates a gain-of-function interaction surface, enabling modified proteins to recruit effectors harboring SUMO-interaction motifs (SIMs), thereby exerting a significant biological impact even at low stoichiometry [18, 19]. Although SUMOylation occurs in multiple cellular compartments, its role in mitochondria has only recently been characterized. Key mitochondrial substrates include SIRT3 (regulating deacetylation), Idh2 (maintaining redox homeostasis), and SDHA (modulating TCA cycle) [20–22]. Despite these advances, whether SUMOylation directly governs fatty acids or amino acids catabolism remains unknown. Furthermore, limited evidence exists for SUMOylation-mediated crosstalk between mitochondrial metabolic and nuclear epigenetic regulation, leaving a gap in understanding how this modification orchestrates organelle-wide metabolic adaptation.

Herein, we propose that nutrient-induced SUMOylation activates ECHS1 to promote crotonyl-CoA catabolism toward acetyl-CoA production. This metabolic adaptation coordinates histone crotonylation and acetylation dynamics, thereby epigenetically regulating the growth of lung cancer cells. Our findings establish a direct crosstalk between mitochondrial SUMOylation and nuclear epigenetic reprogramming, demonstrating how metabolic enzyme plasticity governs histone acylation landscapes in lung adenocarcinoma. This nutrient-responsive mechanism advances understanding of metabolic dysregulation in tumorigenesis and provides potential strategies for lung cancer diagnosis and therapy.

## Materials and methods

### Cell lines, antibodies, and primers

Human embryonic kidney cell line HEK293T and human LUAD cell line A549 were purchased from the Type Culture Collection of the Chinese Academy of Sciences, Shanghai, China. Human LUAD cell line PC-9 was a gift from Professor Jianxiu Yu, Shanghai Jiao Tong University School of Medicine. HEK293T, A549, and PC-9 cells were cultured in Dulbecco’s modified Eagle’s medium (DMEM; HyClone #SH30243.01), Ham’s F-12K medium (HyClone #SH30026.01), and RPMI-1640 medium (HyClone #SH30809.01), respectively, unless otherwise indicated in 5% CO_2_ atmosphere at 37 °C. All media were supplemented with 10% fetal bovine serum (HyClone #SH30406.05) and antibiotics (100 units/mL penicillin and 100 μg/mL streptomycin) (Yeasen #60162ES76). Low glucose DMEM was purchased from HyClone (#SH30021.01). Basal medium (#CD02KJ) and lysine/tryptophan-deprived medium (#X237KJ) were customized produced by BasalMedia company (Shanghai, China). Infected A549 cells and PC-9 cells were selected with 2 μg/mL puromycin.

Antibodies and primers are listed in Supplementary Table S1.

### ECHS1 SUMOylation assay

HEK293T cells were co-transfected with ECHS1-SBP-Flag, HA-SUMO1 and Ubc9-Myc plasmids. After 48 h, total cells were harvested and lysed with pre-cooled RIPA buffer (50 mM Tris-HCl at pH 7.4), 400 mM NaCl, 1% NP-40, 0.3% Triton X-100, 0.25% sodium deoxycholate, 0.1% SDS, 1% glycerol, 20 mM N-ethylmaleimide (NEM), 1 mM phenylmethanesulfonyl fluoride (PMSF) and EDTA-free cocktail protease inhibitor (Roche #11836170001). The cell lysate was centrifuged at 14,000 × *g* for 10 min at 4 °C, and the supernatant was incubated with anti-Flag affinity beads (Sigma #A2220) at 4 °C overnight. After the incubation, the beads were washed three times with ice-cold RIPA buffer to remove non-specifically bound substances. Then the beads were boiled in 1 × SDS loading buffer (50 mM Tris-HCl at pH6.8, 2% SDS, 10% glycerol, 0.01% bromophenol blue and 1% β-mercaptoethanol) for 10 min. The protein samples were separated by 10% SDS-PAGE and then were transferred onto a PVDF membrane, where immunoblotting was performed using the indicated antibodies. Endogenous SUMOylation assays were performed using an in-house SUMO1C-K (5C4) mouse monoclonal antibody to enrich SUMO1-modified proteins [16]. For detecting the SUMO-SIM interaction, cells were lysed in weak RIPA buffer (50 mM Tris-HCl at pH 7.4, 150 mM NaCl, 0.5% NP-40, 20 mM NEM, 1 mM PMSF and EDTA-free cocktail protease inhibitor). Myc-tagged ECHS1 was enriched using anti-Myc magnetic beads (Selleck #B26301).

### Mitochondrial fractionation

Mitochondria were isolated from the indicated cells using established methods [21]. Briefly, cultured cells were washed with pre‑cooled PBS and resuspended in ice‑cold MS buffer (210 mM mannitol, 70 mM sucrose, 1 mM EGTA, and 5 mM Tris‑HCl, pH 7.5). Fresh 1 mM PMSF, 20 mM NEM, and protease inhibitor cocktail were added to the buffer immediately before use. After 30 min of hypotonic swelling on ice, the cell suspension was homogenized 60 times using a glass-Teflon motorized homogenizer. The homogenate was centrifuged at 1000 × *g* for 10 min to remove nuclei and unbroken cells, and this low‑speed centrifugation step was repeated twice. The resulting supernatant was transferred to a new tube and centrifuged at 3500 × *g* for 10 min to collect the mitochondrial fraction.

### ECHS1 activity assay

Aliquots corresponding to 100 μg of mitochondrial protein equivalent were resuspended in 40 μL ice-cold mitochondrial lysis buffer (50 mM Tris-HCl, pH 7.4, 100 mM NaCl, 2% digitonin, 20 mM NEM, 1 mM PMSF, EDTA-free protease inhibitor cocktail), incubated on ice for 20 min, then centrifuged at 20,000 × *g* for 10 min at 4 °C. The supernatant was collected, and 100 μL aliquots containing 5 μg mitochondrial proteins in reaction buffer (50 mM Tris-HCl, pH 7.4, 100 mM NaCl, 80 μM crotonyl-CoA) were transferred to individual wells of a 96-well plate (Corning #3635). Absorbance at 263 nm was monitored every minute for 15 min at 37 °C using a microplate reader.

### Immunofluorescence

For observing the mitochondrial localization of ECHS1, immunofluorescence staining was performed and imaged with a confocal microscope. Briefly, cells were stained with Mito-Tracker Green (Byotime #C1048), fixed, permeabilized, and blocked, then incubated with the primary antibody against ECHS1 (Abnova # H00001892-D01P) overnight at 4 °C. After washing, cells were incubated with a fluorescently labeled secondary antibody (Abmart #M21014) at room temperature in the dark. Nuclei were stained with DAPI. Images were captured using the THUNDER Imager Cell Spinning Disk system (Leica), and colocalization analysis was performed to confirm the mitochondrial localization of the target protein.

### Analysis of cellular or tissue metabolites by UPLC-MS/MS

For cellular metabolites analysis: ECHS1^WT^ and ECHS1^KR^ cells (1.0 × 10^6^ per sample) were harvested, washed twice with 1 mL ice-cold PBS, and extracted with 1 mL ice-cold 80% methanol. The mixture was vortex-mixed at 1500 rpm for 30 min at 4 °C, then centrifuged at 15,000 rpm for 15 min at 4 °C. The resulting supernatant was transferred to new microcentrifuge tubes.

For tissue metabolites analysis: Fresh tissues (50 mg) were homogenized in 1 mL ice-cold 80% acetonitrile using 3 mm zirconia beads in a cryogenic grinder (60 Hz, 60 s × 2 cycles with 2 s intervals). Homogenates were vortexed at 1500 rpm for 30 min at 4 °C, centrifuged at 15,000 rpm for 15 min at 4 °C, and 500 μL of supernatant was transferred to new tubes. Samples were dried under a nitrogen stream at 37 °C and reconstituted in 50 μL ice-cold 80% acetonitrile.

Quality control and LC-MS/MS analysis: A 10 μL aliquot from each processed sample was pooled to generate quality control (QC) samples. Metabolite profiling was performed on an Agilent 1290 UHPLC system coupled to an AB Sciex QTRAP 6500+ mass spectrometer equipped with a Merck ZIC-pHILIC column (150 × 2.1 mm, 3 μm). Data acquisition in multiple reaction monitoring (MRM) mode used Analyst 1.7 software, with subsequent analysis in Sciex OS 2.1.5.

### Detection of acetyl-CoA, NAD^+^/NADH and mitochondrial ROS

Acetyl-CoA levels were quantified using the PicoProbe^TM^ Acetyl-CoA Assay Kit (BioVision #K317-100) according to the manufacturer’s instructions. NAD^+^/NADH ratios were determined with the Enhanced NAD^+^/NADH Assay Kit (Beyotime #S0176) following the manufacturer’s protocol. Mitochondrial ROS (superoxide anion) was detected with MitoSox^TM^ (Invitrogen #M36008).

### Analysis of ECHS1 polymerization and mitochondrial respiratory chain activity by blue native polyacrylamide gel electrophoresis (BN-PAGE)

Mitochondria were isolated from HEK293T, A549, or PC-9 cells. Aliquots containing 100 μg mitochondrial protein were resuspended in 40 μL ice-cold lysis buffer (1× NativePAGE Sample Buffer (Thermo Fisher #BN2003), 2% digitonin, 20 mM NEM, EDTA-free protease inhibitor cocktail), incubated on ice for 20 min, then centrifuged at 20,000 × *g* for 10 min at 4 °C. The supernatant was partitioned for parallel analyses: 15 μL for BN-PAGE and 20 μL for SDS-PAGE. Proteins from both electrophoretic systems were transferred to PVDF membranes and immunoblotted with target-specific antibodies. Activities of mitochondrial respiratory chain complex I and IV were detected using established methods [23].

### Cleavage under targets and tagmentation sequencing (CUT&Tag-seq)

For H2BK20cr and H3K27cr CUT&Tag profiling, 50,000 cells per genotype were processed using the Hyperactive Universal Kit (Vazyme #TD904) with validated anti-H2BK20cr or anti-H3K27cr antibody according to the manufacturer’s protocol. Libraries were sequenced on an Illumina NovaSeq 6000 (2 × 150 bp; Novogene). Bioinformatics analysis included: (1) Alignment to hg38/GRCh38.p13 via bowtie2 (v2.2.5; parameters: --end-to-end, --very-sensitive, --no-mixed, --no-discordant, --phred33, -X 1000); (2) Duplicate removal with sambamba (v0.8.2) and mitochondrial DNA exclusion using ATACgraph [24]; (3) Peak calling via MACS3 (v3.0.1, *q* < 0.05) with fragment distribution visualization by deepTools (v3.5.1); (4) Genomic annotation via HOMER (v4.11); (5) Differential peak identification by edgeR (|FC| ≥ 1.2, *p* < 0.05).

### RNA-sequencing (RNA-seq)

Total RNA was extracted from 1.0 × 10^6^ ECHS1-knockdown A549 cells reconstituted with ECHS1^WT^ or ECHS1^KR^ using TRIzol^TM^ (Invitrogen #15596026), with immediate snap-freezing at −80 °C prior to commercial RNA-seq processing (GENEWIZ), which included RNA extraction under RIN ≥ 8.0/DV_200_ > 50% QC standards, Illumina-compatible library construction via polyA selection-fragmentation-dscDNA synthesis-end repair/A-tailing-adapter ligation-size selection (150–200 bp)-PCR amplification, and NovaSeq 6000 sequencing (150 bp PE); subsequent bioinformatic analysis was performed to identify differential expressed genes using edgeR with under |FC| ≥ 1.2 and *p* < 0.05 thresholds. Gene set enrichment analysis (GSEA) was performed using clusterProfiler (v4.12.6) with KEGG pathways, citrate cycle (hsa00020), fatty acid degradation (hsa00071), oxidative phosphorylation (hsa00190), and PI3K-AKT signaling (hsa04151), evaluating metabolic alterations in ECHS1^KR^ versus ECHS1^WT^ cells under |NES| > 1.5 and *p* < 0.05 thresholds.

### Quantitative RT-PCR

Total RNA was isolated from A549 cells using TRIzol^TM^ reagent (TIANGEN #DP424) according to the manufacturer’s protocol. From 1 μg DNase-treated RNA, cDNA was synthesized with HiScript III All-in-one RT SuperMix (Vazyme #R333-01) under the following conditions: 50 °C for 15 min, 85 °C for 5 s. Quantitative PCR (q-PCR) was performed in triplicate using ChamQ Universal SYBR qPCR Master Mix (Vazyme #Q711-02) on the LightCycler 96 system (Roche). Gene expression was normalized to human 18S rRNA via the 2^−ΔΔCt^ method, with primer sequences provided in Supplementary Table S1.

### Cell proliferation assay

Cells were seeded in 6-well plates at 20,000 cells/well and cultured in the indicated medium for 24, 48, 72, and 96 h. Cells were digested and resuspended in an appropriate volume of culture medium. The cell suspension was gently mixed, and a small volume was transferred to a sterile centrifuge tube. An equal volume of 0.4% trypan blue staining solution (Invitrogen #T10282) was added, followed by gentle mixing, and the mixture was incubated at room temperature for 5 min. Viable cells were counted using a Countess II Automated Cell Counter (Thermo Fisher Scientific). All experiments were performed with three biological replicates.

### Colony formation assay

Cells were plated in 6-well plates at 500 cells/well and cultured for 14 days in a humidified 37 °C, 5% CO_2_ incubator. Colonies were fixed with 4% paraformaldehyde for 15 min, stained with 0.5% crystal violet (Beyotime #C0121) for 20 min, and washed three times with PBS. Colony images were captured, and the number of colonies with a diameter larger than 200 μm was quantified using ImageJ V1.8.0 (NIH, USA). Each experiment was performed in triplicate.

### Transplanted tumor model in nude mice

Five-week-old male BALB/c nude mice (Shanghai Lingchang Bio.) were maintained under specific pathogen-free conditions and randomly assigned to either a normal diet or a lysine/tryptophan-restriction diet (K/W-R diet; 90% of lysine and 90% of tryptophan reduction). Subcutaneous xenografts were established by injecting 3 × 10^6^ A549 cells (ECHS1^WT^ or ECHS1^KR^) into the right flank (*n* = 6/group), with tumor volume measured every 48-h using digital calipers and calculated as *V* = (length × width^2^)/2 (length: longest axis; width: perpendicular axis); mice were euthanized at day 21 post-inoculation for tumor excision and weighing, with all procedures compliant with ARRIVE 2.0 guidelines and approved by Shanghai Jiao Tong University School of Medicine.

### Histologic evaluation

Immunohistochemical staining of human lung adenocarcinoma tissue microarrays (WeiaoBio #ZL-LUC1601) was performed using antibodies against H2BK20cr and H3K27cr at magnifications of 4× (scale bar = 312.5 μm) and 40× (scale bar = 25 μm). Protein expression was quantified via H-score (range 0-300) calculated as [1 × (% low-expressing cells) + 2 × (% medium-expressing cells) + 3 × (% high-expressing cells)]. Based on the staining intensity, samples were classified into four groups: the weak group (+), moderate group (++), strong group (+++), and strongest group (++++) [25]. Statistical analysis utilized GraphPad Prism 10.0 for H-score comparisons and Kaplan–Meier Plotter (v2024; www.kmplot.com) for survival analysis.

### Statistical analysis

Statistical methods for all figures are specified in either the methods section or the respective figure legends.

## Results

### ECHS1 is a SUMOylated protein

**Fig. 1:**
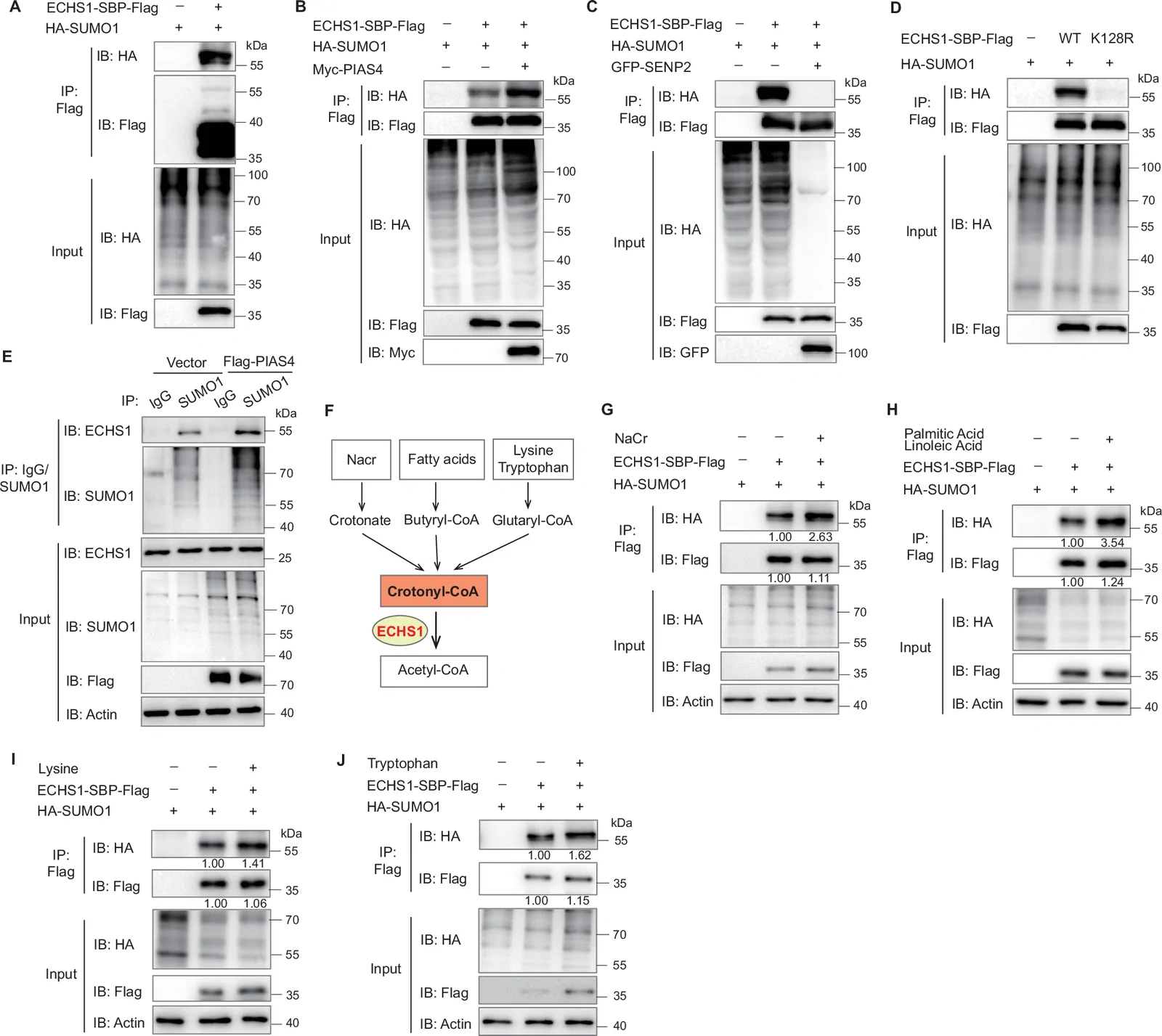
ECHS1 is modified by SUMO1. **A**–**D** HEK293T cells were co-transfected with the indicated plasmids. Total cell lysates were collected and subjected to the SUMOylation assay. **E** Mitochondria of HEK293T cells transfected with or without Flag-tagged PIAS4 were separated and subjected to an endogenous SUMOylation assay. **F** Sketch map of crotonyl-CoA metabolism. **G** Transfected HEK293T cells were cultured in basal medium for 24 h and then treated with or without 1 mM sodium crotonate for 5 min. Total cell lysates were collected and subjected to the SUMOylation assay. **H** Transfected HEK293T cells were cultured in basal medium for 24 h and then treated with or without 0.05 mM palmitic acid and linoleic acid for 5 min. Total cell lysates were collected and subjected to the SUMOylation assay. **I** Transfected HEK293T cells were cultured in basal medium for 24 h and then treated with or without 2 mM lysine for 30 min. Total cell lysates were collected and subjected to the SUMOylation assay. **J** Transfected HEK293T cells were cultured in basal medium for 24 h and then treated with or without 0.2 mM tryptophan for 5 min. Total cell lysates were collected and subjected to the SUMOylation assay.

SUMOylation has emerged as a key post-translational modification to regulate enzymatic activity [26, 27]. Previously, we identified an association between ECHS1 and SUMO1 in HepG2 cells [28]; however, whether ECHS1 undergoes SUMO1 modification remained unknown. To test whether ECHS1 is a SUMOylated protein, we performed co-immunoprecipitation (co-IP) followed by Western blotting. Lysates from HEK293T cells expressing SBP-Flag-ECHS1 and HA-SUMO1 were immunoprecipitated using an anti-Flag antibody. In addition to free ECHS1 (~37 kDa), a ~57 kDa band was enriched and recognized by both anti-Flag and anti-HA antibodies, indicating the presence of SUMO1-conjugated ECHS1 (Fig. 1A). To confirm this band represented SUMOylated ECHS1, we modulated SUMOylation by co-expressing key regulators. Co-expression of the E3 ligase PIAS4 enhanced the band intensity, whereas the deSUMOylase SENP2 eliminated it (Fig. 1B, C). Next, we mapped the SUMOylation site. As none of the lysine residues in ECHS1 conform to the canonical ΨKxE/D motif, we therefore screened alternative consensus sequences and identified K128 as matching the KxΨ motif (Supplementary Fig. S1). Mutation of this residue to arginine (K128R) markedly diminished SUMOylated ECHS1, establishing K128 as the primary SUMOylation site (Fig. 1D). Endogenous co-immunoprecipitation (co-IP) assays further confirmed that ECHS1 undergoes SUMO1 modification in mitochondria, and transfection of PIAS4 enhanced the intensity of the SUMOylated ECHS1 band (Fig. 1E). Collectively, these results demonstrate that ECHS1 is a SUMOylated protein.

We next investigated whether ECHS1 SUMOylation responds to nutrient stimuli. As crotonyl-CoA, the highest-affinity substrate of ECHS1, can be generated from sodium crotonate, fatty acids, lysine, or tryptophan (Fig. 1F), we supplemented cells with these nutrients. SUMOylated ECHS1 was rapidly stimulated by these nutrients (Fig. 1G–J). This nutrient-induced enhancement suggests that ECHS1 SUMOylation might play a role in mediating adaptive cellular responses to nutrient utilization.

### SUMOylation enhances the activity of ECHS1

**Fig. 2:**
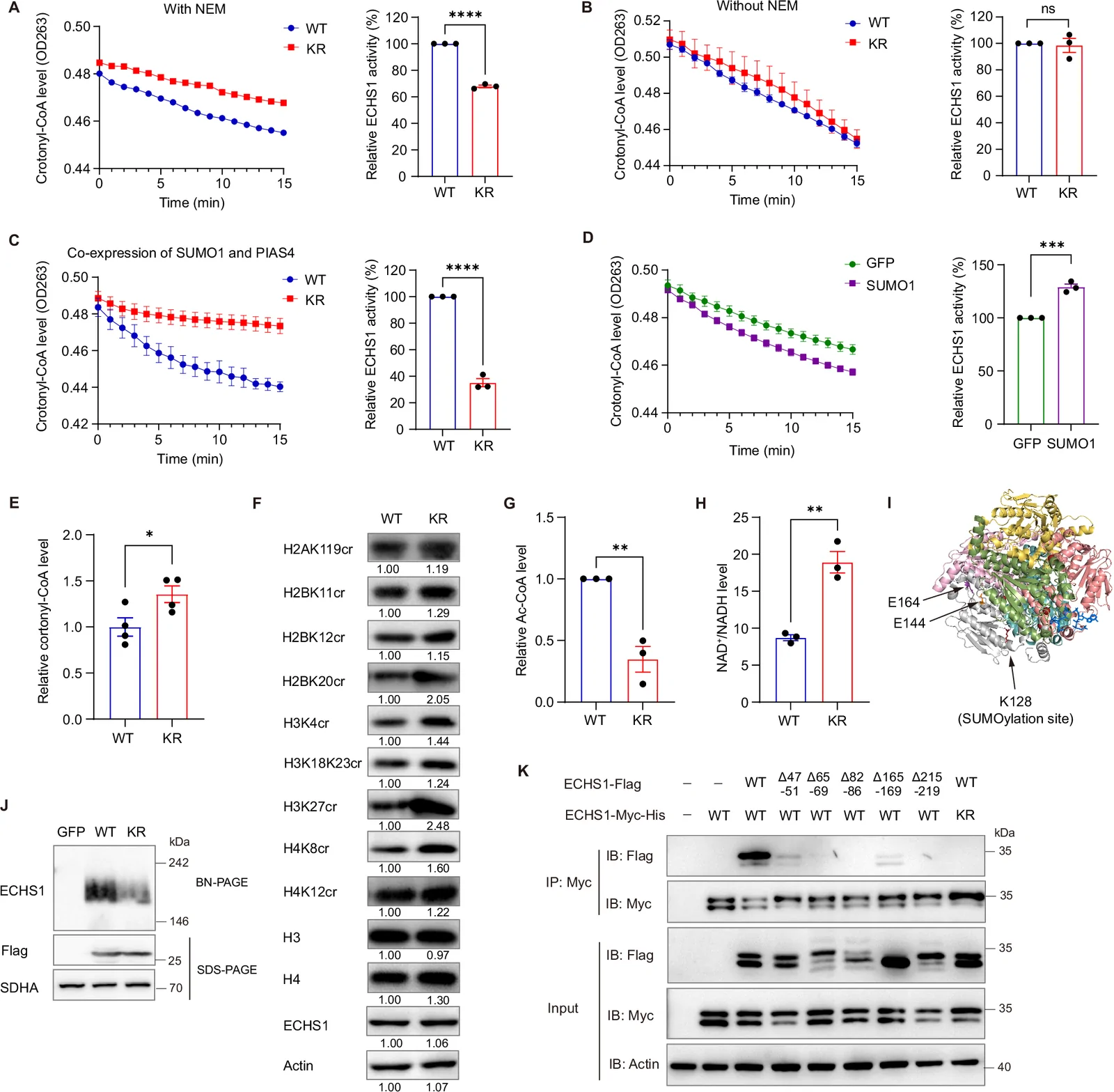
SUMOylation enhances the activity of ECHS1. **A** HEK293T-shECHS1 cells were transfected with wild type (ECHS1^WT^) or K128R-mutated ECHS1 (ECHS1^KR^). Mitochondria of these cells were separated and lysed in a buffer containing 20 mM NEM. **B** Mitochondria of HEK293T-ECHS1^WT^ and -ECHS1^KR^ cells were separated and lysed in buffer without NEM. **C** HEK293T-ECHS1^WT^ and -ECHS1^KR^ cells were co-transfected with HA-SUMO1 and Flag-PIAS4. Mitochondria of these cells were separated and lysed in a buffer containing 20 mM NEM. **D** HEK293T cells were transfected with Flag-tagged SUMO1 or GFP. Mitochondria of these cells were separated and lysed in a buffer containing 20 mM NEM. Activity of ECHS1 was indicated by the degradation rate of crotonyl-CoA. Error bars represent the SEM values (*n* = 3). **E** Intracellular levels of crotonyl-CoA of HEK293T cells expressing wild-type or K128R-mutated ECHS1 were quantified by LC-MS. Error bars represent the SEM values (*n* = 4). **F** Total cell lysate of HEK293T cells expressing wild-type or K128R-mutated ECHS1 was subjected to Western blotting with the indicated antibodies. Relative intensities of protein bands were analyzed by ImageJ software. **G** Intracellular levels of acetyl-CoA of HEK293T cells expressing wild-type or K128R-mutated ECHS1 were detected in three independent experiments. Error bars represent the SEM values (*n* = 3). **H** Intracellular levels of NAD^+^ and NADH of HEK293T cells expressing wild-type or K128R-mutated ECHS1 were detected in three independent experiments. NAD^+^/NADH was calculated. Error bars represent the SEM values (*n* = 3). **I** Homohexamer structure (PDB: 2hw5) of ECHS1 was demonstrated with six colors, indicating different monomers. Active sites (E144&E164) and SUMOylation site (K128) of the gray monomer were annotated. **J** HEK293T-shECHS1 cells were transfected with Flag-tagged wild-type or K128R-mutated ECHS1. Mitochondrial proteins were separated by BN-PAGE or SDS-PAGE and detected with the indicated antibodies. **K** HEK293T cells were transfected with Myc-tagged ECHS1 and Flag-tagged ECHS1 truncates. Co-immunoprecipitation (Co-IP) was performed using anti-Myc magnetic beads in weak RIPA buffer. Error bars represent SEM values; statistical significance (unpaired *t*-test): ****p < 0.0001, ***p < 0.001, **p < 0.01, *p < 0.05, ns non-significant.

To determine the functional impact of SUMOylation on ECHS1, we mutated its SUMOylation site (K128R) and measured enzymatic activity both in vitro and in vivo. Endogenous ECHS1 in HEK293T cells was stably knocked down and transiently reconstituted with equivalent levels of wild-type ECHS1 (ECHS1^WT^) or the K128R mutant (ECHS1^KR^) (Supplementary Fig. S2A). Mitochondrial fractions were incubated with crotonyl-CoA in vitro. To prevent deSUMOylation during sample preparation, N-ethylmaleimide (NEM) was added to lysates to inhibit SENP activity. ECHS1 knockdown reduced enzyme activity (Supplementary Fig. S2B). As predicted, this depletion also suppressed activity in the absence of NEM supplementation (Supplementary Fig. S2C, D). In the presence of NEM, mitochondria harboring ECHS1^WT^ degraded crotonyl-CoA more efficiently than those with ECHS1^KR^ (Fig. 2A). Without NEM, the activities of ECHS1^WT^ and ECHS1^KR^ were comparable (Fig. 2B). This NEM-dependent enhancement observed specifically in ECHS1^WT^ indicated that the activity impairment in the KR mutant stems primarily from SUMOylation deficiency, not other effects of the mutation. Moreover, co-expression of SUMO1 and PIAS4 significantly boosts the activity of ECHS1^WT^ but has no effect on ECHS1^KR^ (Fig. 2C and Supplementary Fig. S2E). This manipulation increased the activity difference between WT and KR from a modest ~30% reduction (Fig. 2A) to a more substantial ~60% reduction, indicating SUMOylation is a potent activator of ECHS1 enzymatic function. Consistently, transfection with SUMO1 augmented ECHS1 activity (Fig. 2D and Supplementary Fig. S2F), demonstrating that SUMOylation enhances ECHS1-mediated crotonyl-CoA catabolism in vitro.

We next evaluated the in vivo consequences of impaired ECHS1 SUMOylation. Mass spectrometry detected significantly higher intracellular crotonyl-CoA levels in ECHS1^KR^ cells compared to ECHS1^WT^ cells (Fig. 2E). This accumulation elevated global histone crotonylation, evidenced by increased crotonylation marks on histones H2A, H2B, H3, and H4 in ECHS1^KR^ cells (Fig. 2F). Given that ECHS1 catabolizes crotonyl-CoA to acetyl-CoA, feeding into the TCA cycle to generate NADH from NAD^+^, we measured these metabolites. ECHS1^KR^ cells exhibited decreased acetyl-CoA levels and an elevated NAD^+^/NADH ratio (Fig. 2G, H), confirming that SUMOylation is essential for efficient crotonyl-CoA-to-acetyl-CoA conversion in vivo.

To elucidate the molecular mechanism of SUMOylation-dependent ECHS1 regulation, we focused on its essential homohexameric structure (di-trimer). Structural modeling positioned the SUMOylation site K128 at the monomer-monomer interface, distal from its catalytic sites (Fig. 2I), suggesting SUMO1 conjugation might regulate formation of the hexamer. Since SUMOylation often facilitates macromolecular assembly via SUMO-SIM interactions, computational analysis (GPS-SUMO) identified multiple SIMs on ECHS1 (Supplementary Fig. S2G), supporting a potential role in self-association. To test the requirement of SUMOylation for hexamer formation, we reconstituted ECHS1-depleted cells with Flag-ECHS1^WT^ or K128R mutant. Blue native PAGE analysis of mitochondrial extracts revealed a severe defect in hexamer assembly, specifically in K128R-expressing cells, compared to robust formation in WT-expressing cells (Fig. 2J). These combined findings establish that site-specific SUMOylation at K128 is indispensable for assembly of functional ECHS1 homohexamers, likely by stabilizing SUMO-SIM-mediated intermolecular interactions. For validation of this hypothesis, we then generated a series of SIM deletion mutants (Δ47–51, Δ65–69, Δ82–86, Δ165–169, Δ215–219) and tested their ability to interact with wild-type ECHS1 in co-immunoprecipitation assays. The results show that deletion of specific SIMs (notably Δ65–69, Δ82–86 in the spiral domain and Δ215–219 in the trimerization domain) dramatically impairs ECHS1 self-association (Fig. 2K). These results together indicate that SUMOylation at K128 of ECHS1 facilitates SUMO-SIM interactions between monomers, thereby promoting assembly of the active homohexamer.

To check whether SUMOylation affects ECHS1 localization, we co-stained A549 and PC-9 cells expressing either ECHS1^WT^ or ECHS1^KR^ with antibodies against ECHS1 and a mitochondrial marker (Mito-tracker Green). As shown in the Supplementary Fig. S2H, I, both ECHS1^WT^ and ECHS1^KR^ exhibit strong and overlapping localization with mitochondrial networks, confirming their mitochondrial matrix localization is not affected by the K128R mutation. To further test if enhanced SUMOylation influences this localization, we co-transfected cells with SUMO1 and its E3 ligase PIAS4. Even under these conditions, we observed no discernible difference in the mitochondrial localization of ECHS1 between WT and KR mutants. This suggests that while SUMOylation critically regulates ECHS1’s activity and oligomerization, it does not significantly alter its steady-state subcellular localization to mitochondria.

### ECHS1 SUMOylation suppresses the growth of lung cancer cells

**Fig. 3:**
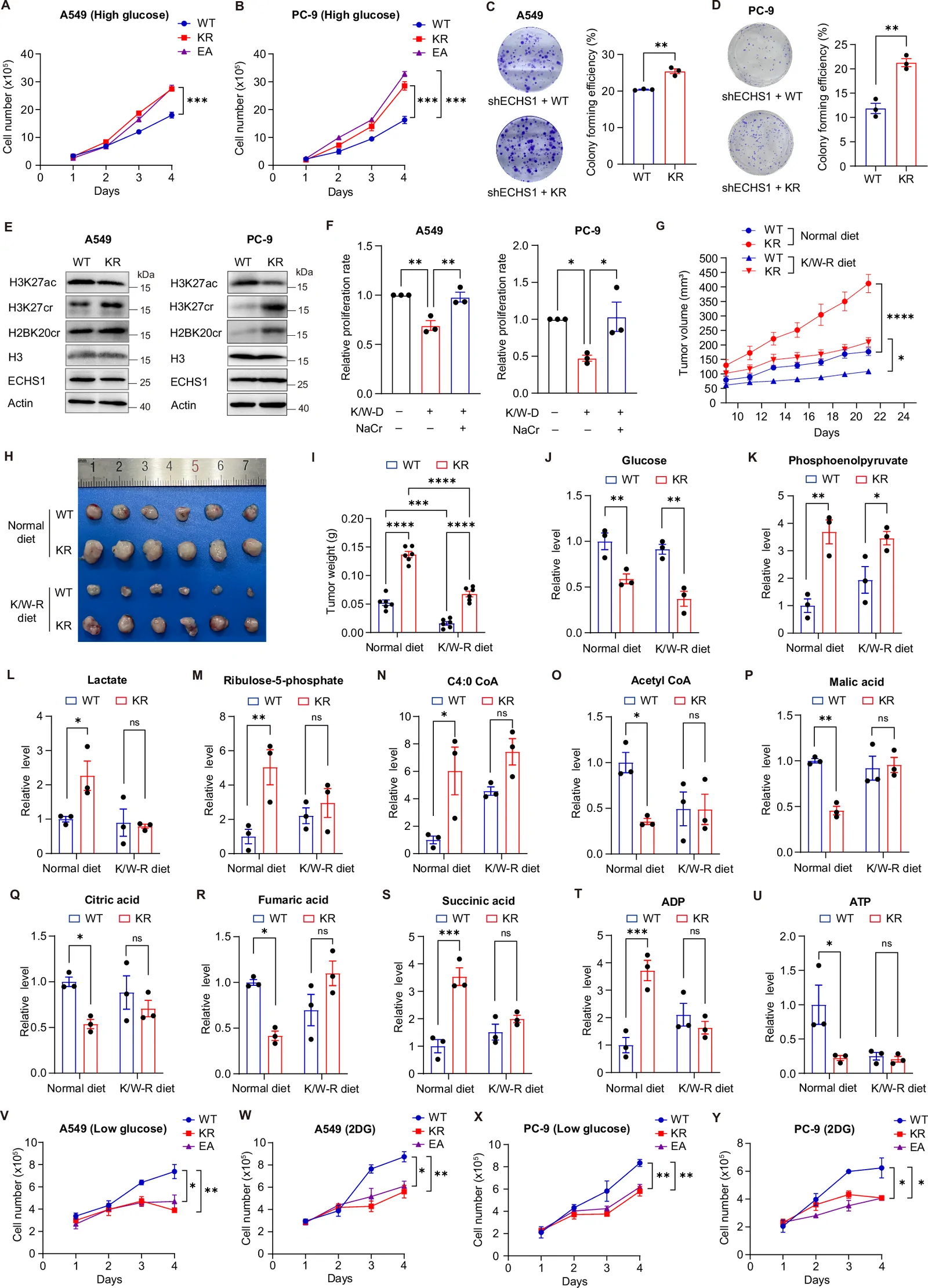
K128R mutation of ECHS1 promotes the growth of lung cancer cells. **A**, **B** Proliferation rate of A549 and PC-9 cells expressing wild type, K128R, or E144/164A mutated ECHS1 was detected by cell counting. Data on day 4 were analyzed. Error bars represent the SEM values (*n* = 3). Statistical significance (one-way ANOVA with Dunnett’s multiple comparisons test): ***p < 0.001. **C**, **D** Colony formation of A549 and PC-9 cells expressing wild-type or K128R-mutated ECHS1. Error bars represent the SEM values (*n* = 3). Statistical significance (unpaired *t*-test): **p < 0.01. **E** Proteins of A549 and PC-9 cells expressing wild-type or K128R-mutated ECHS1 were subjected to Western blotting with the indicated antibodies. **F** A549 or PC-9 cells were cultured in complete medium or lysine/tryptophan-deprived (K/W-D) medium, supplemented with or without 1 mM sodium crotonate for 3 days. Error bars represent the SEM values (*n* = 3). Statistical sig*n*ificance (one-way ANOVA with Dunnett’s multiple comparisons test): **p < 0.01, *p < 0.05. **G** The volume of subcutaneous tumors was measured at different time points. Data are represented as means ± SEM (*n* = 6 mice per group). Statistical significa*n*ce (two-way ANOVA with Šídák’s multiple comparisons test): ****p < 0.0001, *p < 0.05. **H** Photos of subcutaneous tumors of the indicated groups grown for 21 days. **I** Tumor weight of the indicated groups grown for 21 days. Data are represented as means ± SEM (*n* = 6 mice per group). Statistical significance (two-way ANOVA with Šídák’s multiple comparisons test): ****p < 0.0001, ***p < 0.001. **J**–**U** Metabolites of three mice from the indicated groups were detected by LC-MS. Error bars represent the SEM values (*n* = 3). Statistical significance (two-way ANOVA with Šídák’s multiple comparisons test): ***p < 0.001, **p < 0.01, * indicates *p* < 0.05, ns non-significant. **V**-**Y** A549 and PC-9 cells expressing wild type, K128R, or E144/164A mutated ECHS1 were cultured in low glucose medium or high glucose medium supplemented with 5 mM 2DG. Cell proliferation rate was detected by cell counting, and data on day 4 were analyzed. Error bars represent the SEM values (*n* = 3). Statistical significance (one-way ANOVA with Dunnett’s multiple comparisons test): **p < 0.01, *p < 0.05.

While ECHS1 functions as a tumor suppressor in multiple malignancies, its role in lung cancers remains unexplored. To define the biological function of ECHS1 SUMOylation, we characterized growth phenotypes in lung cancer cells. Human lung adenocarcinoma (LUAD) cell line A549 and PC-9 with stable ECHS1 knockdown were reconstituted with equivalent levels of ECHS1^WT^, ECHS1^KR^, or catalytically inactive mutant ECHS1^EA^ (Supplementary Fig. S3A, B). ECHS1^KR^-expressing cells exhibited accelerated proliferation, phenocopying the ECHS1^EA^ mutant (Fig. 3A, B), and enhanced colony formation compared to WT controls (Fig. 3C, D), indicating that SUMOylation is essential for the tumor suppressor function of ECHS1.

Given our finding that ECHS1 SUMOylation regulates crotonyl-CoA/acetyl-CoA balance to modulate histone modifications, we examined epigenetic marks in lung cancer cells. Immunoblotting revealed elevated H2BK20cr and H3K27cr alongside reduced H3K27ac in ECHS1^KR^ cells (Fig. 3E). These alterations mechanistically explain the accelerated proliferation phenotype, as H3K27cr deposition epigenetically silences proliferation suppressor genes while H3K27ac promotes their expression [5]. To directly test the functional role of crotonyl-CoA, we deprived cells of lysine and tryptophan, which are primary sources of crotonyl-CoA in cell culture medium. Lysine/tryptophan-deprivation significantly suppressed cell proliferation (Fig. 3F). This defect was rescued by sodium crotonate supplementation, establishing crotonyl-CoA as the critical metabolic mediator of lysine/tryptophan-dependent growth.

In xenograft models, A549-ECHS1^KR^ cells formed larger tumors than WT controls (Fig. 3G–I). Dietary restriction of lysine and tryptophan reduced tumor growth in both cohorts, though ECHS1^KR^ tumors remained larger under nutrient stress, suggesting compensatory crotonyl-CoA production from alternative sources (e.g., fatty acid metabolism) in vivo. These data demonstrate that ECHS1 SUMOylation constrains lung cancer progression by catabolizing crotonyl-CoA, partially derived from lysine and tryptophan metabolism. Metabolic profiling revealed that ECHS1^KR^ tumors from normal-diet mice exhibited: reduced glucose with elevated phosphoenolpyruvate, lactate and ribulose-5-phosphate (indicating enhanced glycolysis and pentose phosphate flux; Fig. 3J–M); C4-acyl-CoA accumulation with decreased acetyl-CoA (suggesting suppressed β-oxidation; Fig. 3N, O); concomitant reductions in malate, citrate and fumarate (reflecting TCA cycle impairment; Fig. 3P–R); paradoxical succinate accumulation (implying succinate dehydrogenase of respiratory chain inhibition; Fig. 3S); and increased ADP with decreased ATP levels (confirming OXPHOS deficiency; Fig. 3T, U). Collectively, these results demonstrate that disrupting ECHS1 SUMOylation reprograms cellular metabolism toward aerobic glycolysis (Warburg effect) to sustain tumor growth. Lysine/tryptophan-restriction diet attenuated most of the above metabolic differences, indicating that ECHS1 SUMOylation regulates cellular metabolism partially through crotonyl-CoA availability from these amino acids. To further determine the dependency of ECHS1^KR^ cells on glycolysis, we performed glucose starvation experiments on A549 and PC-9 cells. As shown in Fig. 3V–Y, when cells are cultured in low-glucose medium or treated with the glycolytic inhibitor 2-deoxy-D-glucose (2DG), the proliferation advantage of ECHS1^KR^ and ECHS1^EA^ cells is completely reversed. In fact, under these conditions, the mutant cells now grow more slowly than the WT cells. This “synthetic sickness” demonstrates that the hyper-proliferative state of KR/EA cells is addicted to the glycolytic shift; when glycolysis is forcibly constrained, their underlying defect in mitochondrial fuel oxidation (due to low ECHS1 activity) leaves them at a severe bioenergetic disadvantage. Furthermore, similar results were observed in HEK293T cells (Supplementary Fig. S3C–E), indicating that the growth-suppressive function of ECHS1, when activated by SUMOylation, is not a cell-type-specific phenomenon but rather reflects a more fundamental mechanism of metabolic control.

### ECHS1 SUMOylation deficiency upregulates PI3K-Akt signaling pathway and down-regulates OXPHOS

**Fig. 4:**
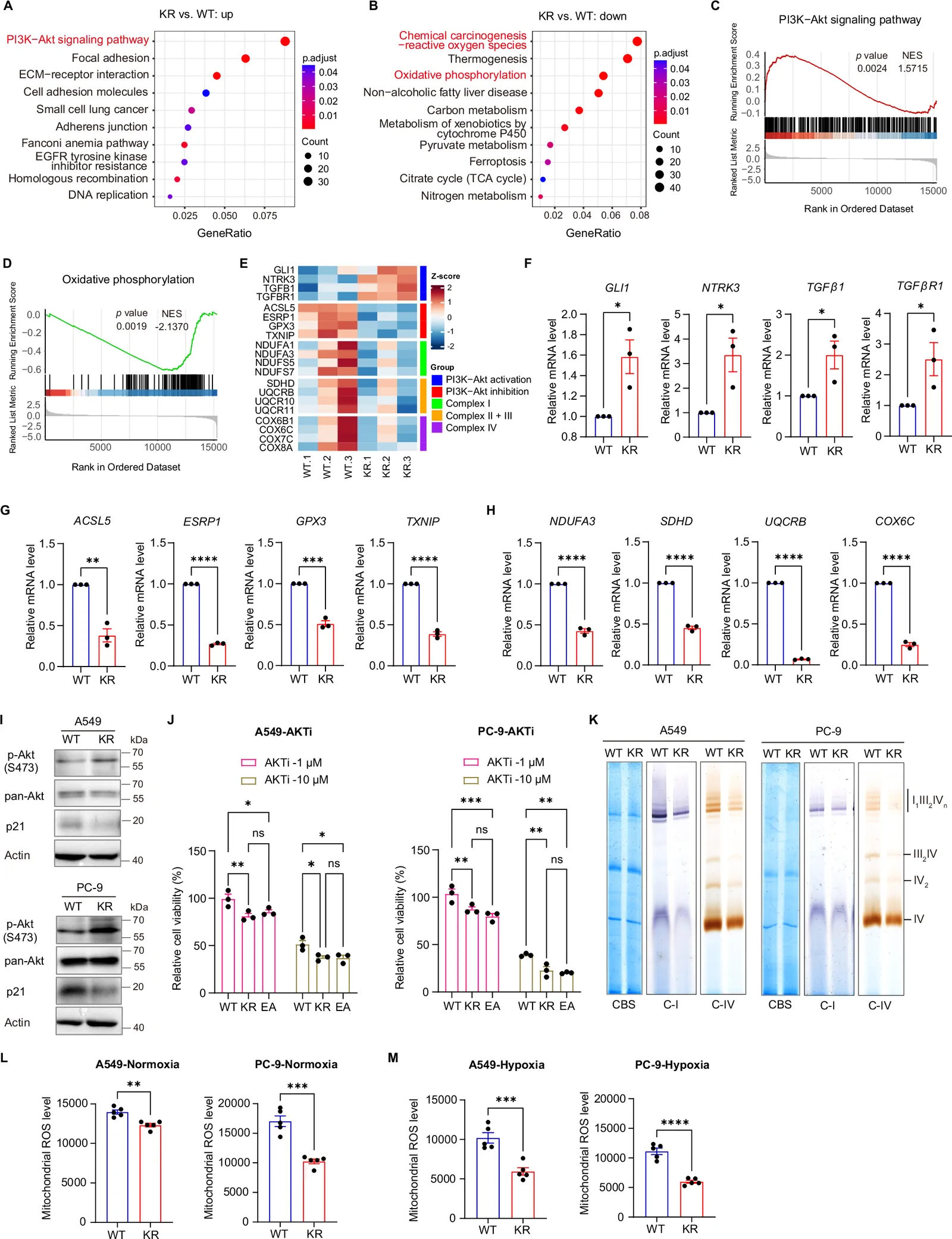
Mutation of the SUMOylation site of ECHS1 activates PI3K-Akt signaling and suppresses OXPHOS. **A** KEGG analysis of upregulated genes in ECHS1^KR^ cells versus ECHS1^WT^ controls. **B** KEGG analysis of downregulated genes in ECHS1^KR^ cells versus ECHS1^WT^ controls. **C** GSEA reveals significant enrichment of PI3K-Akt signaling pathway in ECHS1^KR^ cells versus ECHS1^WT^ controls. **D** GSEA reveals significant enrichment of OXPHOS in ECHS1^WT^ cells versus ECHS1^KR^ cells. **E** Heatmap analysis of the expression of PI3K-Akt signaling and OXPHOS-related genes. **F** Q-PCR validation of genes activating PI3K-Akt signaling. **G** Q-PCR validation of genes inhibiting PI3K-Akt signaling. **H** Q-PCR validation of respiratory chain subunits. **I** Western blotting validation of Akt phosphorylation in A549 and PC-9 cells expressing wild-type or K128R-mutated ECHS1. **J** A549 and PC-9 stable cell lines were treated with AKT inhibitor VIII (MCE # HY-10355) at the indicated concentrations for 24 h, followed by cell counting. Relative cell viability was determined relative to the number of untreated cells. Data are represented as means ± SEM (*n* = 3). Statistical significance (two-way ANOVA with Šídák’s multiple comparisons test): ***p < 0.001, **p < 0.01, *p < 0.05, ns non-significant. **K** In-gel activity analysis of respiratory chain complex I and complex IV in A549 and PC-9 cells expressing wild-type or K128R-mutated ECHS1. CBS: Coomassie blue staining. **L**, **M** Quantitative analysis of mitochondrial ROS levels in A549 and PC-9 cells expressing wild-type or K128R-mutated ECHS1, which were cultured under normoxia (21% O_2_) or hypoxia (1% O_2_). Error bars represent the SEM values (*n* = 3 in q-PCR analysis; *n* = 5 i*n* ROS measurements). Statistical significance (unpaired *t*-test): ****p < 0.0001, ***p < 0.001, **p < 0.01, *p < 0.05.

To systematically define how ECHS1 SUMOylation regulates tumor growth, we performed transcriptomic analysis of A549 cells expressing ECHS1^WT^ or ECHS1^KR^. Principal component analysis revealed distinct global transcriptomic profiles between genotypes (Supplementary Fig. S4A). Differential expression analysis identified 1167 upregulated and 1291 downregulated transcripts in ECHS1^KR^ versus ECHS1^WT^ cells (Supplementary Fig. S4B and Table S2). KEGG pathway analysis demonstrated upregulation of proliferation-associated pathways, particularly the PI3K-Akt signaling pathway (Fig. 4A), alongside downregulation of pathways involved in mitochondrial catabolism and cell death processes, including OXPHOS, TCA cycle, ROS generation, and ferroptosis (Fig. 4B). This impairment of OXPHOS aligns with metabolic alterations observed in ECHS1D-associated Leigh syndrome. Gene set enrichment analysis (GSEA) confirmed significant upregulation of PI3K-Akt signaling and downregulation of OXPHOS, fatty acid oxidation, and TCA cycle genes (Fig. 4C, D; Supplementary Fig. S4C, D). These transcriptomic alterations corroborate our metabolic profiling, indicating K128R-mediated ECHS1 inactivation promotes a Warburg phenotype through suppressed mitochondrial catabolism and enhanced anabolic signaling.

q-PCR validation of RNA-seq findings confirmed ECHS1^KR^ upregulates PI3K-Akt activators (*GLI1*, *NTRK3*, *TGFB1*, *TGFBR1*) while downregulating proliferation inhibitors (*ACSL5*, *ESRP1*, *GPX3*, *TXNIP*) and OXPHOS complex subunits (Fig. 4E–H; Supplementary Fig. S4E) [29–37]. Functional validation demonstrated elevated Akt-S473 phosphorylation in ECHS1^KR^-expressing A549 and PC-9 cells, accompanied by downregulation of p21, a downstream effector negatively regulated by PI3K-Akt signaling (Fig. 4I) [38]. To further determine the role of PI3K-Akt activation, we treated cells with an Akt inhibitor. The results show that while the inhibitor slows the growth of all cell lines, the proliferation of KR and EA cells is significantly more sensitive to Akt inhibition than that of WT cells (Fig. 4J). This indicates that the enhanced growth of the mutant cells is particularly dependent on the heightened Akt signaling we documented.

For validation of respiratory chain suppression, we performed in-gel activity assays to detect Complex I and IV activities. In cells, Complex I predominantly assembles into higher-order supercomplexes, with the major active form being respirasomes (I_1_III_2_IV_n_). Our results showed reduced Complex I activity within respirasomes in A549 and PC-9 cells expressing ECHS1^KR^ compared to ECHS1^WT^. Similarly, Complex IV activity, assessed in its monomeric form and within supercomplexes (IV_2_, III_2_IV, I_1_III_2_IV_n_), was diminished in ECHS1^KR^ cells (Fig. 4K). As a result, mitochondrial superoxide anion, which is primarily generated from the respiratory chain, was reduced in ECHS1^KR^ cells under normoxia (Fig. 4L). Under hypoxia, ECHS1^KR^ cells still exhibit a significant reduction in mitochondrial ROS compared to WT cells, and the magnitude of this difference is greater than that observed under normoxia (Fig. 4M). This suggests that the regulatory effect of ECHS1 SUMOylation on ROS generation is not only maintained but may be more potent in a more physiologically relevant hypoxic condition. Collectively, these findings reveal that SUMOylation-deficient ECHS1 activates oncogenic PI3K-Akt signaling while suppressing mitochondrial respiration and concomitant ROS generation, ultimately driving dysregulated proliferation.

### ECHS1 SUMOylation regulates the PI3K-Akt signaling pathway and OXPHOS through histone crotonylation

Next, we investigated how ECHS1 SUMOylation transcriptionally regulates PI3K-Akt signaling and OXPHOS-related genes. Elevated histone crotonylation in ECHS1^KR^ cells pointed to SUMOylation-dependent epigenetic control. We therefore performed CUT&Tag sequencing in A549-ECHS1^WT^ and ECHS1^KR^ cells to determine whether histone crotonylation directly modulates the transcription of these differentially expressed genes. H2BK20cr and H3K27cr were selected as representative marks given their most significant elevation in ECHS1^KR^ cells.

**Fig. 5:**
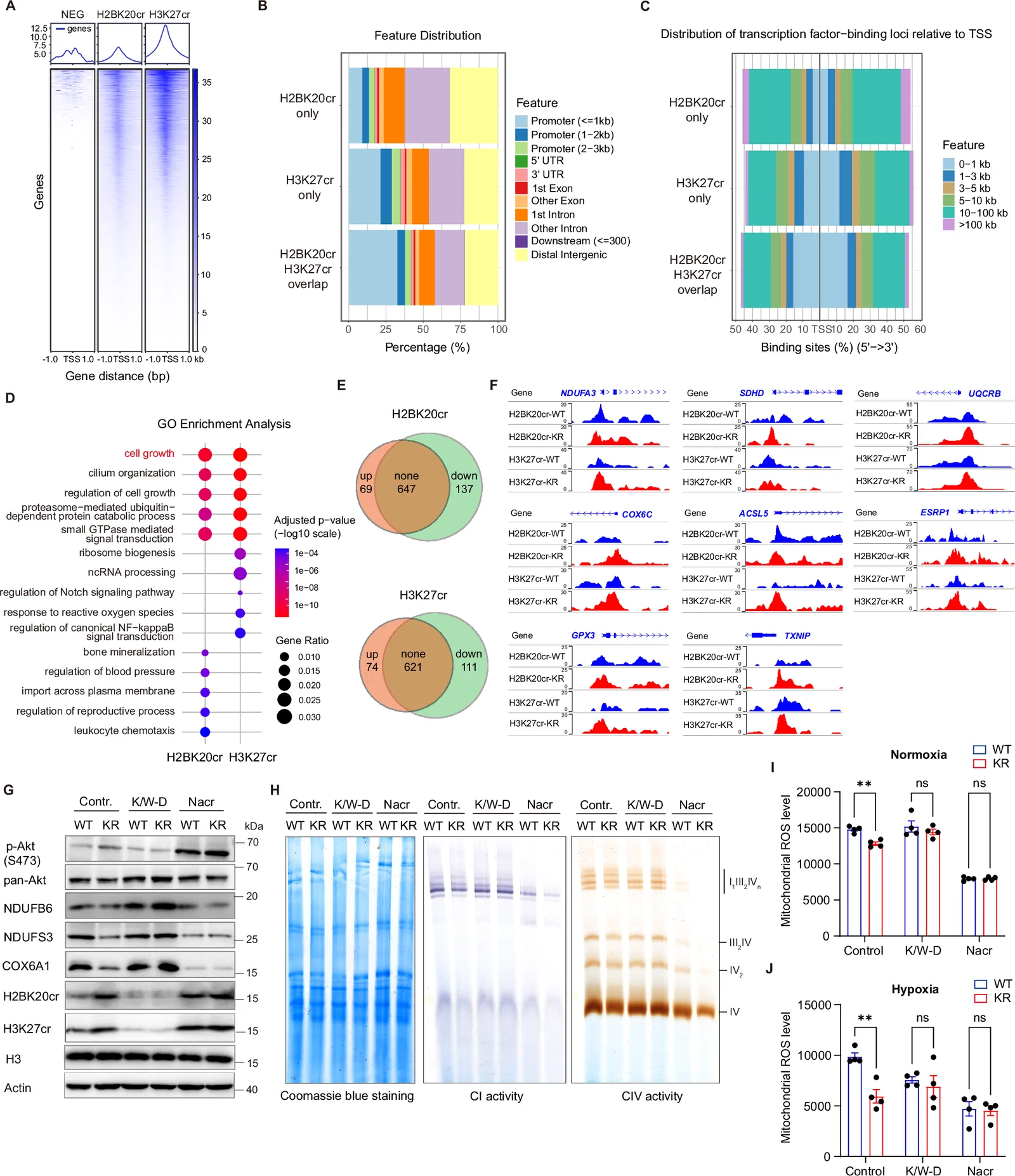
PI3K-Akt signaling and OXPHOS are epigenetically regulated by histone crotonylation. **A** CUT&Tag analysis of binding sites of H2BK20cr and H3K27cr in A549 cells. **B**, **C** Distribution of H2BK20cr and H3K27cr in genome. **D** Gene oncology (GO) analysis of the biological processes of genes specifically bond to H2BK20cr or H3K27cr. **E** Integrative genomic analysis of H2BK20cr and H3K27cr regulated genes from RNA-seq data and CUT&Tag data in ECHS1^WT^ and ECHS1^KR^ cells. **F** Enrichment of H2BK20cr or H3K27cr in promoters of PI3K-Akt signaling suppressor genes and respiratory chain subunits coding genes. **G** A549-ECHS1^WT^ and -ECHS1^KR^ cells were cultured in medium with or without lysine and tryptophan or supplemented with 1 mM sodium crotonate. Cell lysates were subjected to Western blotting and detected with indicated antibodies. **H** A549-ECHS1^WT^ and -ECHS1^KR^ cells were cultured in medium with or without lysine and tryptophan or supplemented with 1 mM sodium crotonate. Mitochondrial proteins were subjected to in-gel activity analysis of respiratory chain complex I and complex IV. **I**, **J** Mitochondrial ROS levels were measured in A549-ECHS1^WT^ and A549-ECHS1^KR^ cells cultured in medium that was either unsupplemented, supplemented with 1 mM sodium crotonate, or deprived of lysine and tryptophan, under normoxia (21% O₂) or hypoxia (1% O₂). Error bars represent the SEM values (*n* = 4). Statistical significance (two-way ANOVA with Šídák’s multiple comparisons test): **p < 0.01, ns non-significant.

As histone crotonylation remains poorly studied in lung cancer, we first characterized H2BK20cr and H3K27cr binding patterns in A549 cells (Fig. 5A). Both modifications occupied promoter regions, with H3K27cr showing stronger enrichment near transcription start sites (Supplementary Fig. S5A, B). While H2BK20cr peaks predominantly localized to introns and distal intergenic regions, 40% of co-occupied peaks resided in promoters (Fig. 5B, C). Functional annotation revealed a significant overlap in biological processes regulated by H2BK20cr and H3K27cr, with 54.7% of enriched processes being shared (Supplementary Fig. S5C, D and Table S3). These core shared processes primarily involved cell growth and its regulation (Fig. 5D). H3K27cr specifically enriched for ribosome biogenesis and ncRNA processing genes, whereas H2BK20cr uniquely targeted nutrient metabolism regulators, including bone mineralization and transmembrane transport genes. These findings indicate that H2BK20cr and H3K27cr cooperatively modulate growth pathways under metabolic stress.

Integration of RNA-seq and CUT&Tag-seq data from ECHS1^KR^ vs. ECHS1^WT^ cells demonstrated H2BK20cr negatively regulated 137 transcripts while positively regulating 69. Similarly, H3K27cr repressed 111 transcripts and activated 74 (Fig. 5E). This overall transcriptional repressive effect of histone crotonylation aligns with prior observations in HCT116 cells [5]. Crucially, both marks showed enhanced enrichment at promoters of PI3K-Akt signaling suppressors and OXPHOS subunit genes in ECHS1^KR^ cells (Fig. 5F and Supplementary Fig. S5E). Corresponding mRNA reductions confirmed transcriptional repression (Fig. 4F–H), establishing that ECHS1 SUMOylation modulates these pathways by histone crotonylation.

Functional validation via crotonyl-CoA perturbation revealed that lysine/tryptophan deprivation reduced Akt phosphorylation while increasing OXPHOS subunit expression, abolishing phenotypic differences between ECHS1^WT^ and ECHS1^KR^ cells (Fig. 5G). Conversely, sodium crotonate enhanced Akt phosphorylation and suppressed OXPHOS subunit expression. Consistent with these findings, depleting crotonyl-CoA via lysine/tryptophan deprivation modestly increased Complex I and IV activities in ECHS1^KR^ cells, nearly abolishing the difference from WT cells. Conversely, supplementing the medium with sodium crotonate dramatically suppressed the activity of Complex I within respirasomes and of Complex IV across its various supercomplex assemblies (Fig. 5H). Differential mitochondrial ROS levels between ECHS1^WT^ and ECHS1^KR^ cells cultured under either normoxia or hypoxia were also abrogated by lysine/tryptophan deprivation or sodium crotonate supplementation (Fig. 5I, J). These data demonstrate that ECHS1 SUMOylation couples PI3K-Akt activation to OXPHOS suppression through histone crotonylation.

### Elevated H2BK20cr and H3K27cr levels are associated with poor prognosis in LUAD patients

**Fig. 6:**
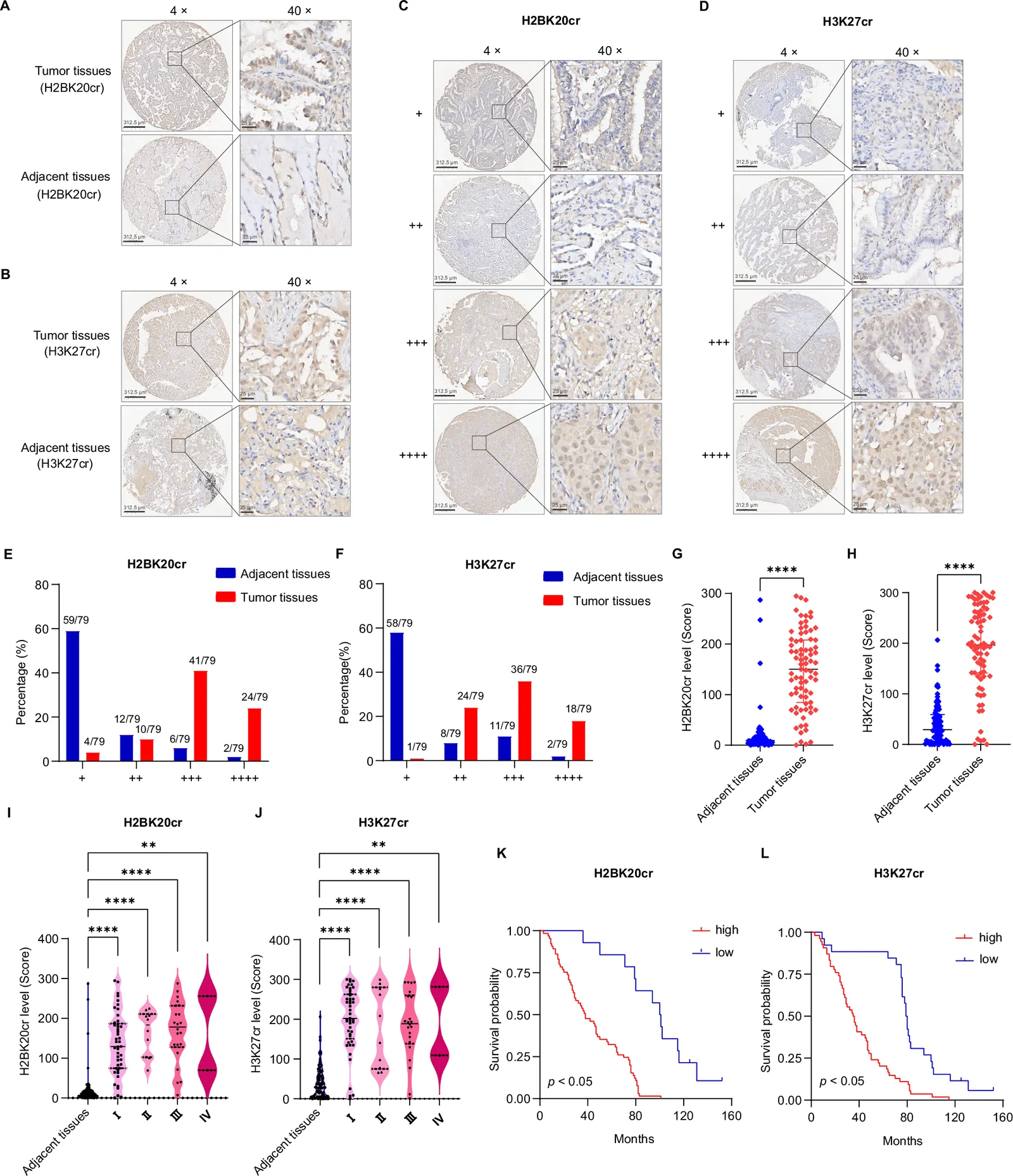
H2BK20cr and H3K27cr are highly expressed in tumor tissues of lung adenocarcinoma patients. **A**, **B** H2BK20cr and H3K27cr levels were detected by immunohistochemical staining in tumors and adjacent tissues of LUAD patients. **C**, **D** Tissue arrays were stained for detecting H2BK20cr and H3K27cr expression. Stained tissue samples were classified into four groups (+ to ++++) according to the staining intensity of pulmonary epithelial cells. **E**, **F** Distribution of H2BK20cr and H3K27cr intensities in each tumor and ssadjacent samples. **G**, **H** Quantitative analysis of H2BK20cr and H3K27cr intensities in tumor and adjacent samples. Statistical significance (paired *t*-test): ****p < 0.0001. **I**, **J** H2BK20cr and H3K27cr levels in adjacent tissues and different stages of AJCC tumor tissues. Statistical significance (one-way ANOVA with Dunnett’s multiple comparisons test): ****p < 0.0001, **p < 0.01. **K**, **L** The Kaplan–Meier curves analysis of the overall survival rate of LUAD patients according to the expression level of H2BK20cr or H3K27cr with the log-rank test.

As a readout of ECHS1 SUMOylation, histone crotonylation has not been clinically associated with lung cancer. To assess the clinical significance, we analyzed H2BK20cr and H3K27cr expression in tissue microarrays containing 79 paired primary LUAD specimens and adjacent normal tissues (Fig. 6A, B). Based on immunohistochemical staining intensity of pulmonary epithelial cells, samples were categorized into four ordinal groups (negative to strong) (Fig. 6C, D). While 74.7% (59/79) of adjacent tissues showed minimal H2BK20cr and 73.4% (58/79) exhibited minimal H3K27cr staining, 82.3% (65/79) of tumors displayed strong H2BK20cr and 68.3% (54/79) showed strong H3K27cr expression (+++/++++ groups; Fig. 6E, F). Quantitative analysis confirmed significantly higher H2BK20cr and H3K27cr levels in tumors versus adjacent tissues (Fig. 6G, H). Notably, this elevation was already evident in American Joint Committee on Cancer (AJCC) stage I tumors (Fig. 6I, J), indicating their potential as early diagnostic biomarkers. Kaplan–Meier analysis revealed significantly reduced overall survival in patients with high versus low H2BK20cr or H3K27cr expression (Fig. 6K, L). These findings establish histone crotonylation as both a tumor-associated epigenetic alteration and an independent prognostic factor in LUAD.

## Discussion and conclusion

**Fig. 7:**
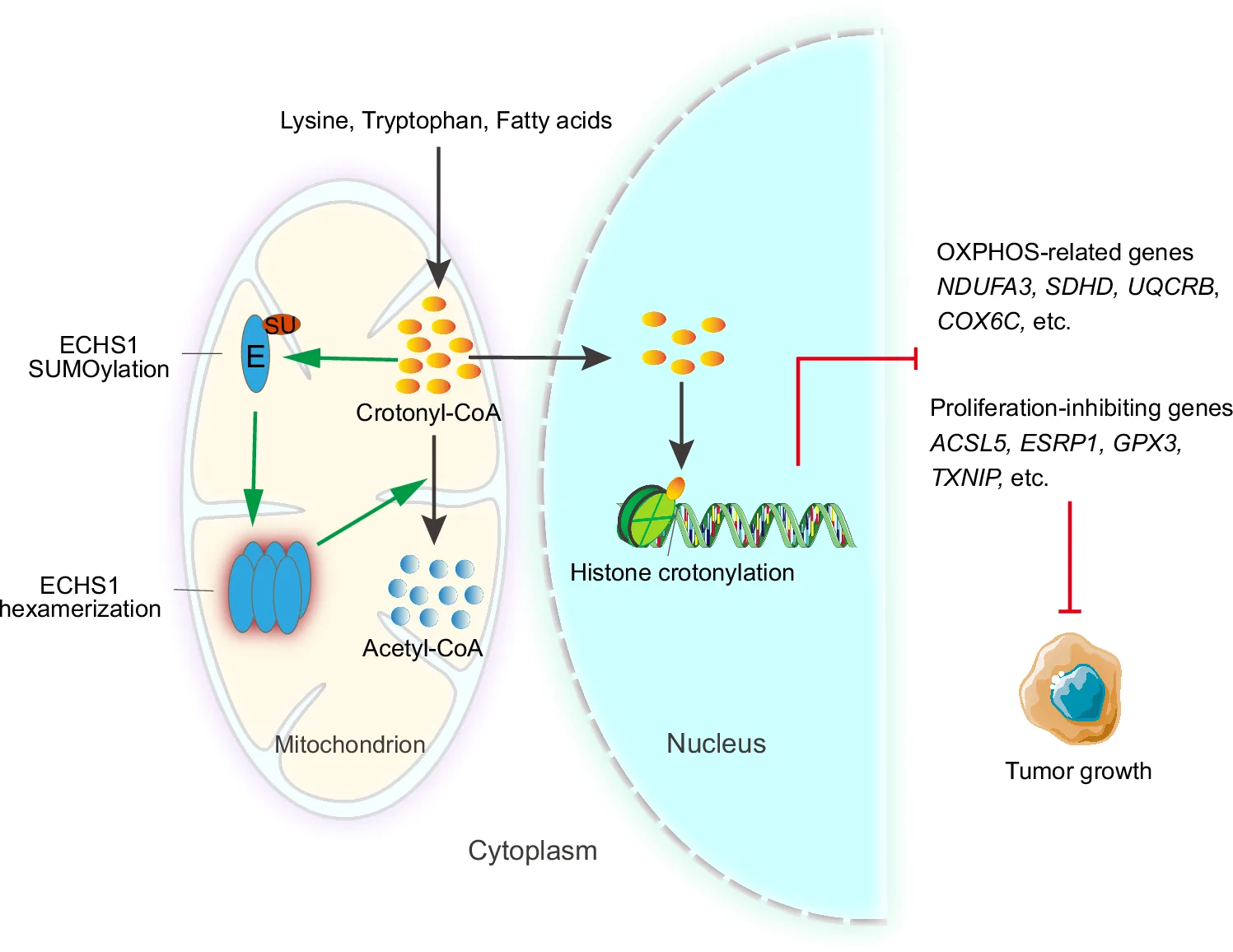
Working model of ECHS1 SUMOylation regulating histone crotonylation. Nutrients stimulate SUMOylation of ECHS1 to form a catalytic homohexamer in mitochondria, thereby accelerating crotonyl-CoA catabolism to acetyl-CoA. Diminished crotonyl-CoA pool results in reduced histone crotonylation, which promotes cancer cell growth through epigenetic regulation.

This study reveals a nutrient-adaptive mechanism wherein SUMOylation activates ECHS1 to catabolize crotonyl-CoA, thereby suppressing LUAD proliferation through reducing histone crotonylation (Fig. 7). Our findings establish a crosstalk between post-translational modifications that integrates mitochondrial metabolism with nuclear epigenetic regulation, suggesting potential diagnostic and therapeutic strategies for lung cancer.

We demonstrate that ECHS1 is activated by SUMOylation to adaptively regulate nutrient catabolism. ECHS1 functions as an essential sensor of overnutrition that antagonizes tumorigenesis [3]. While previous studies focused primarily on ECHS1 expression levels in cancer progression, we reveal its dynamic post-translational regulation [7, 39]. Nutrient exposure rapidly induces SUMOylation within 30 min, contrasting with the delayed acetylation-mediated degradation observed after ~4 h [3, 11]. This temporal hierarchy suggests SUMOylation represents an immediate adaptive response to nutrient stress, whereas acetylation signals eventual functional compromise. Supporting this, inactivation of ECHS1 via SUMOylation site mutation phenocopied the effects of ECHS1 downregulation or acetylation reported previously [3]. Mechanistically, SUMOylation at K128 promotes active homohexamer assembly, whereas acetylation near catalytic sites impairs substrate binding [3]. This assembly-factor function mirrors SUMOylation paradigms in nuclear complexes, representing a previously unrecognized regulatory mechanism for mitochondrial metabolic enzymes [18].

Our work establishes a functional link between SUMOylation and fatty acid/amino acid catabolism in mitochondria. While proteomic studies have identified numerous SUMOylated mitochondrial proteins [16], only a few of them have been mechanistically characterized, such as SIRT3 in fasting adaptation [20], Idh2 during oxidative stress [21], or SDHA under glutamine deprivation [22]. We demonstrate that ECHS1 SUMOylation activates crotonyl-CoA catabolism from both fatty acid and amino acid precursors. SUMOylation deficiency triggers PI3K-Akt/mTOR-driven anabolism while suppressing β-oxidation, TCA cycle, and OXPHOS, ultimately disrupting cellular metabolic homeostasis. The glycolytic shift and concomitant Akt activation are not just correlative but are essential drivers of the proliferation phenotype caused by defective ECHS1 SUMOylation. The tumor metabolic fragility caused by ECHS1 inactivation may serve as a potential target for glycolysis inhibitors, thereby enabling more effective tumor treatment. This regulatory axis involves dynamic control by PIAS4 and SENP2, though their mitochondrial targeting mechanisms require further investigation.

Crucially, we identify cross-compartmental crosstalk between mitochondrial SUMOylation and nuclear histone modifications. By converting crotonyl-CoA to acetyl-CoA, SUMOylated ECHS1 reduces histone crotonylation (H2BK20cr/H3K27cr) while increasing acetylation (H3K27ac), modifications with opposing transcriptional effects [5]. This substrate-level regulation resolves a key paradox: how shared modifying enzymes yield distinct histone marks. Unlike nuclear crotonyl-CoA hydratase CDYL, which degrades crotonyl-CoA without changing acetyl-CoA production [40], mitochondrial ECHS1 coordinates with β-hydroxybutyryl-CoA dehydrogenase to generate acetyl-CoA, thereby regulating the crotonyl-CoA/acetyl-CoA ratio that determines modification balance. Our findings align with studies demonstrating that metabolic enzymes influence epigenetics by controlling substrate availability for histone modifications [41]. This mechanism may illuminate ECHS1 deficiency-associated Leigh syndrome (ECHS1D), as mutation hotspots occur within SIMs, potentially impairing SUMOylation functionality [42–44]. Moreover, while this study focuses on histone modifications, ECHS1 SUMOylation may also influence non-histone protein acetylation and crotonylation, a mechanism warranting further investigation.

Our study elucidates a substrate-level regulatory mechanism for protein crotonylation, wherein nutrient availability governs crotonyl-CoA pools through the SUMOylation and activation of the mitochondrial enzyme ECHS1. This pathway operates distinctly from the known enzyme-level regulation mediated by oxygen tension, such as the hypoxia-induced downregulation of the decrotonylase HDAC6, which elevates global crotonylation [45]. These two regulatory layers are not mutually exclusive. They can function independently, as evidenced by the enhanced crotonylation observed in ECHS1^KR^ cells under normoxia. More significantly, they can act synergistically: under hypoxic conditions, the accumulation of crotonyl-CoA resulting from impaired ECHS1 activity is likely channeled more efficiently into modification reactions due to the coincident reduction in HDAC6-mediated removal. This synergy is supported by our experimental finding that the biological effects of the ECHS1^KR^ mutation, including the suppression of mitochondrial ROS, are accentuated under physiological hypoxia (1% O_2_). In the clinically relevant, heterogeneous tumor microenvironment, where both nutrient flux and oxygen levels are dynamic, we propose that the concurrent operation of these complementary mechanisms drives the pathological elevation of histone crotonylation marks.

Clinically, the association of histone crotonylation and LUAD progression suggests that restricting crotonyl-CoA production through amino acid-limited or fatty acid-restricted diets represents a promising therapeutic strategy, consistent with established nutritional approaches like low-protein diets for cancer patients [46]. While essential/non-essential amino acid restriction inhibits tumor growth in vivo by modulating proliferation, differentiation and immunosurveillance [47–49], specific metabolite-mediated mechanisms in distinct oncogenic contexts remain incompletely characterized. Tryptophan promotes tumor progression via kynurenine-mediated immunosuppression [50, 51], whereas lysine deprivation inhibits growth through AMPK and AKT-AADAT signaling [7, 52], though their effector metabolites are poorly defined. Our findings demonstrate that lysine/tryptophan restriction suppresses lung cancer growth in vitro and in vivo, with mechanistic evidence indicating this effect primarily results from reduced crotonyl-CoA availability rather than impaired protein synthesis, as sodium crotonate supplementation rescues proliferation. This metabolite dependence parallels the Warburg effect, where glycolytic metabolites support tumorigenesis [53]. Crotonyl-CoA promotes oncogenesis in multiple cancers through histone crotonylation [5, 45, 54]. This is exemplified by recent findings that H3K56cr-mediated EGFR-TKI resistance in lung cancer via PI3K-Akt regulation [55]. We further establish that elevated H2BK20cr and H3K27cr levels correlate with poor prognosis in LUAD, consistent with glioblastoma studies showing reprogrammed lysine catabolism sustains growth through crotonyl-CoA-dependent histone modifications [7]. Consequently, restricting lysine/tryptophan to diminish crotonyl-CoA may represent a broad anticancer strategy. Notably, maize, a global staple food naturally deficient in these two amino acids, warrants investigation as a dietary component combined with low-fat regimens to reduce tumorigenic crotonyl-CoA production, although clinical validation is required.

In summary, our study establishes ECHS1 SUMOylation as a critical node linking mitochondrial metabolism to epigenetic regulation. By modulating histone crotonylation, this pathway governs metabolic reprogramming, transcriptional networks, and tumor growth. These insights advance our understanding of nutrient-sensing mechanisms and provide potential targets for metabolic intervention in lung cancer.

## Supplementary information

Supplementary information

Supplementary Figure S1

Supplementary Figure S2

Supplementary Figure S3

Supplementary Figure S4

Supplementary Figure S5

Supplementary Figure S6

Supplementary Table S1

Supplementary Table S2

Supplementary Table S3

Uncropped Western blot

Reproducibility checklist

## Data Availability

Uncropped Western blot images are provided in Supplementary Fig. S6. All data are available in the manuscript or the supplementary materials. Omics data are accessible in NODE (https://www.biosino.org/node/) with the accession number OEP00006978 or through the URL: https://www.biosino.org/node/project/detail/OEP00006978. All unique and stable reagents generated in this study are available from the lead contacts on reasonable request with a completed materials transfer agreement.
