# Supplementary material for: SUMOylation activates ECHS1 for adaptive catabolism in lung cancer
Source: Cell Death Dis. 2026 Jul 9;17(1):854. doi: 10.1038/s41419-026-09078-1 (PMC13631240; doi:10.1038/s41419-026-09078-1)
Supplement: Supplementary file 1 [file 41419_2026_9078_MOESM11_ESM.pdf]

Figure 1A

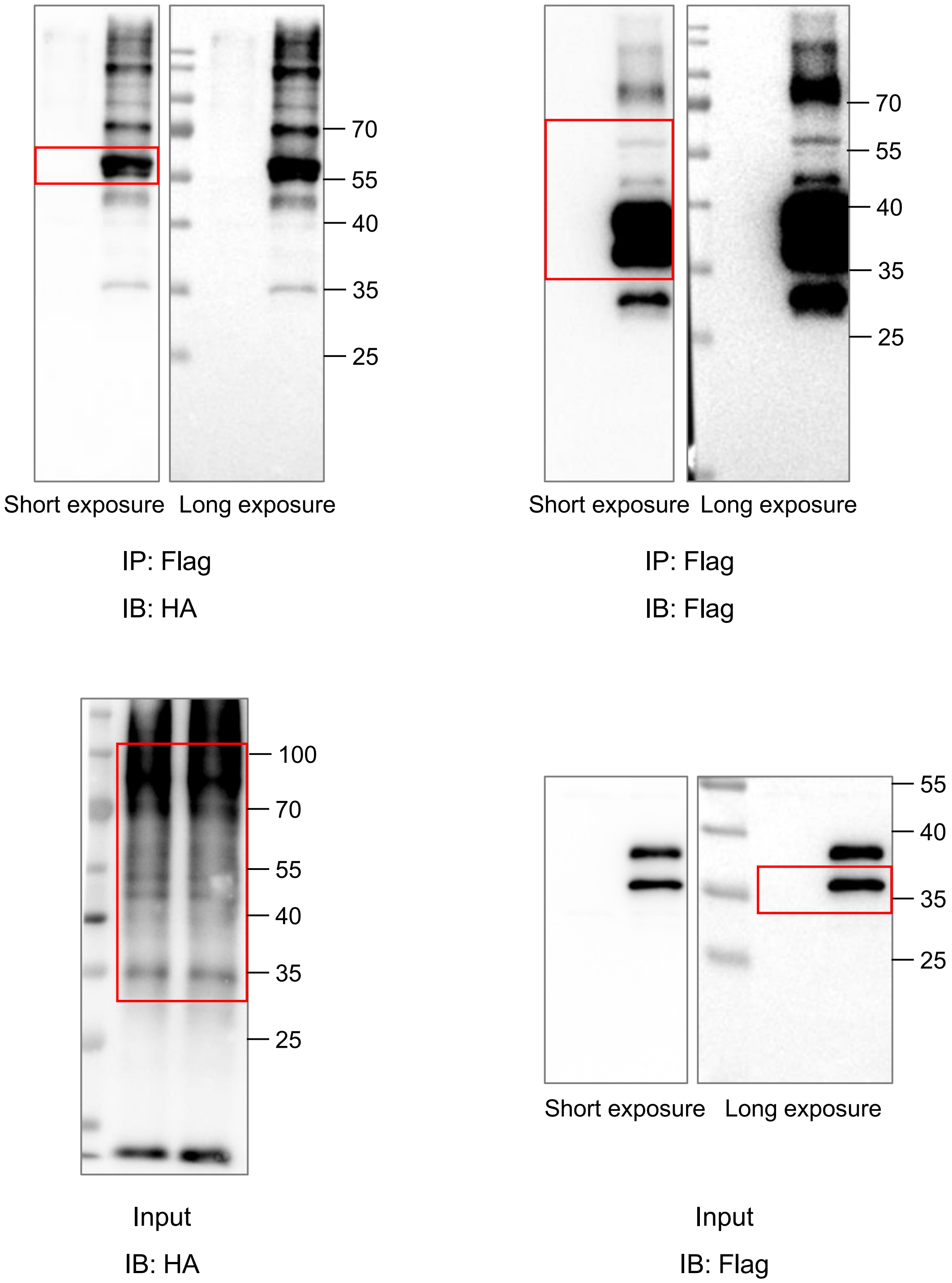
70
55
40
35
25
Short exposure
Long exposure
IP: Flag
IB: HA
70
55
40
35
25
Short exposure
Long exposure
IP: Flag
IB: Flag
100
70
55
40
35
25
Input
IB: HA
55
40
35
25
Short exposure
Long exposure
Input
IB: Flag

Figure 1B

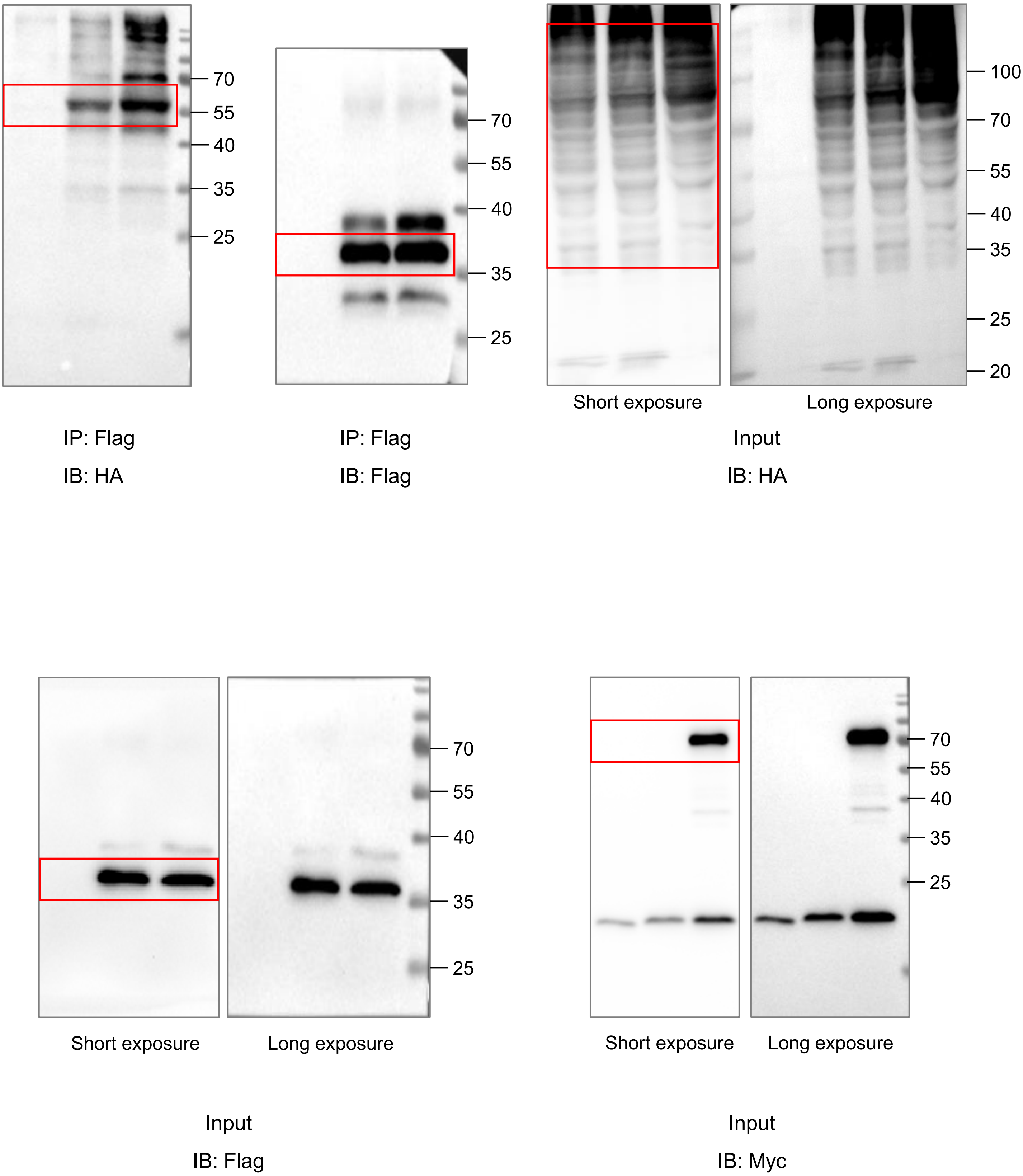
70
55
40
35
25
IP: Flag
IB: HA
70
55
40
35
25
IP: Flag
IB: Flag
100
70
55
40
35
25
20
Short exposure
Long exposure
Input
IB: HA
70
55
40
35
25
Short exposure
Long exposure
Input
IB: Flag
70
55
40
35
25
Short exposure
Long exposure
Input
IB: Myc

Figure 1C

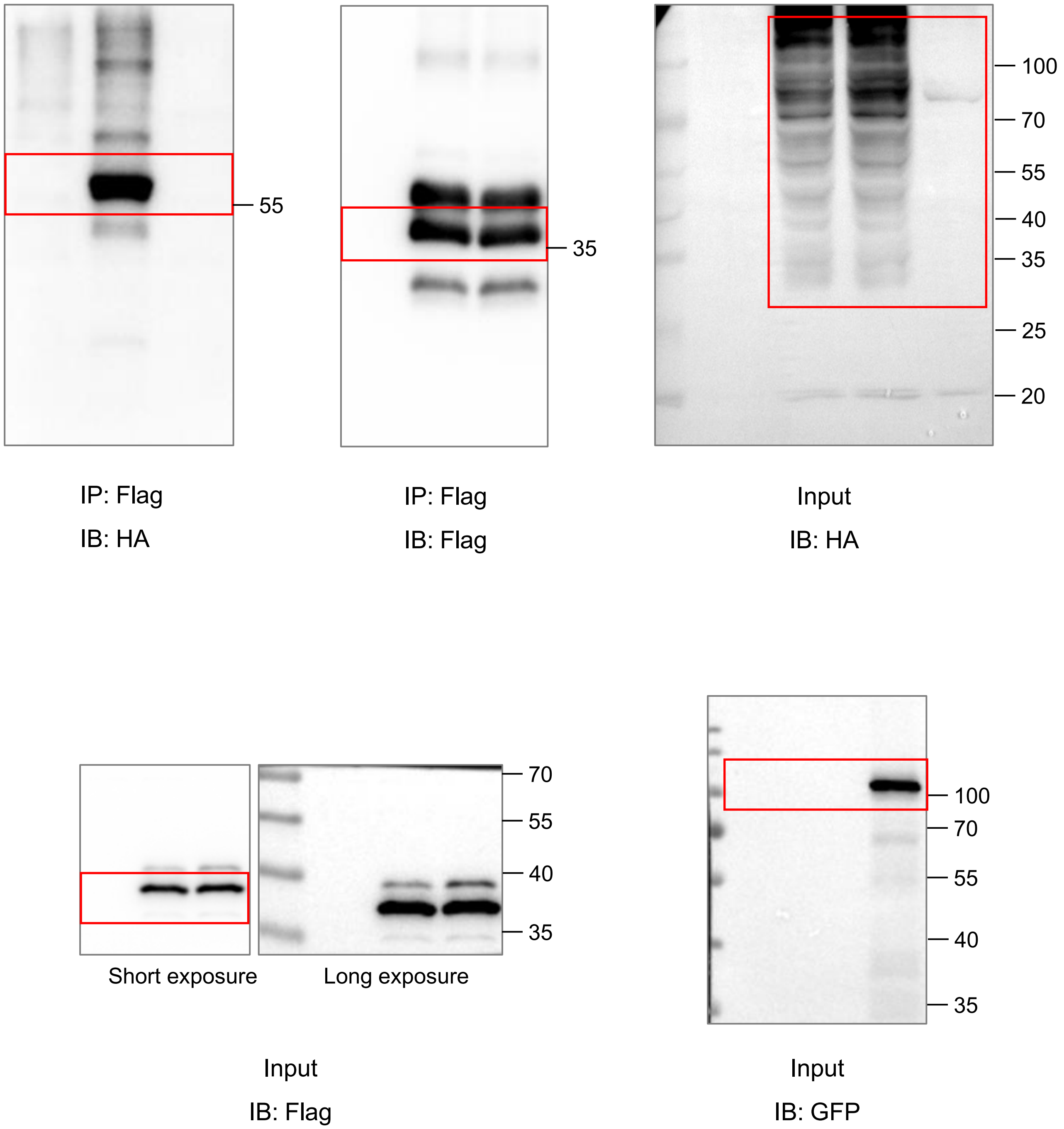
55
35
100
70
55
40
35
25
20
IP: Flag
IB: HA
IP: Flag
IB: Flag
Input
IB: HA
70
55
40
35
Short exposure
Long exposure
100
70
55
40
35
Input
IB: Flag
Input
IB: GFP

Figure 1D

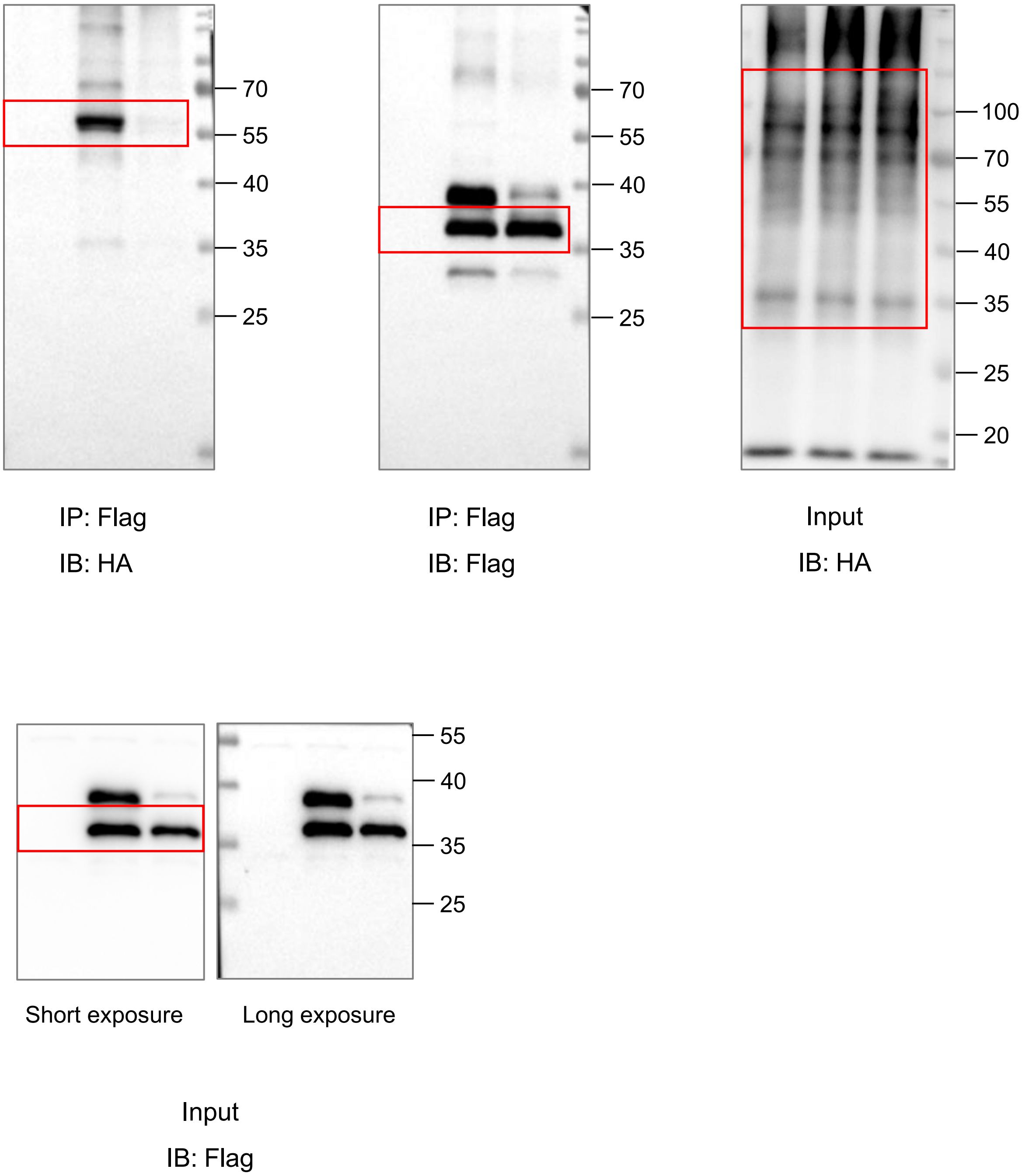

70
55
40
35
25
IP: Flag
IB: HA
70
55
40
35
25
IP: Flag
IB: Flag
100
70
55
40
35
25
20
Input
IB: HA
55
40
35
25
Short exposure
Long exposure
Input
IB: Flag

Figure 1E

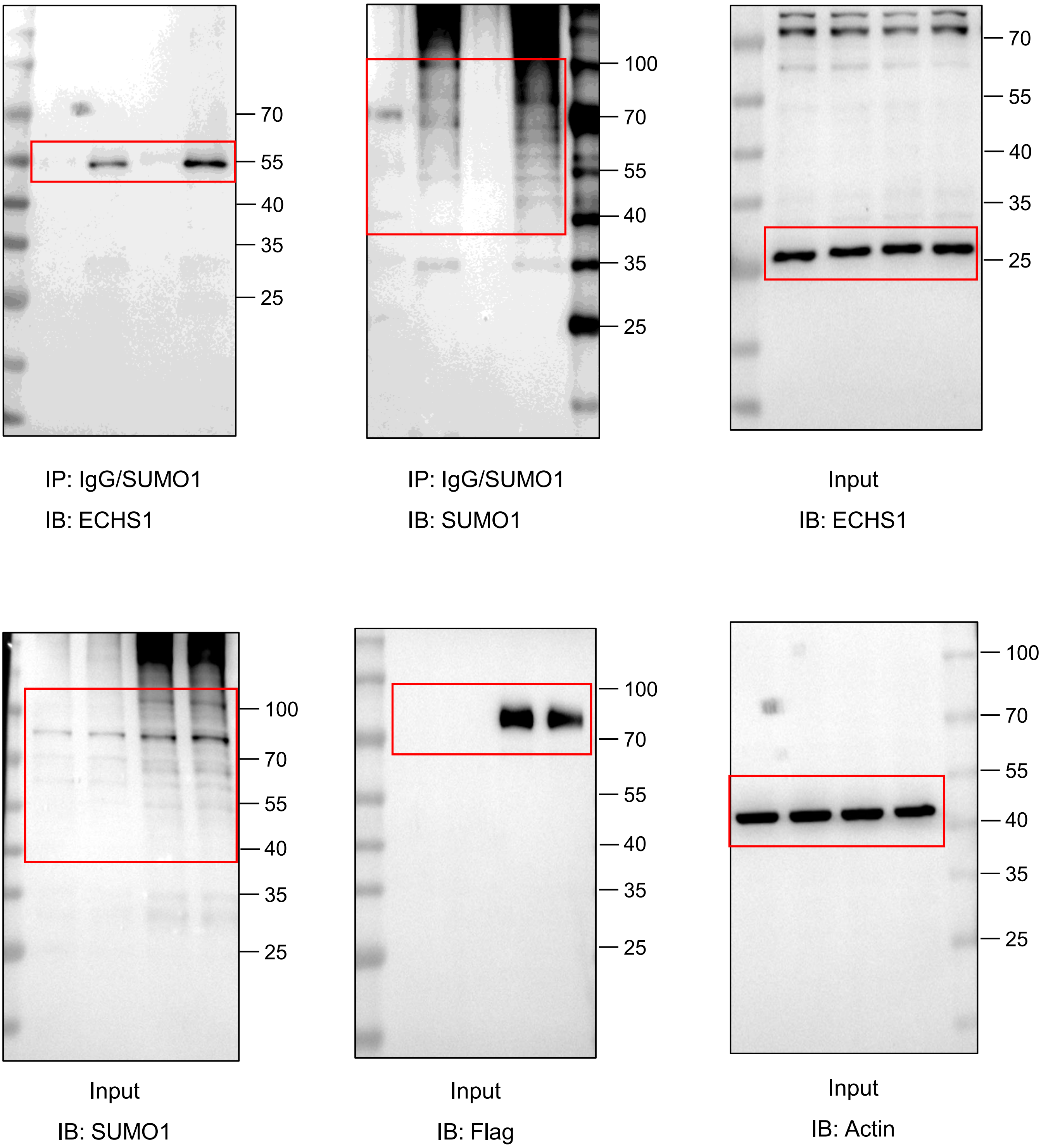
70
55
40
35
25
IP: IgG/SUMO1
IB: ECHS1
100
70
55
40
35
25
IP: IgG/SUMO1
IB: SUMO1
70
55
40
35
25
Input
IB: ECHS1
100
70
55
40
35
25
Input
IB: SUMO1
100
70
55
40
35
25
Input
IB: Flag
100
70
55
40
35
25
Input
IB: Actin

Figure 1G

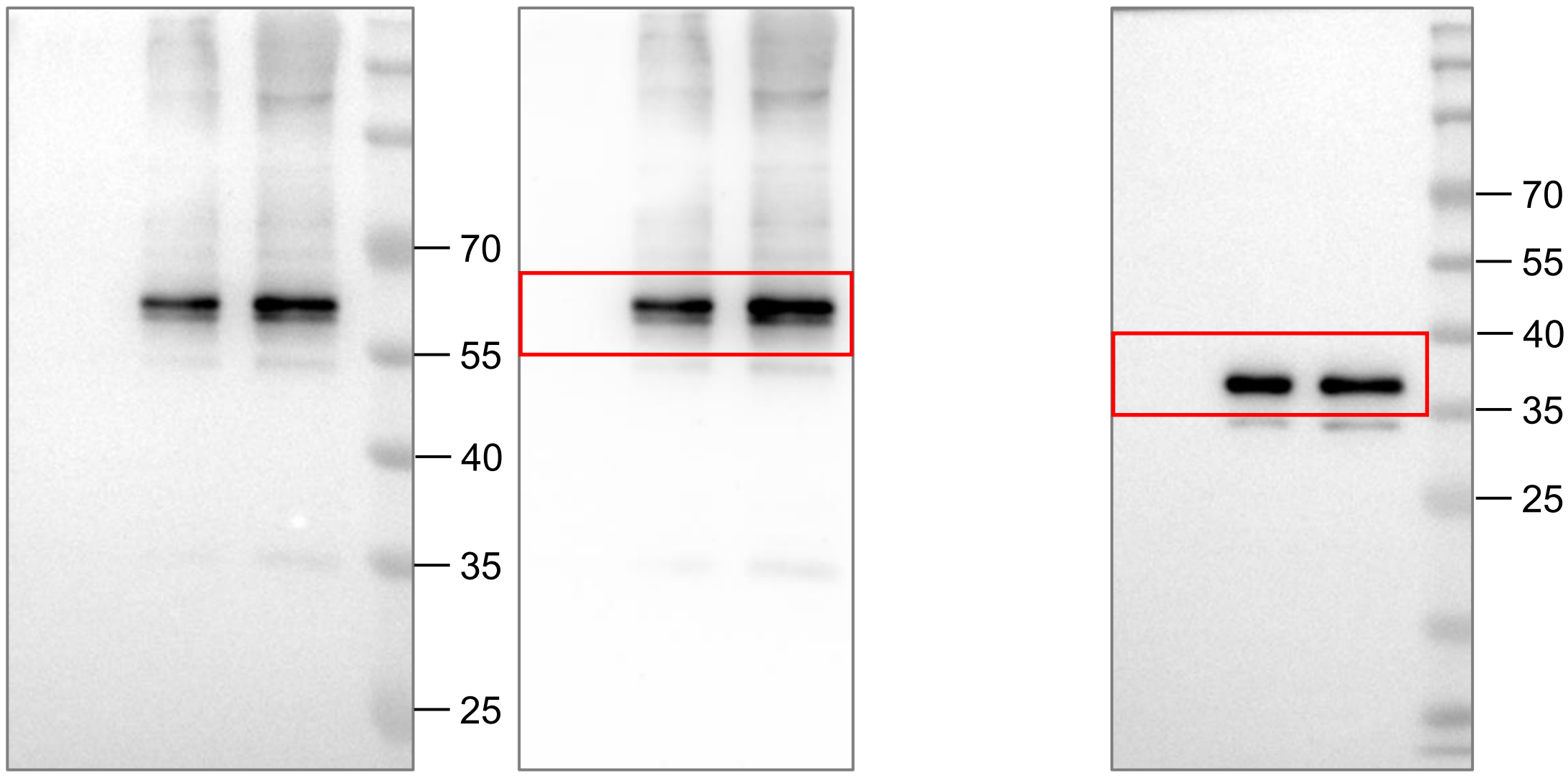

Short exposure

Long exposure

IP: Flag

IB: HA

IP: Flag

IB: Flag

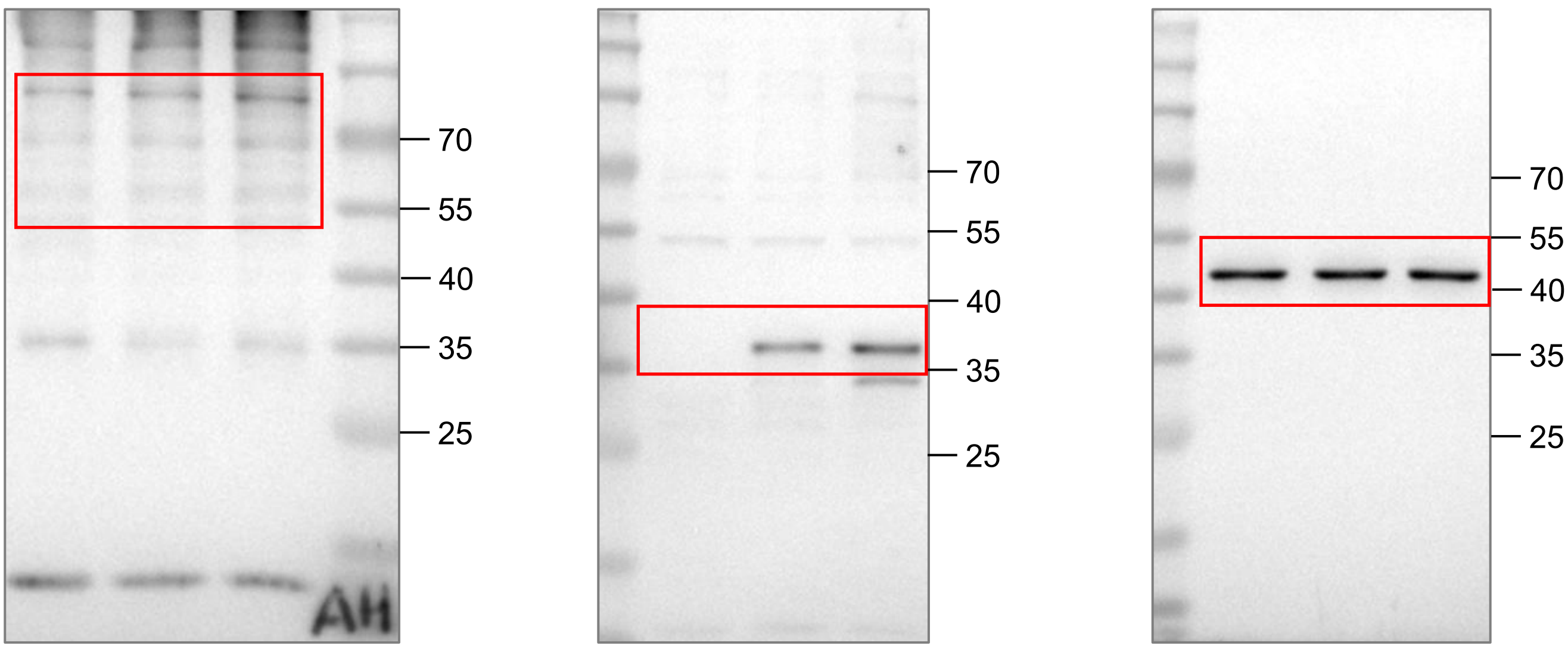

Input

IB: HA

Input

IB: Flag

Input

IB: Actin

Figure 1H

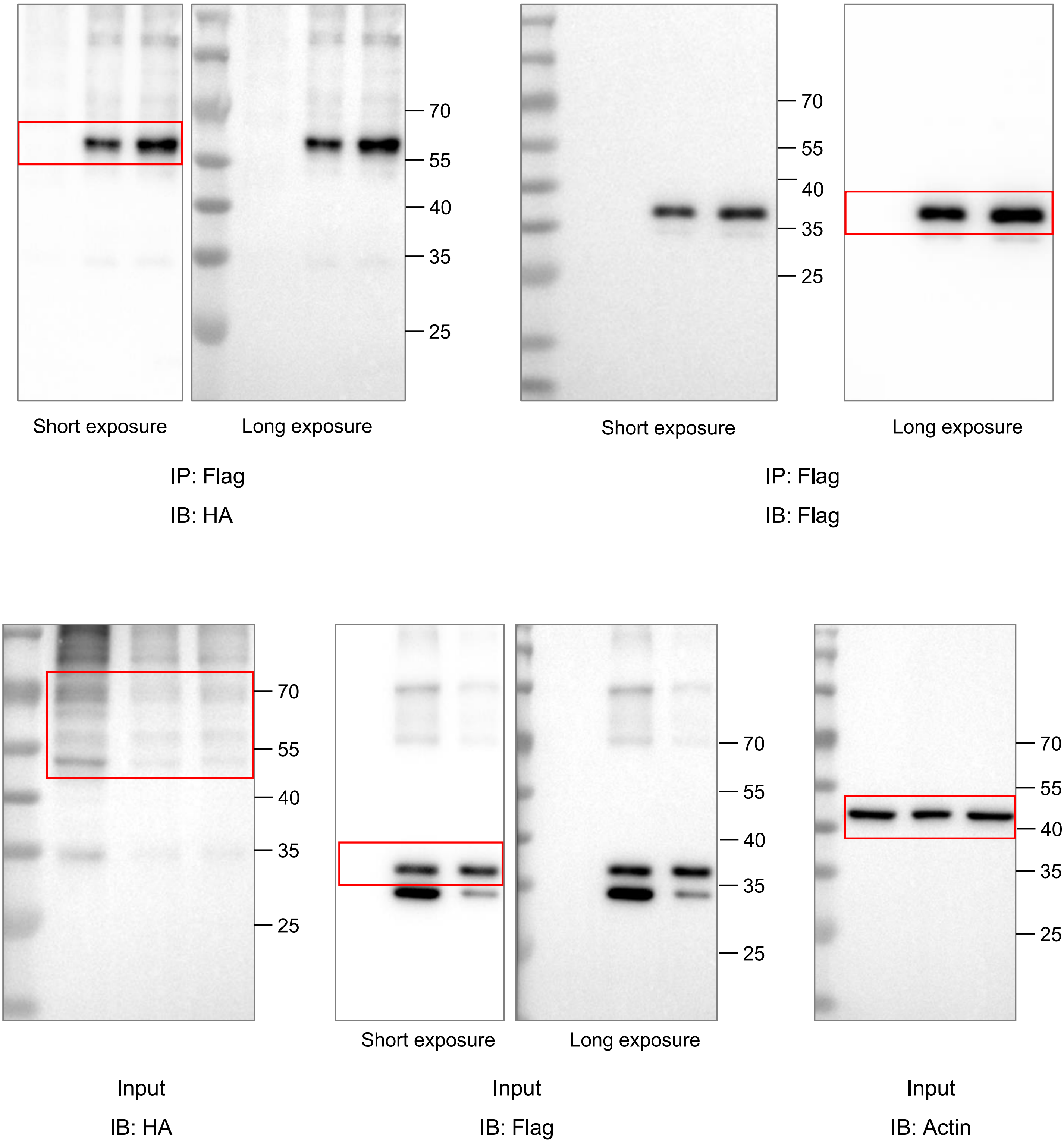
70
55
40
35
25
Short exposure
Long exposure
IP: Flag
IB: HA
70
55
40
35
25
Short exposure
Long exposure
IP: Flag
IB: Flag
70
55
40
35
25
Input
IB: HA
70
55
40
35
25
Short exposure
Long exposure
Input
IB: Flag
70
55
40
35
25
Input
IB: Actin

Figure 1I

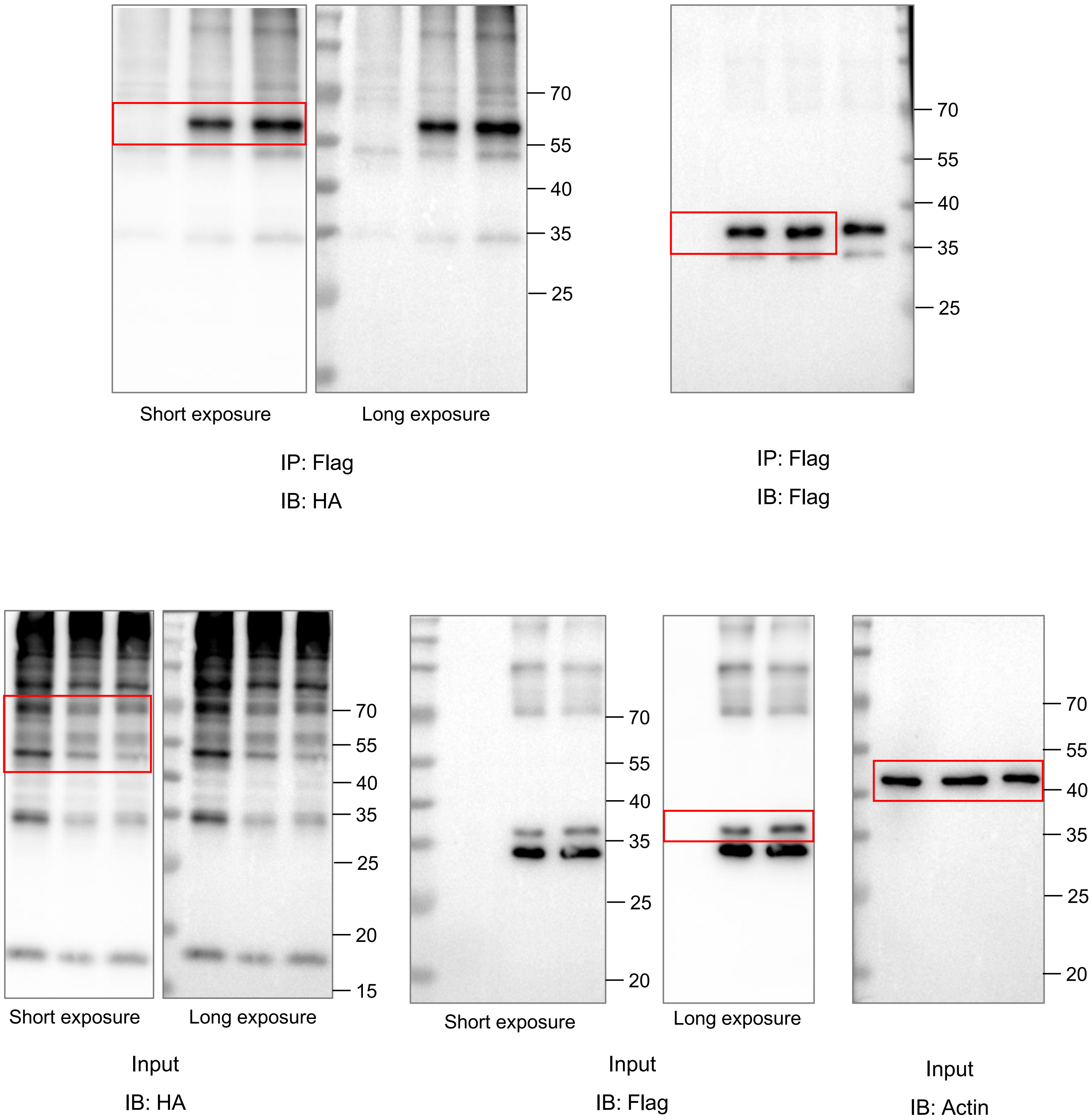
70
55
40
35
25
Short exposure
Long exposure
IP: Flag
IB: HA
70
55
40
35
25
IP: Flag
IB: Flag
70
55
40
35
25
20
15
Short exposure
Long exposure
Input
IB: HA
70
55
40
35
25
20
Short exposure
Long exposure
Input
IB: Flag
70
55
40
35
25
20
Input
IB: Actin

Figure 1J

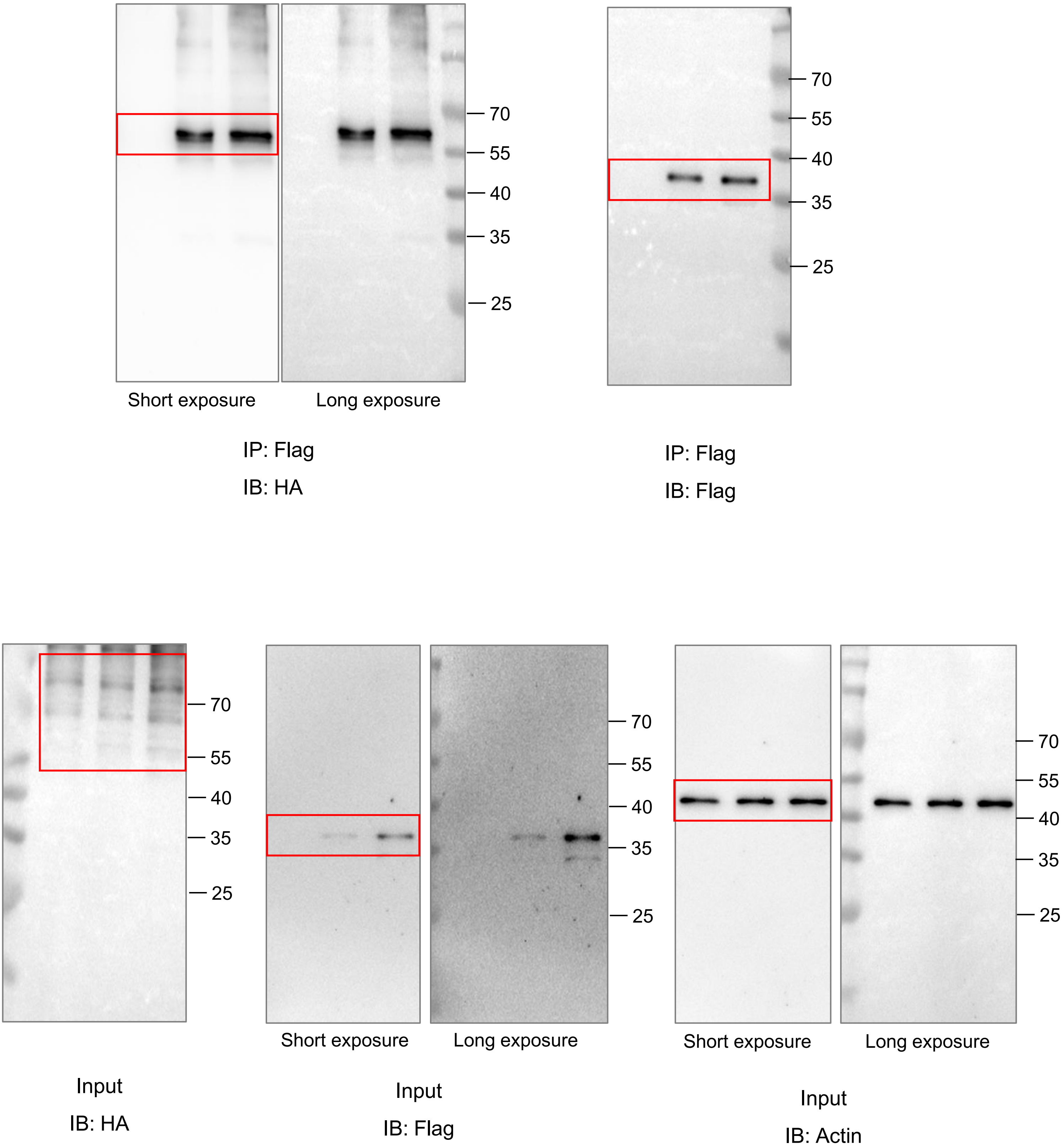
70
55
40
35
25
Short exposure
Long exposure
IP: Flag
IB: HA
70
55
40
35
25
IP: Flag
IB: Flag
70
55
40
35
25
Input
IB: HA
70
55
40
35
25
Short exposure
Long exposure
Input
IB: Flag
70
55
40
35
25
Short exposure
Long exposure
Input
IB: Actin

Figure 2F

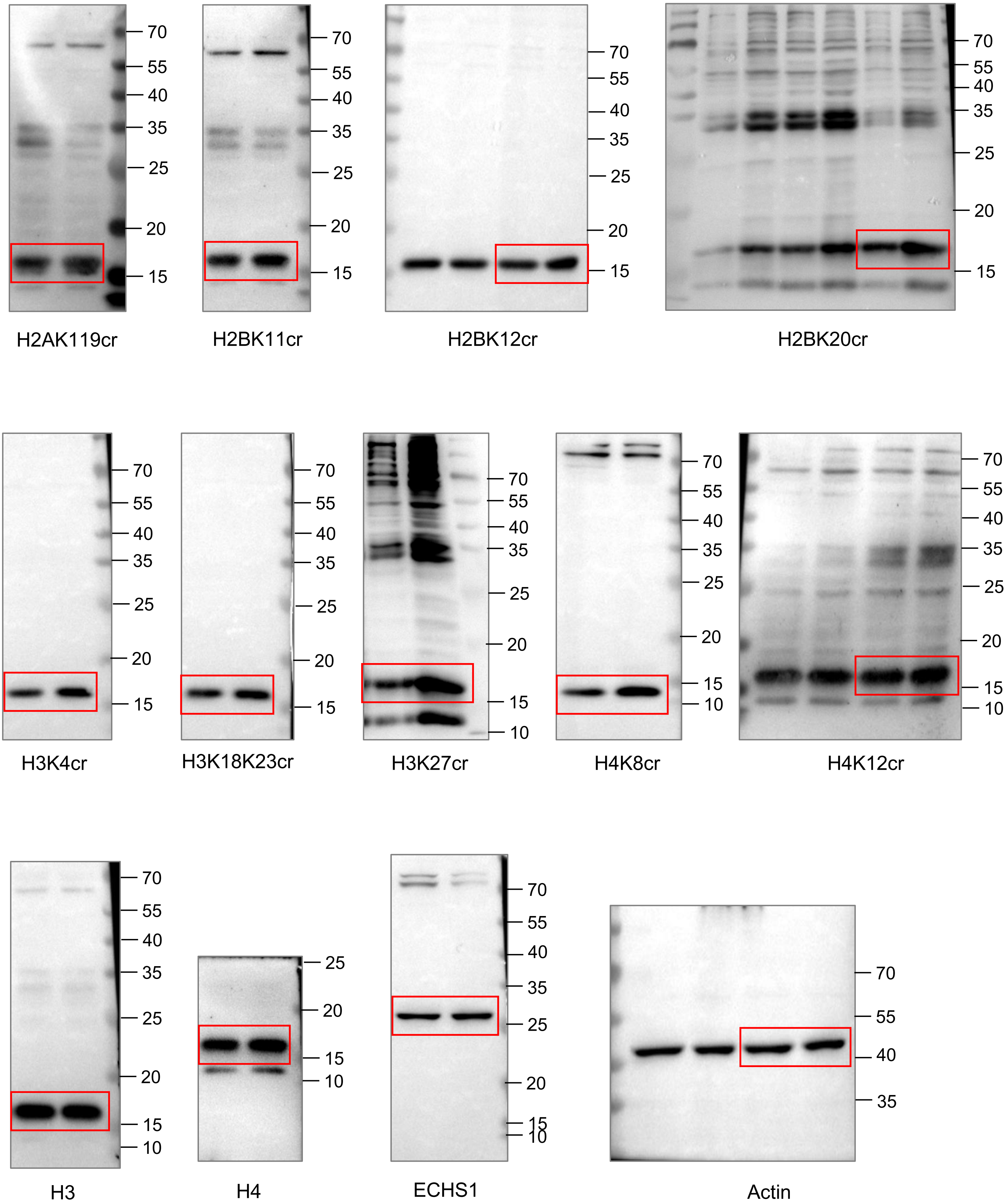

H2AK119cr
H2BK11cr
H2BK12cr
H2BK20cr
H3K4cr
H3K18K23cr
H3K27cr
H4K8cr
H4K12cr
H3
H4
ECHS1
Actin

Figure 2J

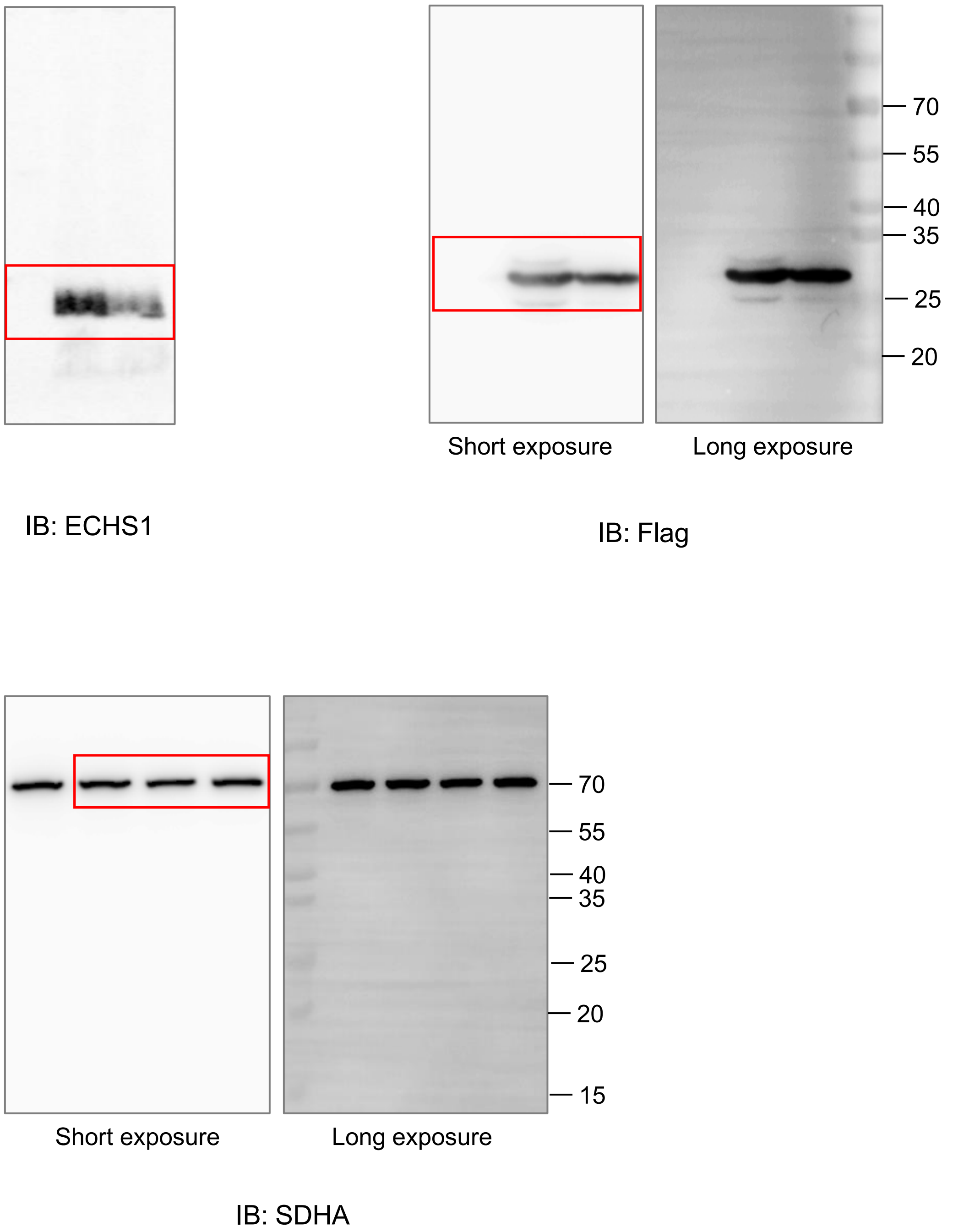
70
55
40
35
25
20
Short exposure
Long exposure
IB: ECHS1
IB: Flag
70
55
40
35
25
20
15
Short exposure
Long exposure
IB: SDHA

Figure 2K

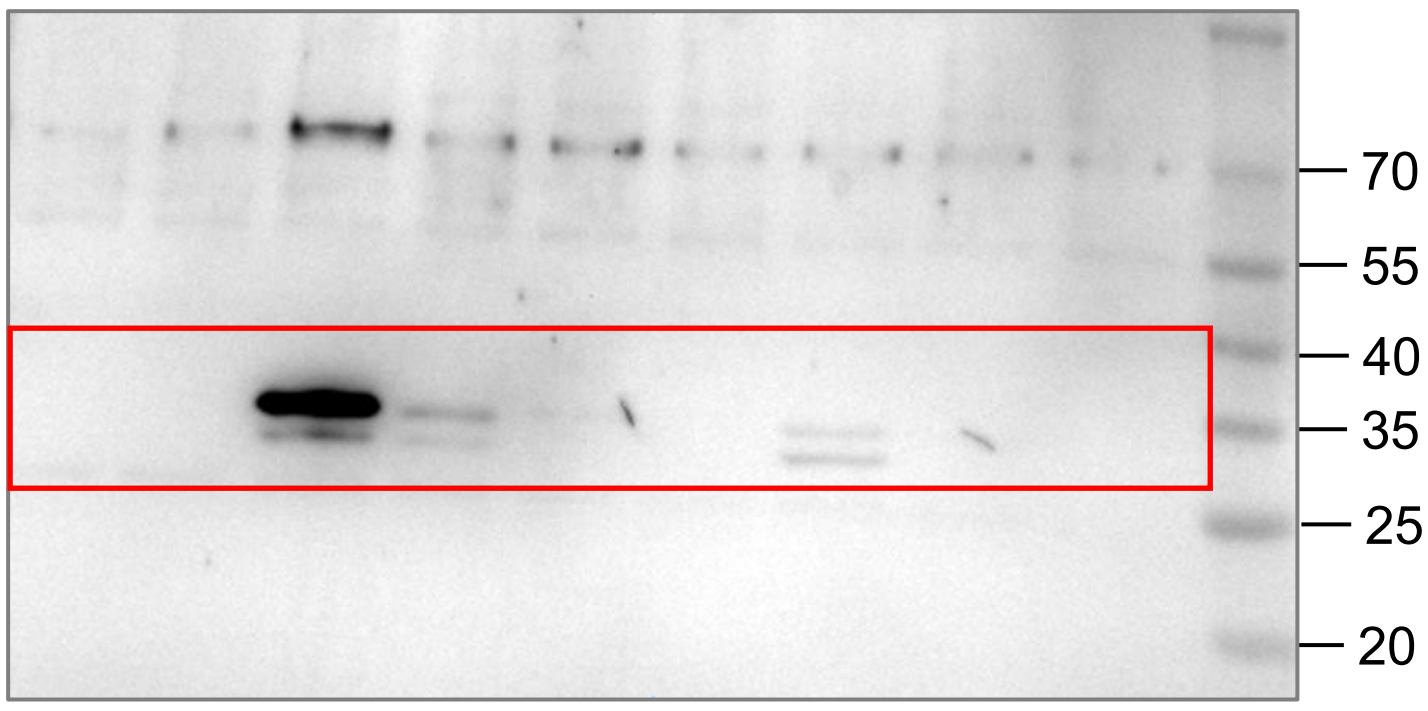

IP: Myc
IB: Flag

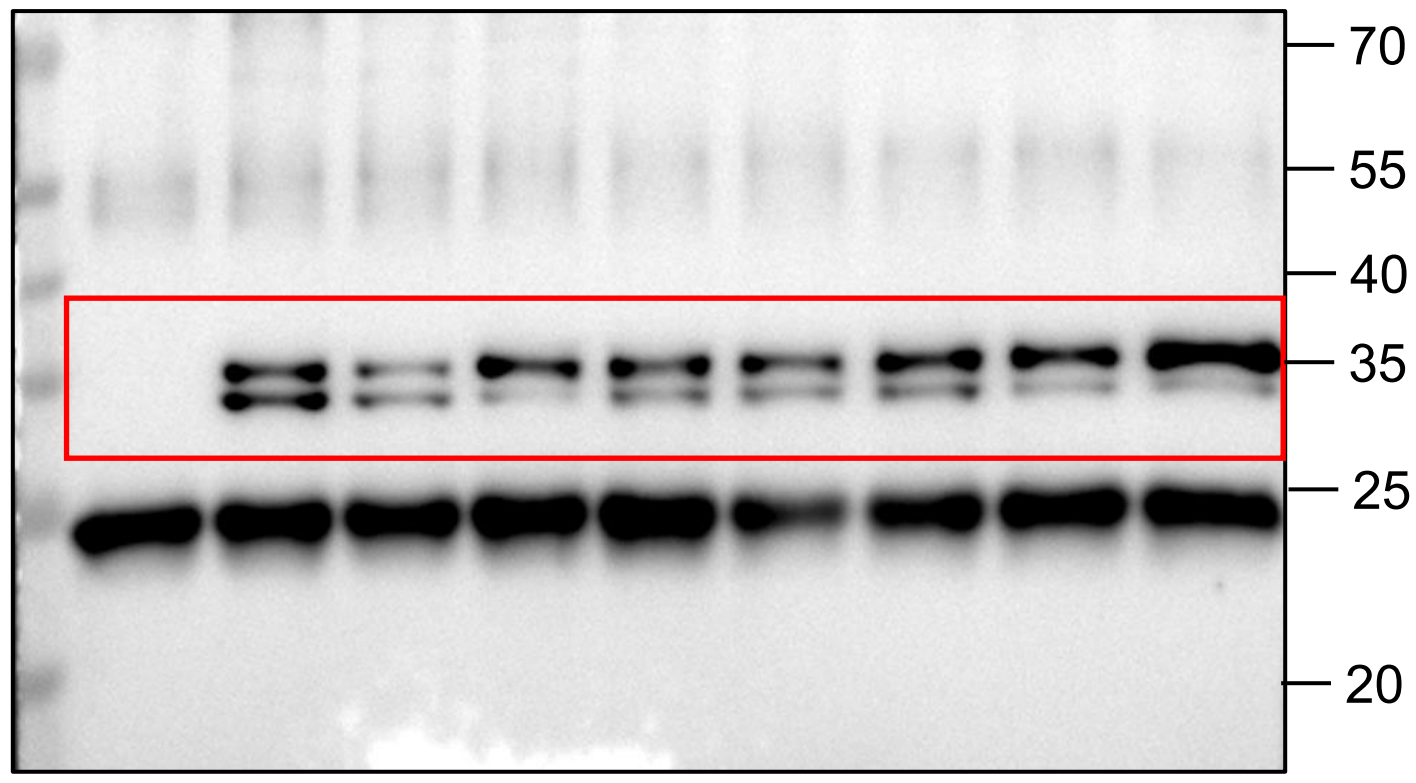

IP: Myc
IB: Myc

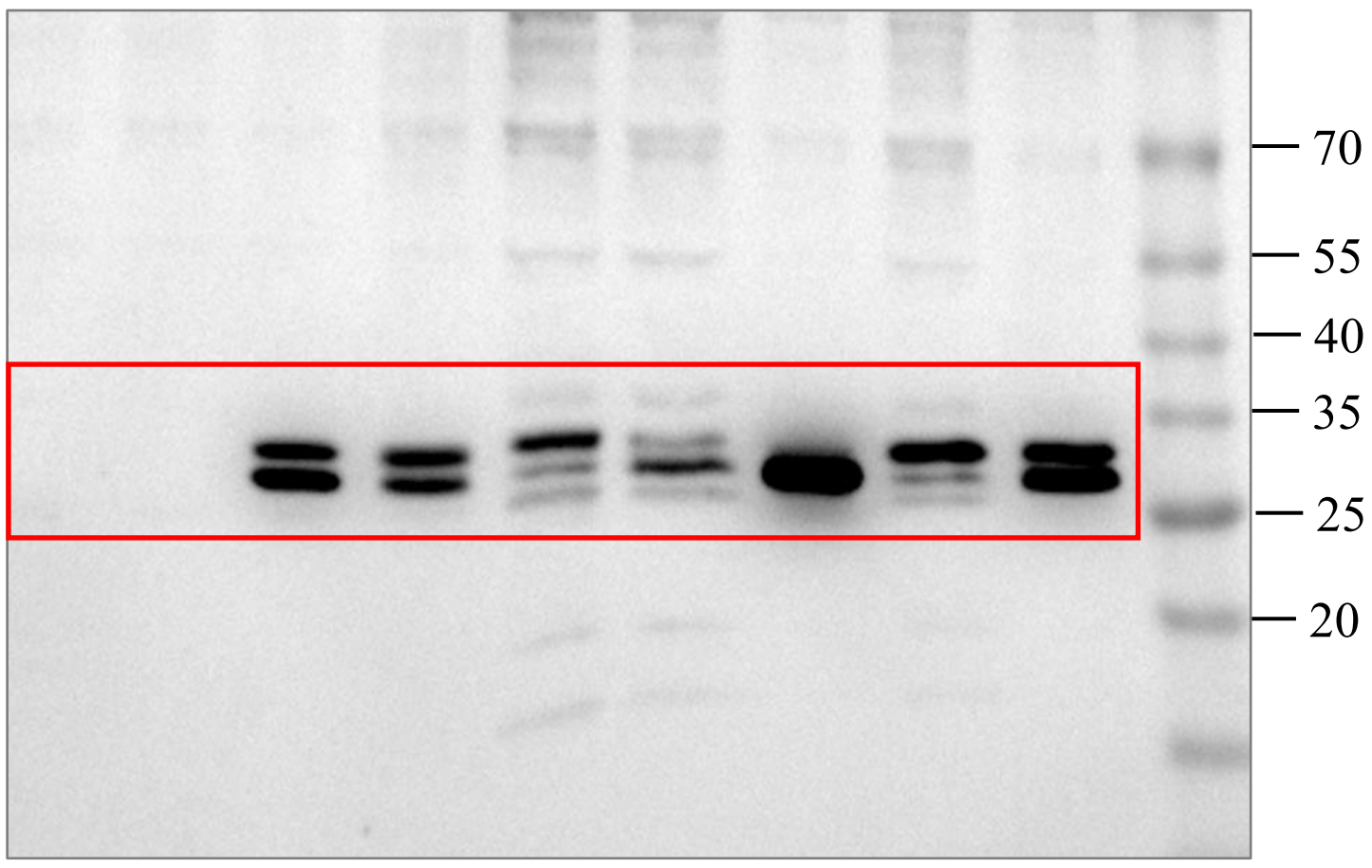

Input
IB: Flag

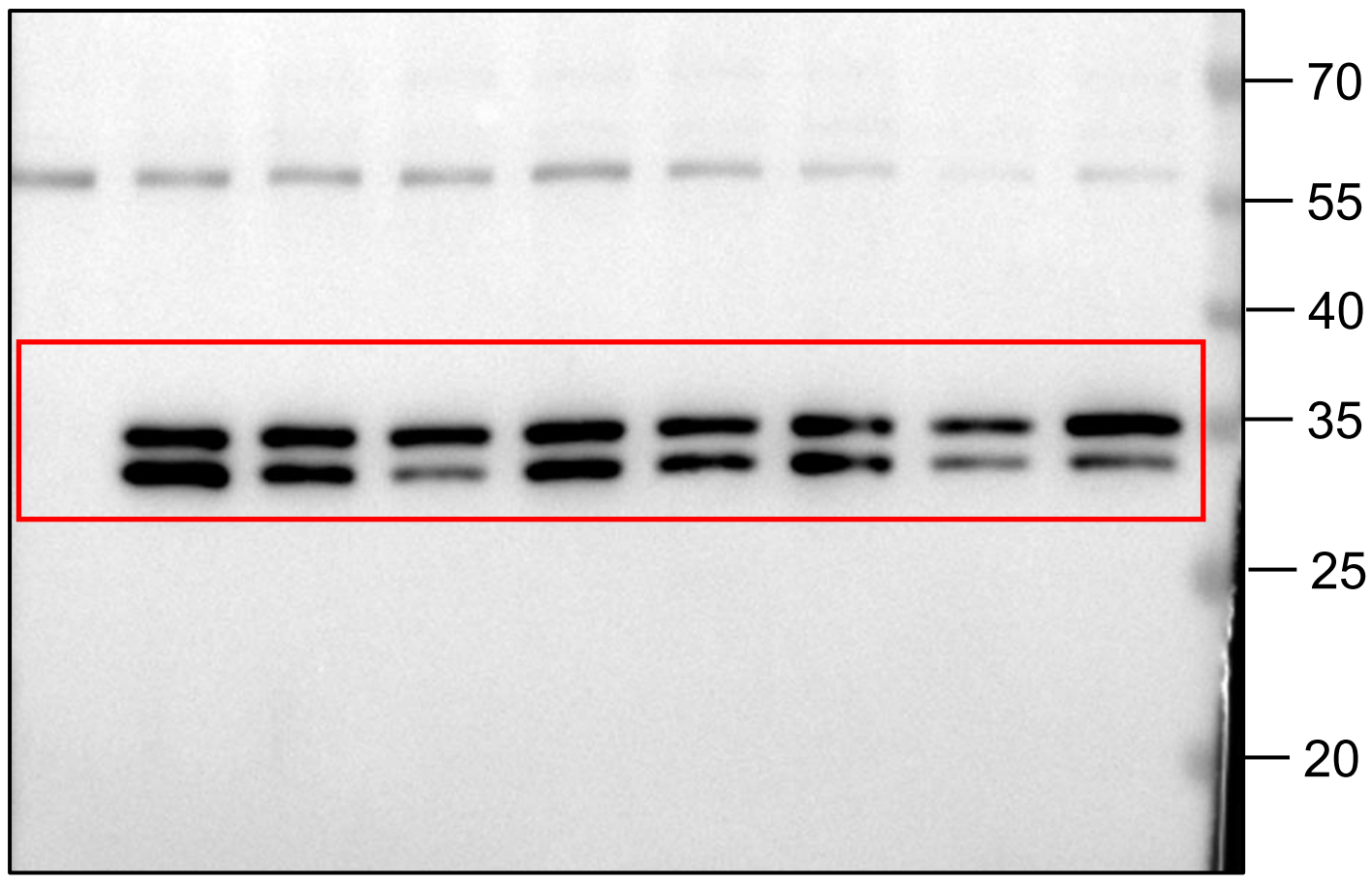

Input
IB: Myc

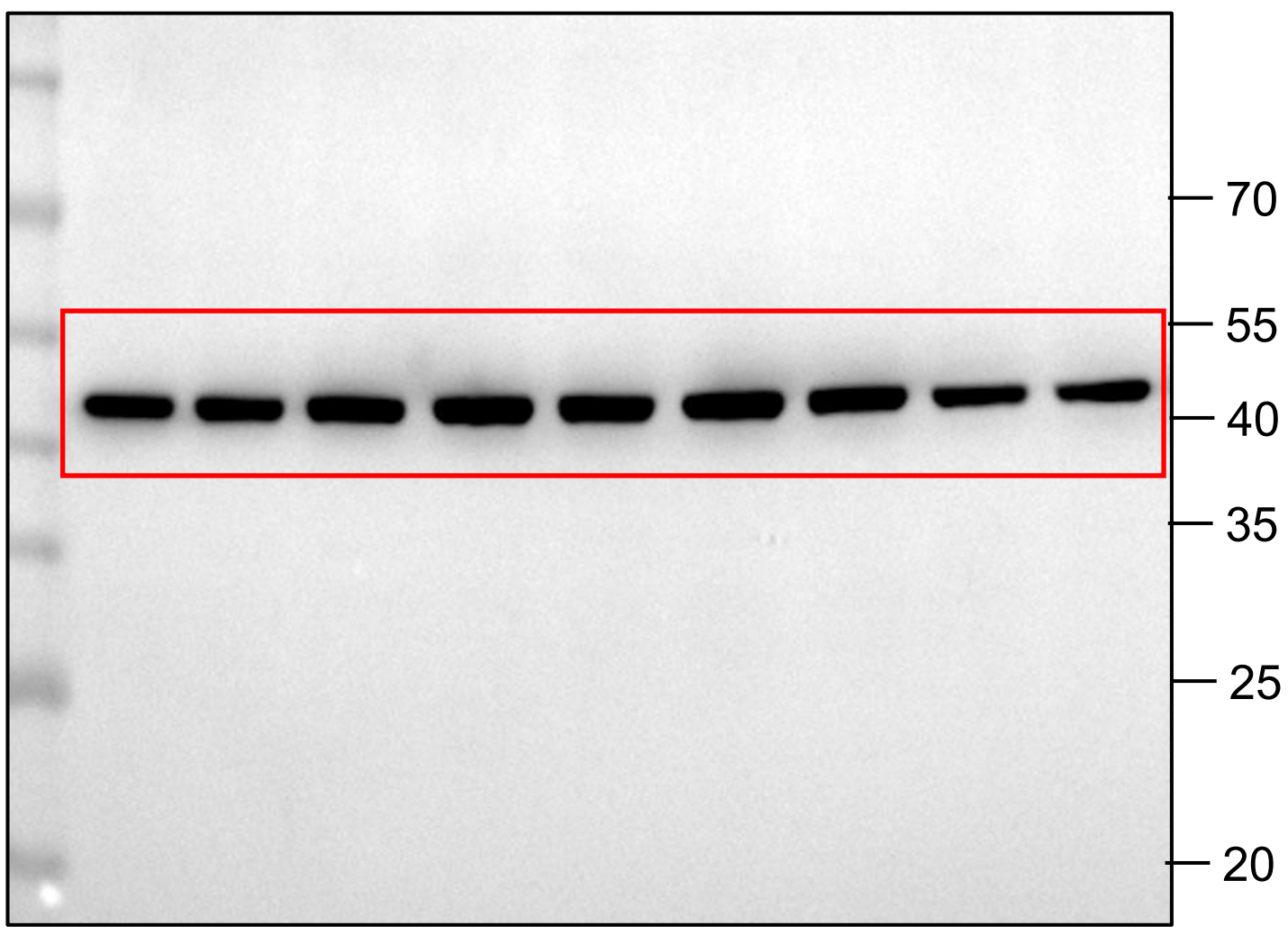

Input
IB: Actin

Figure 3E

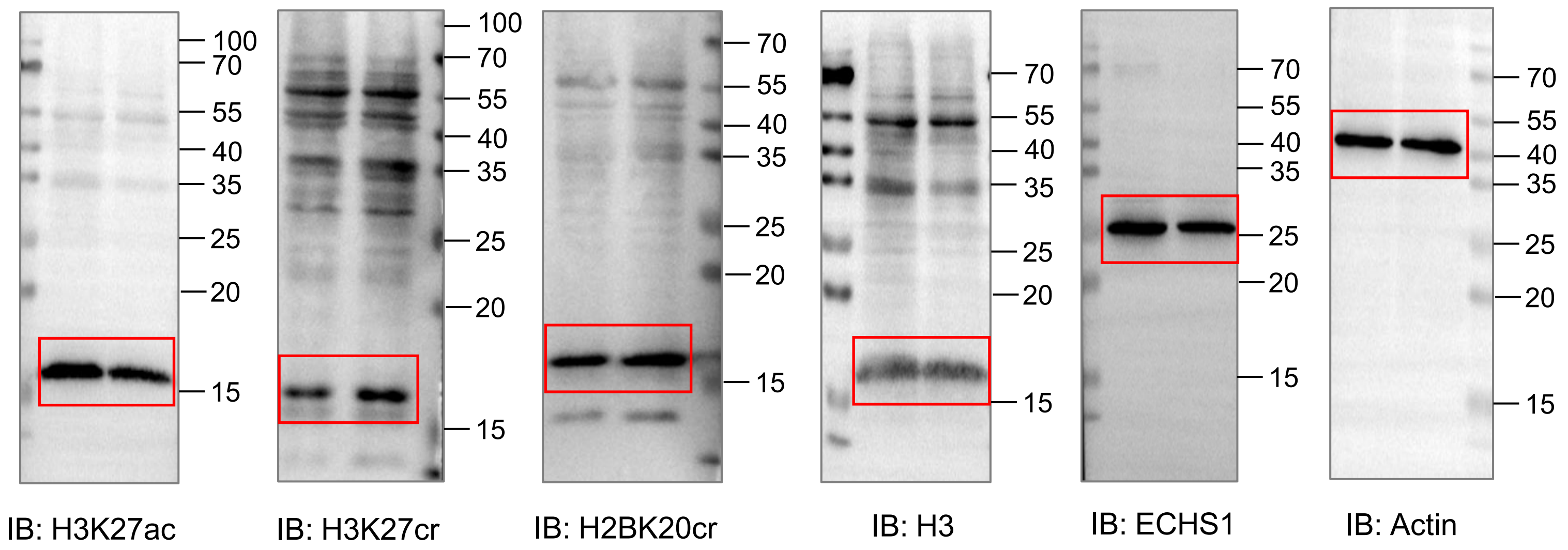

100
70
55
40
35
25
20
15
IB: H3K27ac
100
70
55
40
35
25
20
15
IB: H3K27cr
70
55
40
35
25
20
15
IB: H2BK20cr
70
55
40
35
25
20
15
IB: H3
70
55
40
35
25
20
15
IB: ECHS1
70
55
40
35
25
20
15
IB: Actin

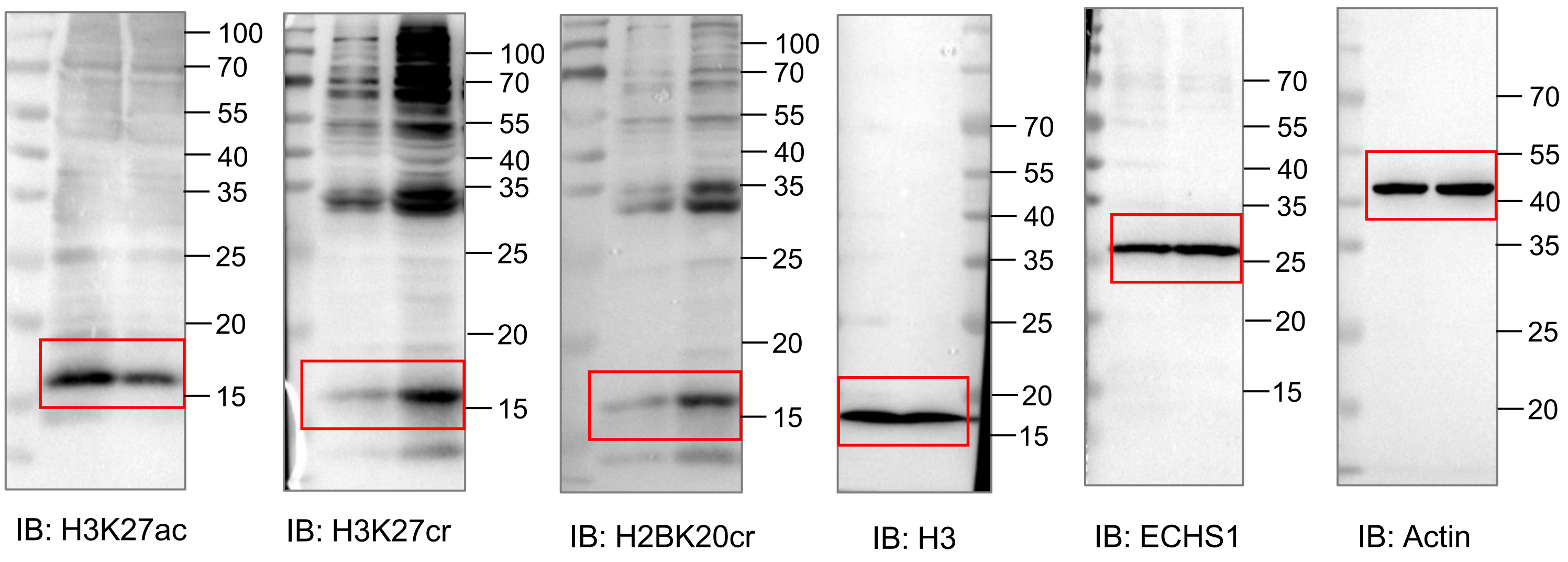

100
70
55
40
35
25
20
15
IB: H3K27ac
100
70
55
40
35
25
20
15
IB: H3K27cr
100
70
55
40
35
25
20
15
IB: H2BK20cr
70
55
40
35
25
20
15
IB: H3
70
55
40
35
25
20
15
IB: ECHS1
70
55
40
35
25
20
IB: Actin

Figure 4I

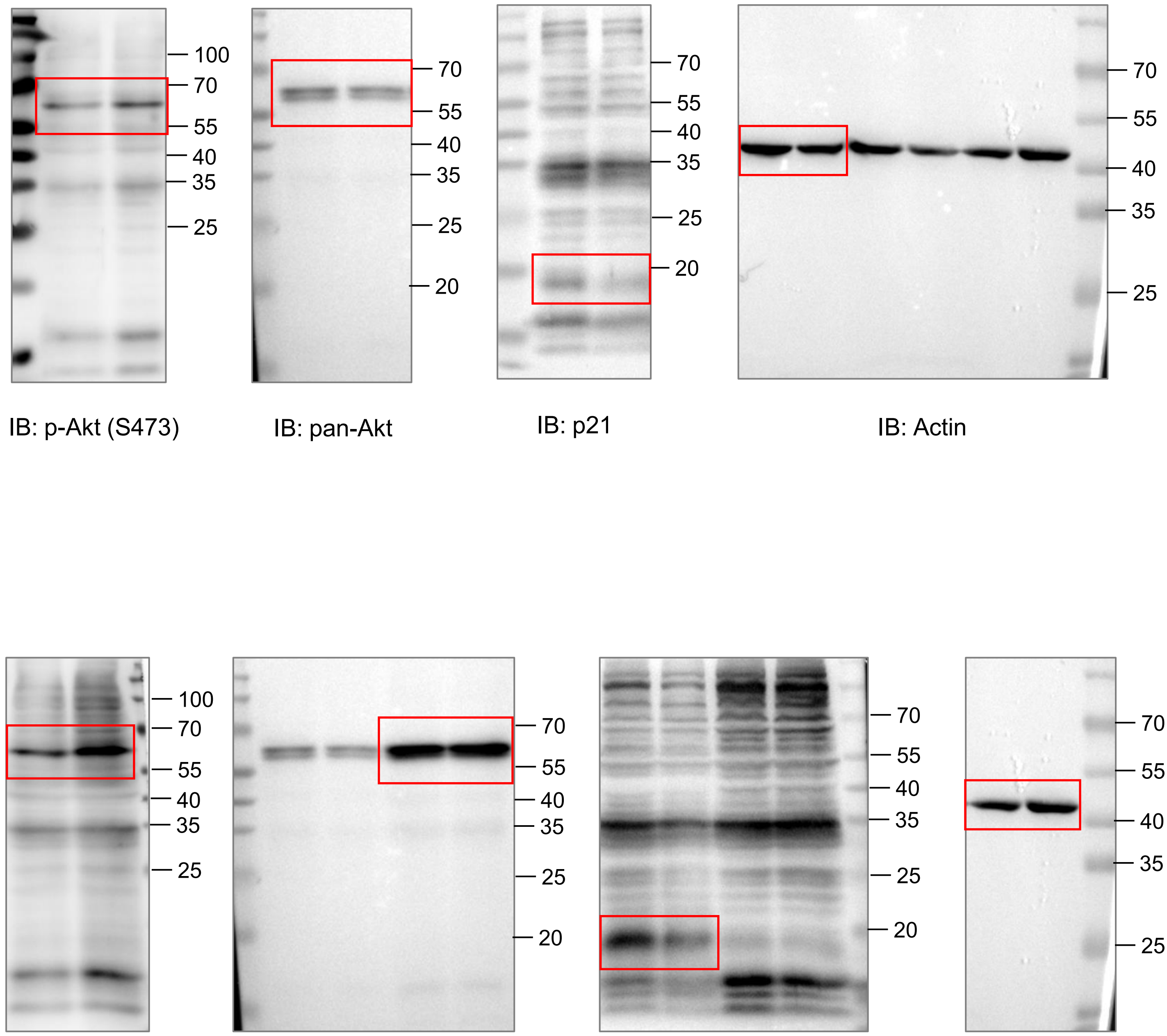

IB: p-Akt (S473) IB: pan-Akt IB: p21 IB: Actin

IB: p-Akt (S473) IB: pan-Akt IB: p21 IB: Actin

Figure 5G

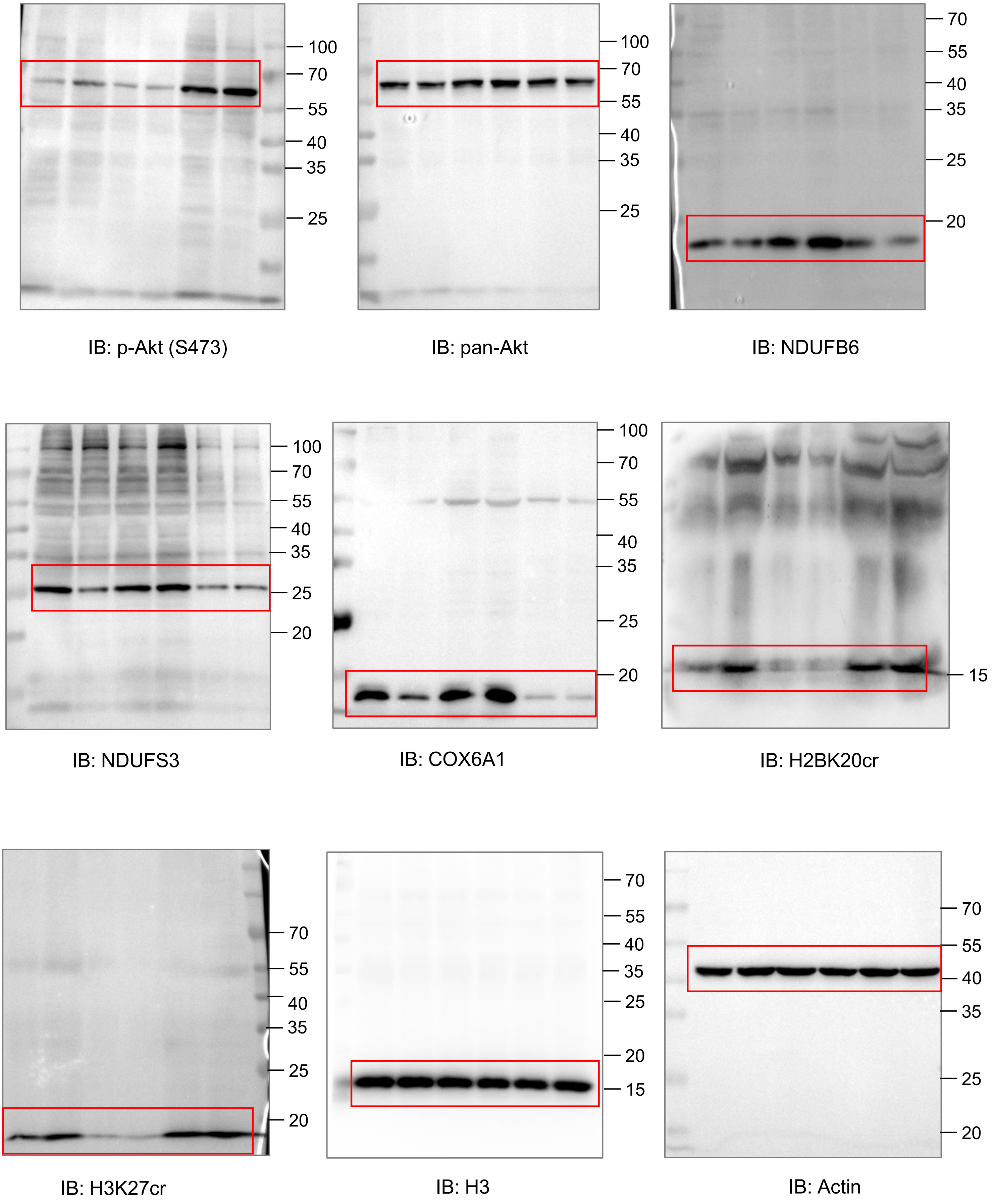

100
70
55
40
35
25
IB: p-Akt (S473)
100
70
55
40
35
25
IB: pan-Akt
70
55
40
35
25
20
IB: NDUFB6
100
70
55
40
35
25
20
IB: NDUFS3
100
70
55
40
35
25
20
IB: COX6A1
15
IB: H2BK20cr
70
55
40
35
25
20
IB: H3K27cr
70
55
40
35
25
20
15
IB: H3
70
55
40
35
25
20
IB: Actin

Supplementary Figure S2A

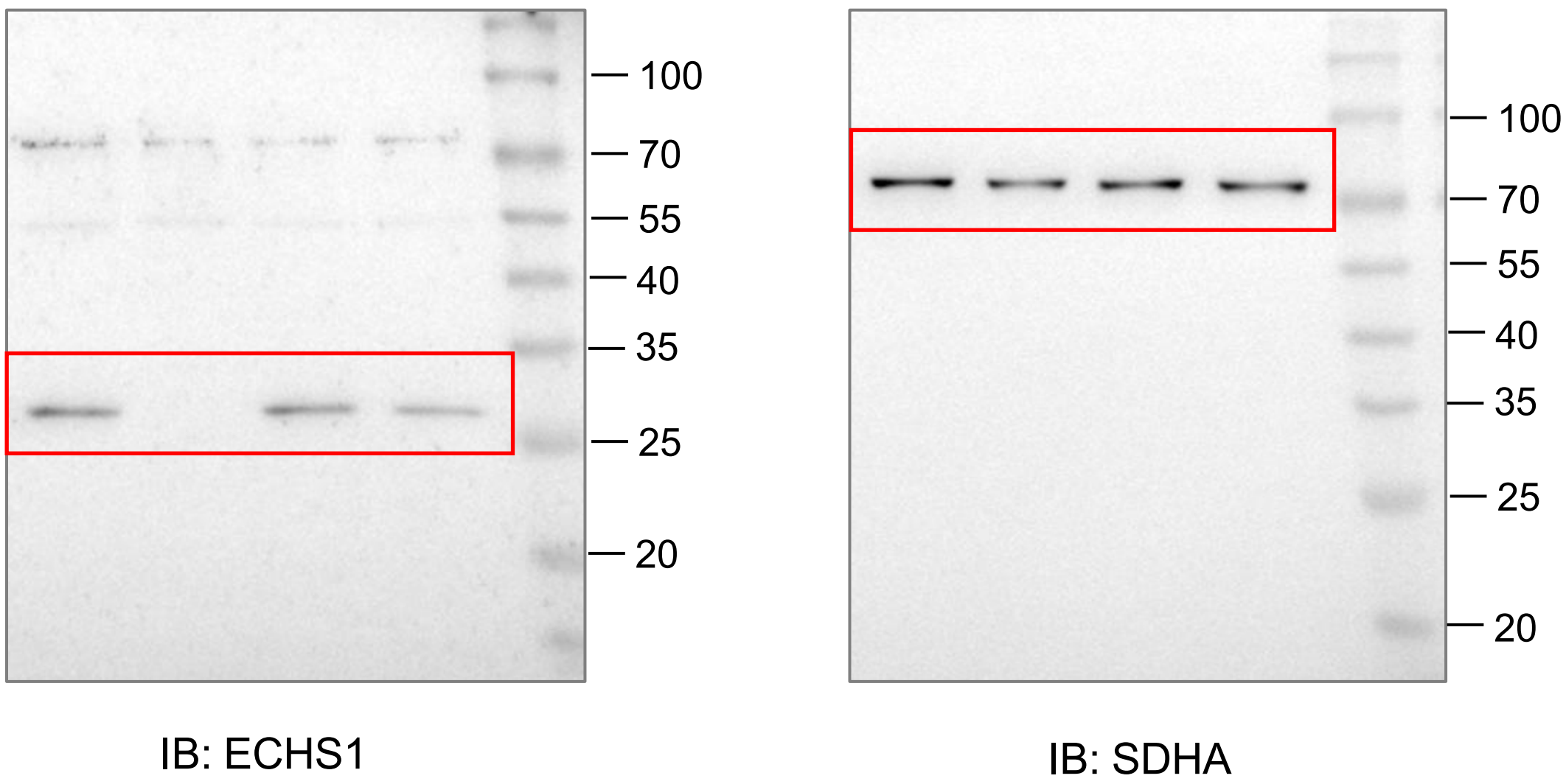

Supplementary Figure S2C

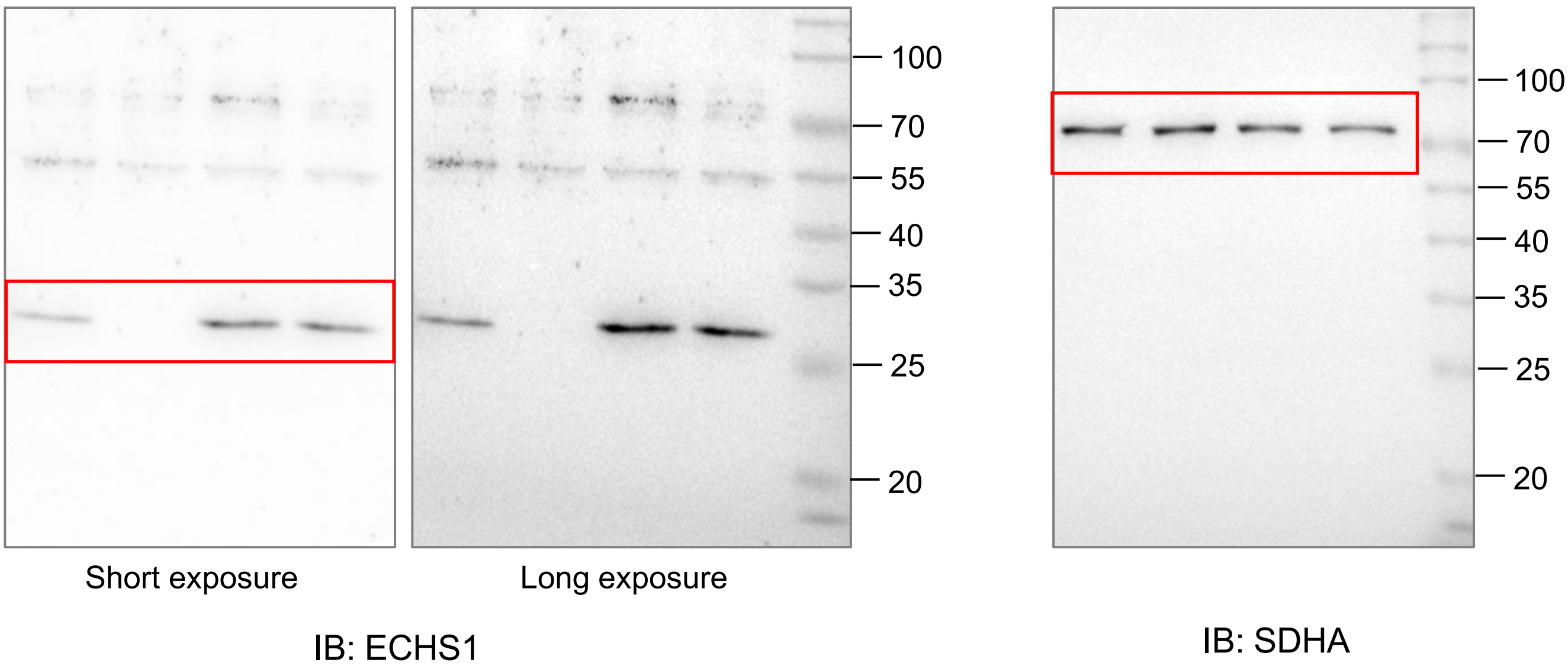

Supplementary Figure S2E

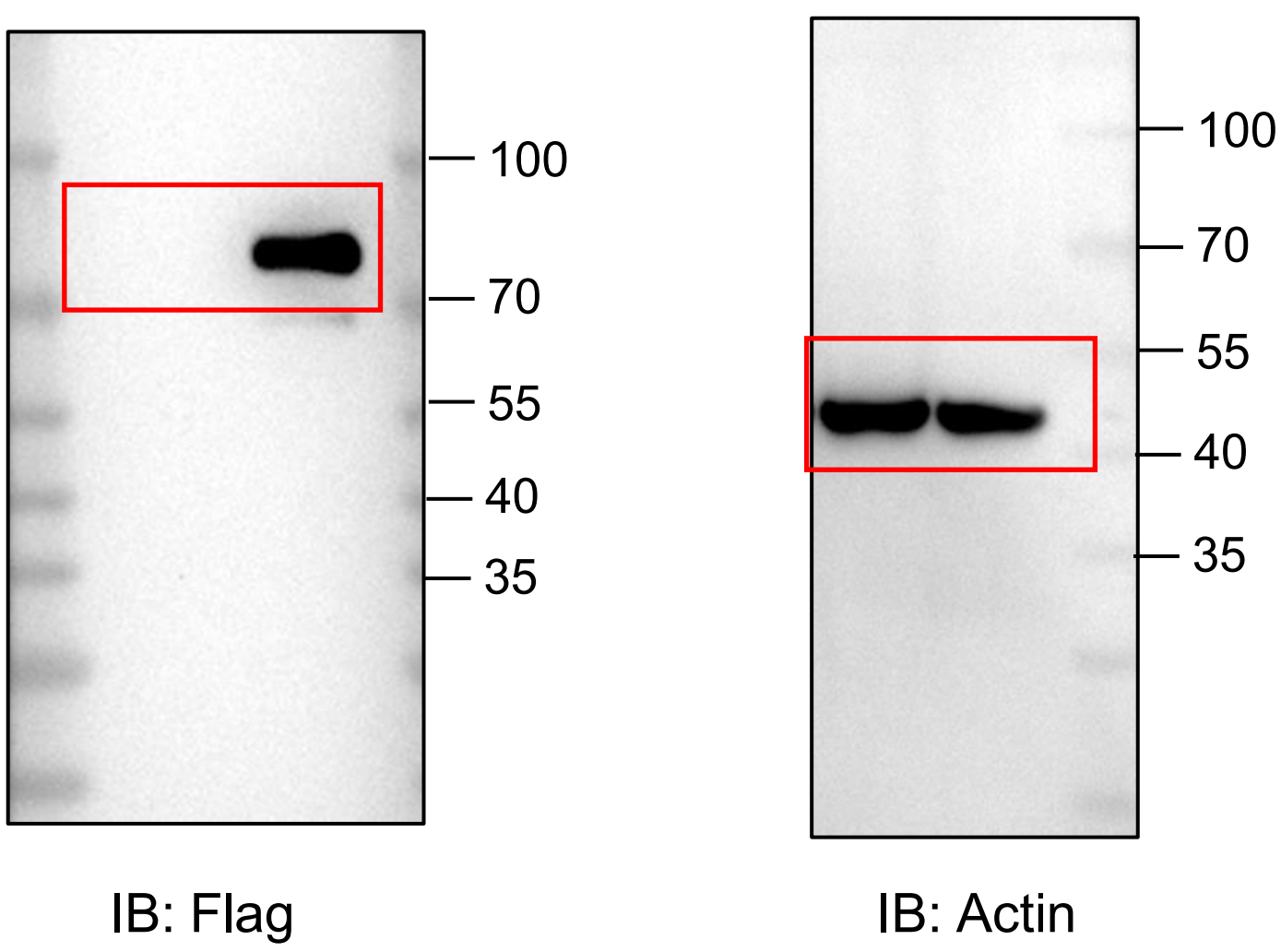

Supplementary Figure S2F

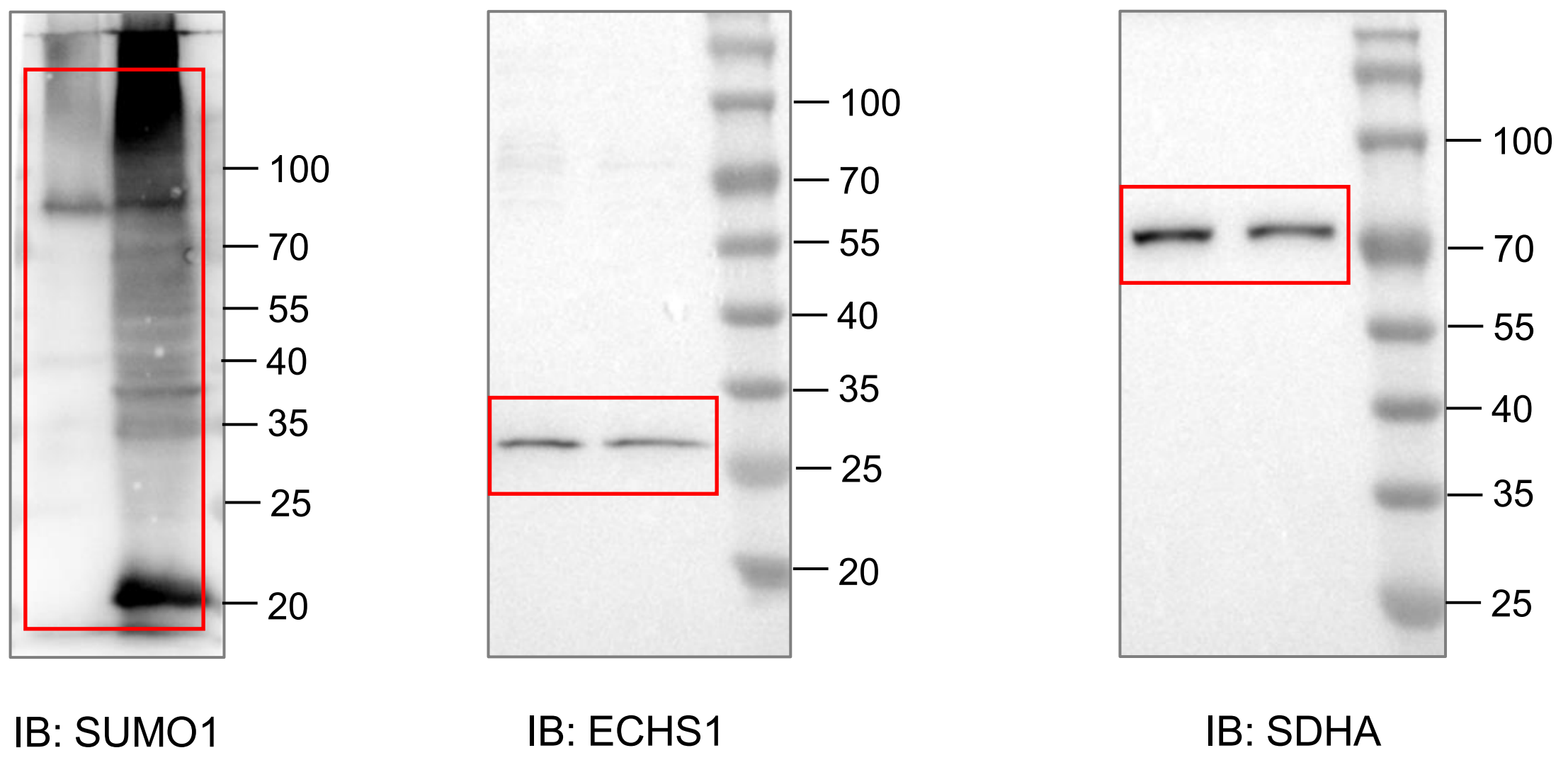

Supplementary Figure S3A

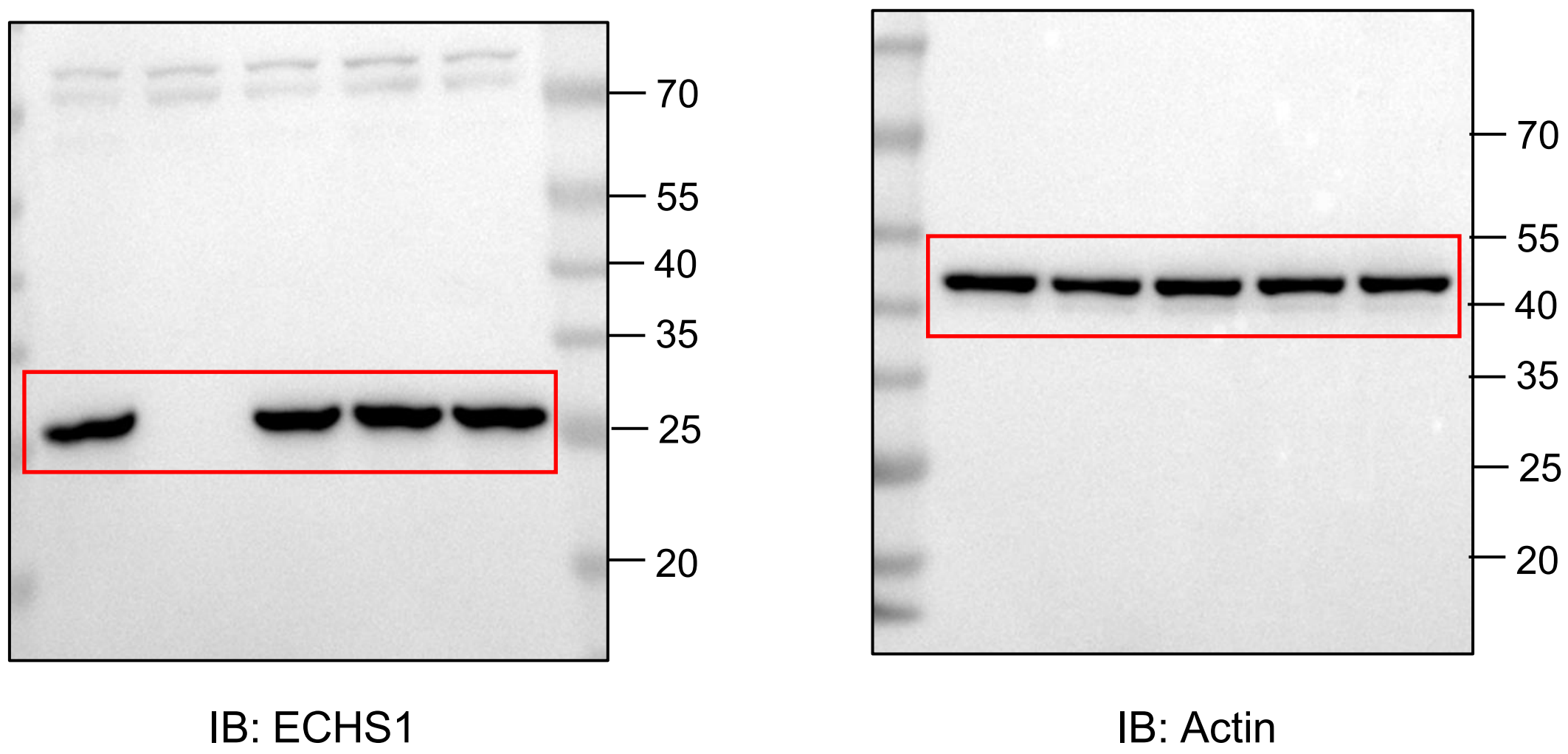

Supplementary Figure S3B

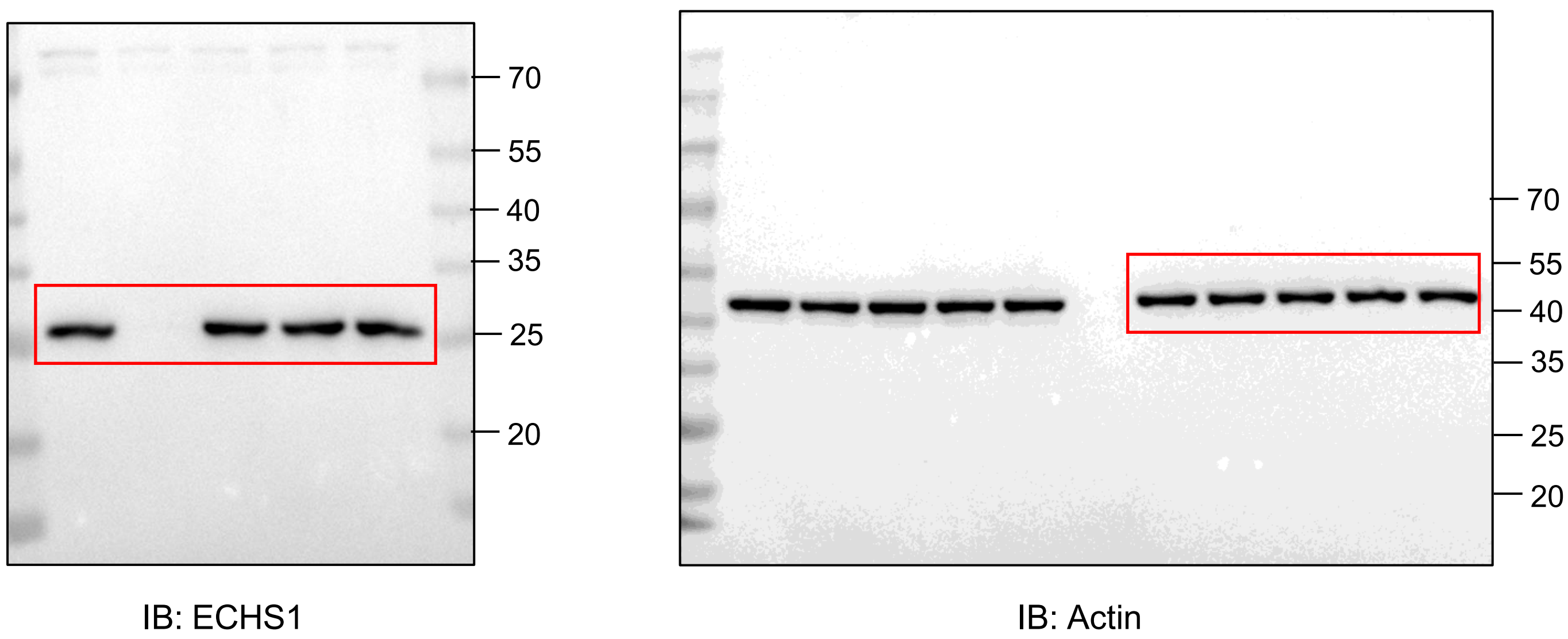

Supplementary Figure S3C

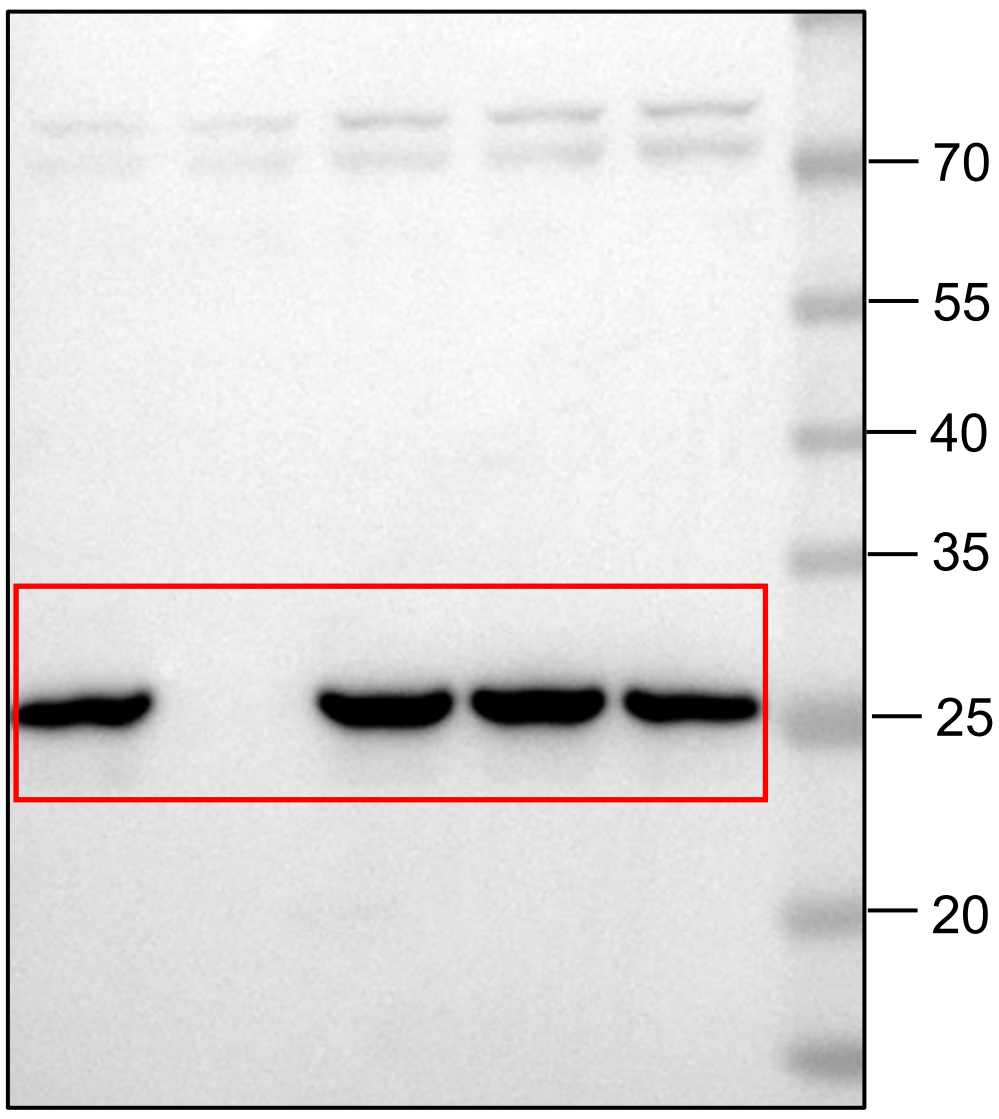

IB: ECHS1

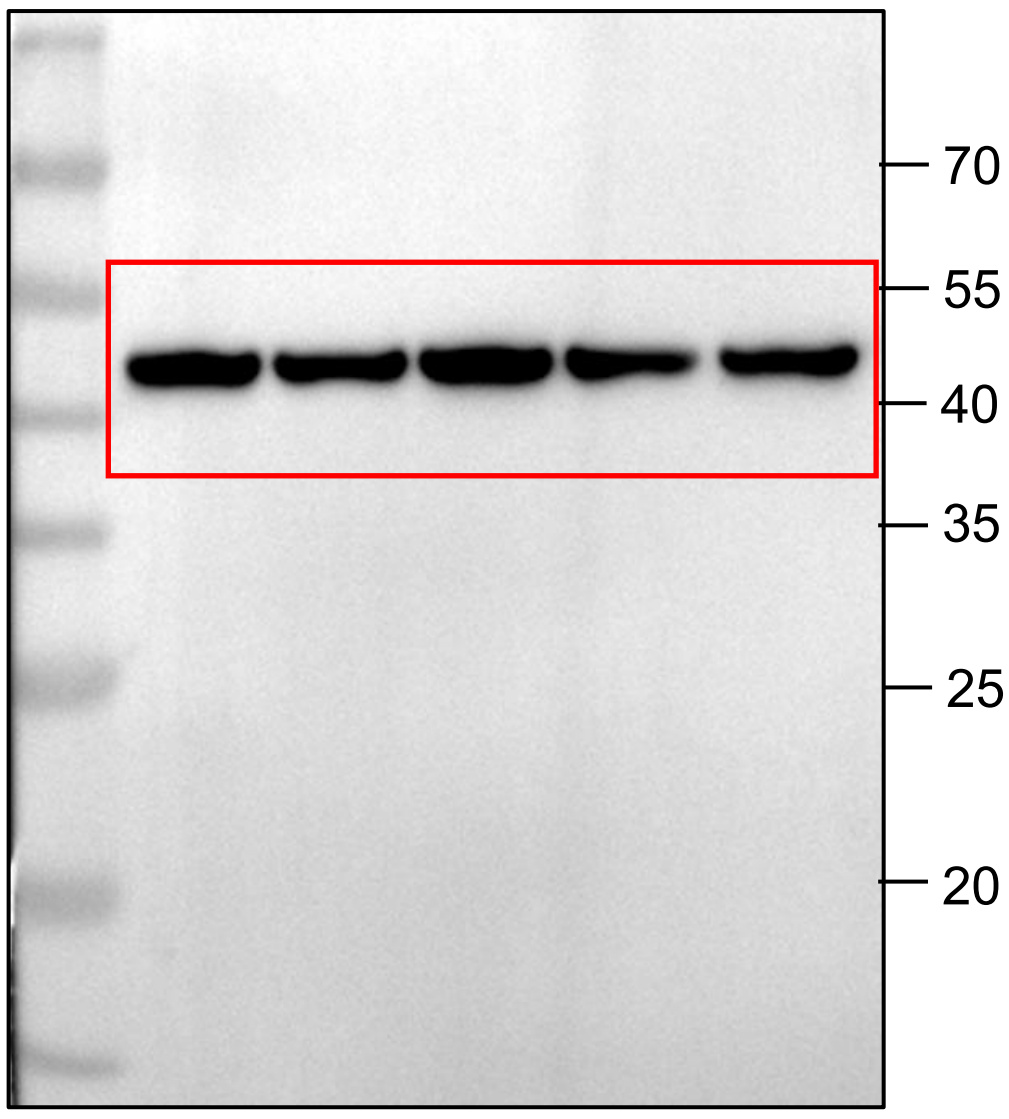

IB: Actin
