# Supplementary material for: SUMOylation activates ECHS1 for adaptive catabolism in lung cancer
Source: Cell Death Dis. 2026 Jul 9;17(1):854. doi: 10.1038/s41419-026-09078-1 (PMC13631240; doi:10.1038/s41419-026-09078-1)
Supplement: Supplementary file 2 [file 41419_2026_9078_MOESM12_ESM.pdf]

Corresponding Author Name: Jun Tu
Manuscript Number: CDDIS-26-009

SPRINGER NATURE

Reporting Summary

Springer Nature wishes to improve the reproducibility of the work that we publish. This checklist is used to ensure good reporting standards and to improve the reproducibility. Please respond completely to all questions relevant to your manuscript. For more information, please read the journal's Guide to Authors.

☑ **Check here to confirm that the following information is available in the Material & Methods section:**

- **The exact sample size (*n*)** for each experimental group/condition, given as a number, not a range
- **A description of the sample collection** allowing the reader to understand whether the samples represent technical or biological replicates (including how many animals, litters, culture, etc.)
- **A statement of how many times the experiment shown was replicated in the laboratory**
- **Definitions of statistical methods and measures**: For small sample sizes (n<5) descriptive statistics are not appropriate, instead plot individual data points
  - Very common tests, such as *t*-test, simple χ^2 tests, Wilcoxon and Mann-Whitney tests, can be unambiguously identified by name only, but more complex techniques should be described in the methods section
  - Are tests one-sided or two-sided?
  - Are there adjustments for multiple comparisons?
  - **Statistical test results**, e.g., ***P* values**
  - Definition of **'center values'** as **median or mean**;
  - Definition of **error bars as s.d. or s.e.m. or c.i.**

Please ensure that the answers to the following questions are reported in the manuscript itself. We encourage you to include a specific subsection in the methods section for statistics, reagents and animal models. Below, provide the page number or section and paragraph number.

Statistics and general methods

	**Reported in section/paragraph or page #**
1. How was the sample size chosen to ensure adequate power to detect a pre-specified effect size? (Give section/paragraph or page #)	For cell culture experiments, n=3 biological replicates per group. For animal studies, 6 mice per group based on preliminary experiments and similar published studies. Materials and Methods 2.12-2.14; Figure Legends
For animal studies, include a statement about sample size estimate even if no statistical methods were used.	The sample size was 6 mice per group, this sample size was chosen based on preliminary experiments and similar published studies.
2. Describe inclusion/exclusion criteria if samples or animals were excluded from the analysis. Were the criteria pre-established? (Give section/paragraph or page #)	No animals or samples were excluded from the final analysis in this study. All experimental subjects and collected samples that completed the scheduled interventions were included for subsequent data
3. If a method of randomization was used to determine how samples/animals were allocated to experimental groups and processed, describe it. (Give section/paragraph or page #)	Mice were randomly assigned to normal diet or lysine/tryptophan-restriction diet groups. Materials and Methods 2.14
For animal studies, include a statement about randomization even if no randomization was used.	For the animal studies, mice were randomly assigned to experimental groups. Specifically, they were randomly allocated to either a normal diet or a lysine/tryptophan-

4. If the investigator was blinded to the group allocation during the experiment and/or when assessing the outcome, state the extent of blinding. (Give section/paragraph or page #)	Blinding was implemented throughout this study. The researchers performing animal treatments and outcome quantification were fully blinded to group allocation during experimental manipulation and all phenotypic
For animal studies, include a statement about blinding even if no blinding was done.	Blinding was implemented throughout this study. The researchers performing animal treatments and outcome
5. For every figure, are statistical tests justified as appropriate?	Statistical analysis performed using GraphPad Prism 10.0. For multiple comparisons, Dunnett or Šídák tests were used; for two-group comparisons, t-test was applied. Data
Do the data meet the assumptions of the tests (e.g., normal distribution)?	The data generally meet the statistical assumptions of the performed tests.
Is there an estimate of variation within each group of data?	Yes, the variation within each experimental group was appropriately estimated and reported in this study. Quantitative data are presented with measures of
Is the variance similar between the groups that are being statistically compared? (Give section/paragraph or page #)	Yes, the variance is similar (homogeneous) between all groups that were subjected to statistical comparison in our study. Figure 2-6

Reagents

Reported in section/paragraph or page #

6. Report the source of antibodies (vendor and catalog number)	See Supplementary Table S1 for catalog numbers and vendors. Materials and Methods 2.1
7. Identify the source of cell lines and report if they were recently authenticated (e.g., by STR profiling) and tested for mycoplasma contamination	HEK293T and A549 cells were purchased from Chinese Academy of Sciences (Shanghai, China). PC-9 cells were gifted by Prof. Jianxiu Yu, Shanghai Jiao Tong University. Cell authentication via short tandem repeat (STR) profiling

Animal Models

Reported in section/paragraph or page #

8. Report species, strain, sex and age of animals	BALB/c nude mice; 5-week-old; males; Source: Shanghai
9. For experiments involving live vertebrates, include a statement of compliance with ethical regulations and identify the committee(s) approving the experiments.	All procedures performed in compliance with ARRIVE 2.0 guidelines and approved by Shanghai Jiao Tong University School of Medicine (Approval No. JUMC2023-120-A). Materials and Methods 2.14

10. We recommend consulting the ARRIVE guidelines (*PLoS Biol.* **8**(6), e1000412,2010) to ensure that other relevant aspects of animal studies are adequately reported.

Human subjects

	Reported in section/paragraph or page #
11. Identify the committee(s) approving the study protocol.	Human lung adenocarcinoma tissue microarrays (WeiaoBio #ZL-LUC1601), The human lung adenocarcinoma tissue
12. Include a statement confirming that informed consent was obtained from all subjects.	Written informed consent was obtained from all participants prior to tissue sample collection.
13. For publication of patient photos, include a statement confirming that consent to publish was obtained.	Not applicable - no patient photos published.
14. Report the clinical trial registration number (at ClinicalTrials.gov or equivalent).	Not applicable - not a clinical trial.

15. For phase II and III randomized controlled trials, please refer to the CONSORT statement and submit the CONSORT checklist with your submission.

16. For tumor marker prognostic studies, we recommend that you follow the REMARK reporting guidelines.

Data deposition

	Reported in section/paragraph or page #
17. Provide accession codes for deposited data. Data deposition in a public repository is mandatory for:<br>a. Protein, DNA and RNA sequences<br>b. Macromolecular structures<br>c. Crystallographic data for small molecules<br>d. Microarray data	Omics data deposited in NODE database, Accession number: OEP00006978 (https://www.biosino.org/node/project/detail/OEP00006978).<br>Data Availability Statement

Deposition is strongly recommended for many other datasets for which structured public repositories exist; more details on our data policy are available in the Guide to Authors. We encourage the provision of other source data in supplementary information or in unstructured repositories such as Figshare and Dryad. We encourage publication of Data Descriptors (see Scientific Data) to maximize data reuse.

18. If computer code was used to generate results that are central to the paper's conclusions, include a statement in the Methods section under "**Code availability**" to indicate whether and how the code can be accessed. Include version information as necessary and any restrictions on availability.	No computer code was used.
