# Supplementary material for: SUMOylation activates ECHS1 for adaptive catabolism in lung cancer
Source: Cell Death Dis. 2026 Jul 9;17(1):854. doi: 10.1038/s41419-026-09078-1 (PMC13631240; doi:10.1038/s41419-026-09078-1)
Supplement: Supplementary file 3 [file 41419_2026_9078_MOESM2_ESM.pdf]

ECHS1 amino acid sequence:

```
  1 MAALRVLLSC VRGPLRPPVR CPAWRPFASG ANFEYIIAEK RGKNNTVGLI QLNRPKALNA
 61 LCDGLIDELN QALKTFEEDP AVGAIVLTGG DKAFAAGADI KEMQNLSFQD CYSSKFLKHW
121 DHLTQVKKPV IAAVNGYAFG GGCELAMMCD IIYAGEKAQF AQPEILIGTI PGAGGTQRLT
181 RAVGKSLAME MVLTGDRISA QDAKQAGLVS KICPVETLVE EAIQCAEKIA SNSKIVVAMA
241 KESVNAAFEM TLTEGSKLEK KLFYSTFATD DRKEGMTAFV EKRKANFKDQ
```

Red text: K128 SUMOylation site

Underline: KxΨ motif
