# Supplementary material for: SUMOylation activates ECHS1 for adaptive catabolism in lung cancer
Source: Cell Death Dis. 2026 Jul 9;17(1):854. doi: 10.1038/s41419-026-09078-1 (PMC13631240; doi:10.1038/s41419-026-09078-1)
Supplement: Supplementary file 4 [file 41419_2026_9078_MOESM3_ESM.pdf]

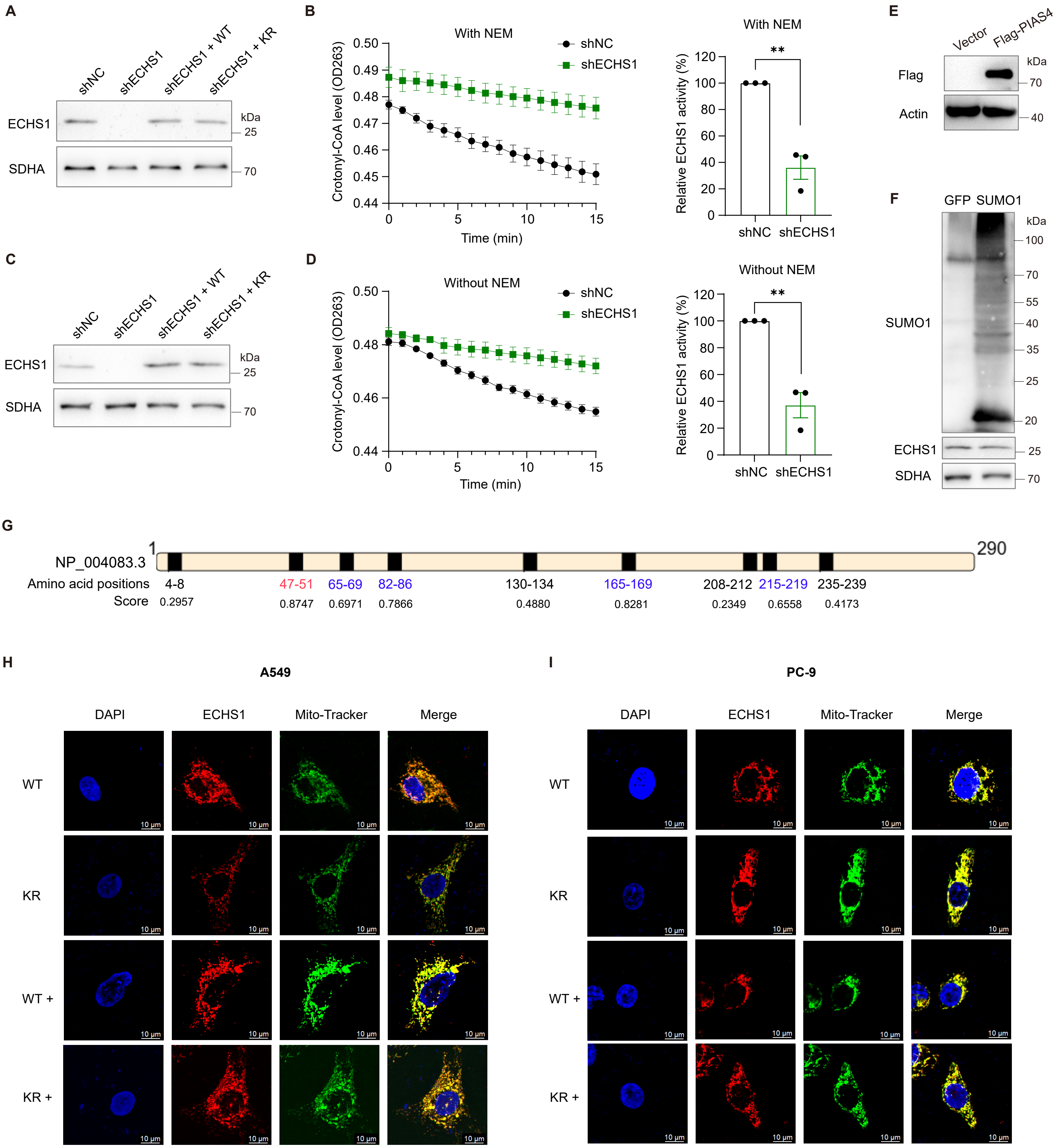
A
shNC
shECHS1
shECHS1 + WT
shECHS1 + KR
ECHS1
SDHA
kDa
25
70
B
With NEM
shNC
shECHS1
Crotonyl-CoA level (OD263)
Time (min)
Relative ECHS1 activity (%)
**
C
D
Without NEM
E
Vector
Flag-PIAS4
Flag
Actin
F
GFP SUMO1
SUMO1
G
NP_004083.3
1
290
Amino acid positions
Score
4-8 0.2957
47-51 0.8747
65-69 0.6971
82-86 0.7866
130-134 0.4880
165-169 0.8281
208-212 0.2349
215-219 0.6558
235-239 0.4173
H
A549
I
PC-9
DAPI
ECHS1
Mito-Tracker
Merge
WT
KR
WT +
KR +
10 μm
