# Supplementary material for: SUMOylation activates ECHS1 for adaptive catabolism in lung cancer
Source: Cell Death Dis. 2026 Jul 9;17(1):854. doi: 10.1038/s41419-026-09078-1 (PMC13631240; doi:10.1038/s41419-026-09078-1)
Supplement: Supplementary file 5 [file 41419_2026_9078_MOESM4_ESM.pdf]

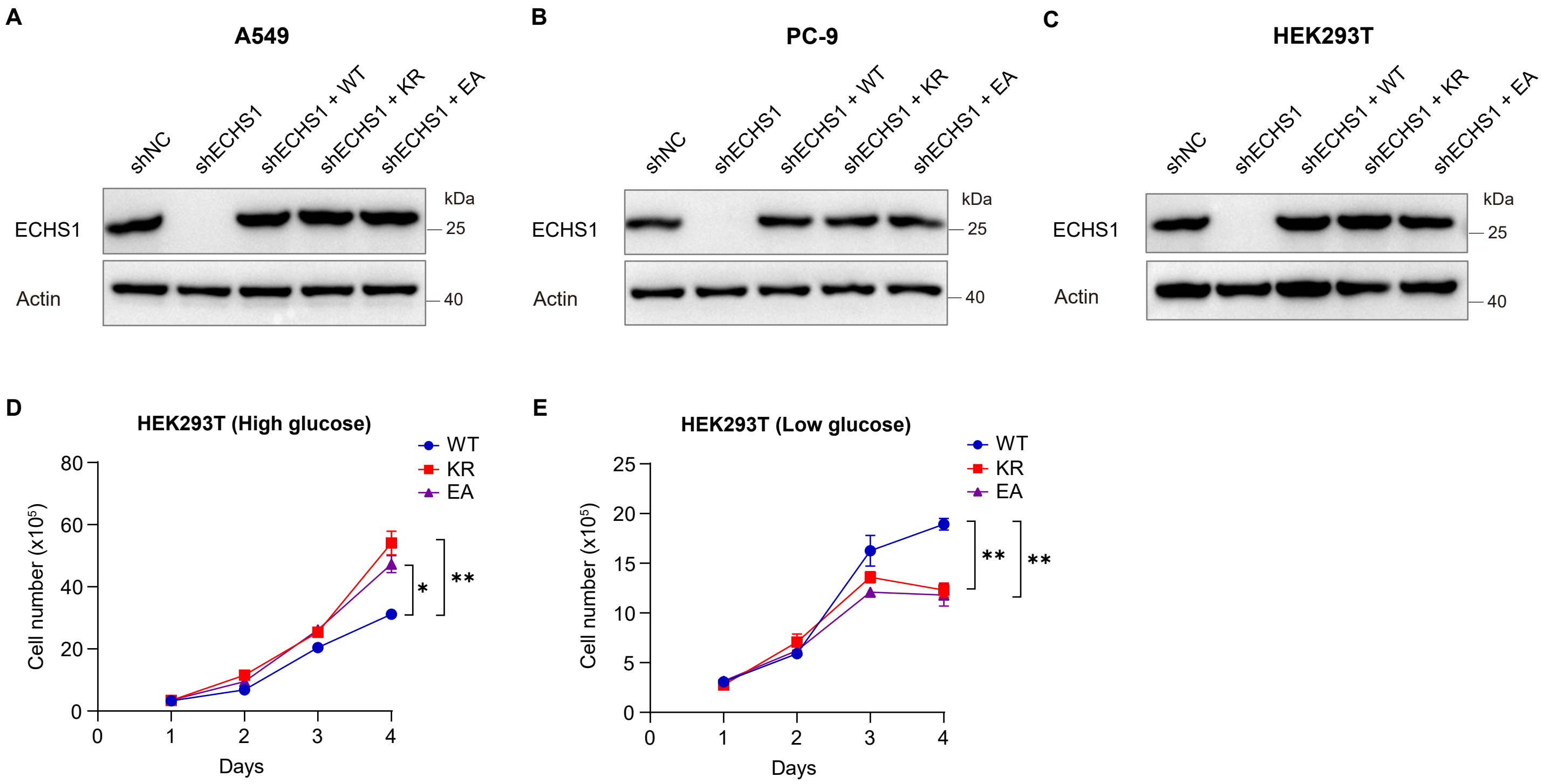
A
A549
shNC
shECHS1
shECHS1 + WT
shECHS1 + KR
shECHS1 + EA
kDa
ECHS1
25
Actin
40
B
PC-9
shNC
shECHS1
shECHS1 + WT
shECHS1 + KR
shECHS1 + EA
kDa
ECHS1
25
Actin
40
C
HEK293T
shNC
shECHS1
shECHS1 + WT
shECHS1 + KR
shECHS1 + EA
kDa
ECHS1
25
Actin
40
D
HEK293T (High glucose)
WT
KR
EA
Cell number (x10⁵)
0
20
40
60
80
*
**
0
1
2
3
4
Days
E
HEK293T (Low glucose)
WT
KR
EA
Cell number (x10⁵)
0
5
10
15
20
25
**
**
0
1
2
3
4
Days
