# Supplementary material for: SUMOylation activates ECHS1 for adaptive catabolism in lung cancer
Source: Cell Death Dis. 2026 Jul 9;17(1):854. doi: 10.1038/s41419-026-09078-1 (PMC13631240; doi:10.1038/s41419-026-09078-1)
Supplement: Supplementary file 6 [file 41419_2026_9078_MOESM5_ESM.pdf]

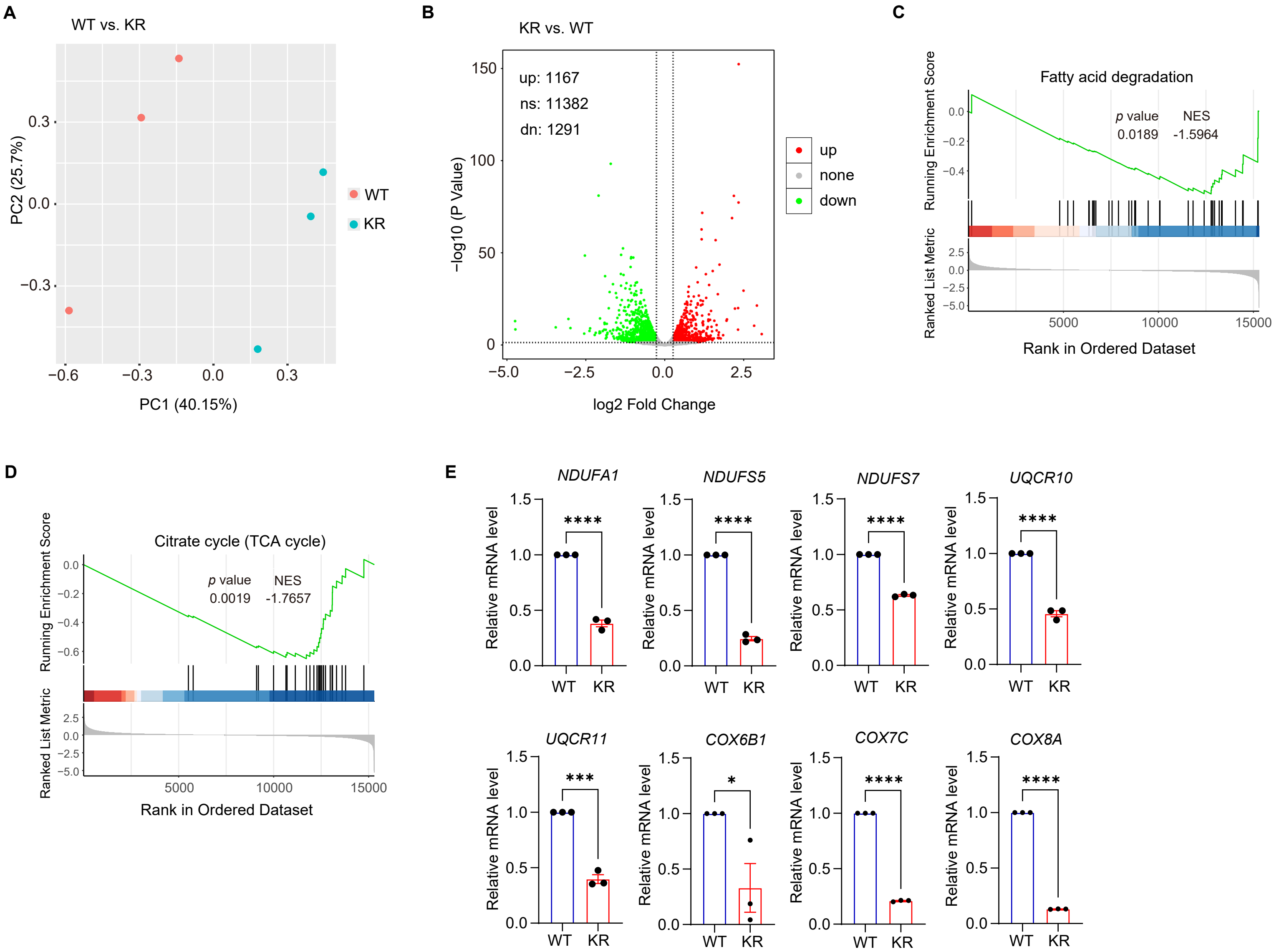
A
WT vs. KR
PC1 (40.15%)
PC2 (25.7%)
WT
KR
B
KR vs. WT
up: 1167
ns: 11382
dn: 1291
up
none
down
log2 Fold Change
−log10 (P Value)
C
Fatty acid degradation
p value 0.0189
NES -1.5964
Running Enrichment Score
Ranked List Metric
Rank in Ordered Dataset
D
Citrate cycle (TCA cycle)
p value 0.0019
NES -1.7657
Running Enrichment Score
Ranked List Metric
Rank in Ordered Dataset
E
NDUFA1
NDUFS5
NDUFS7
UQCR10
UQCR11
COX6B1
COX7C
COX8A
Relative mRNA level
WT
KR
****
***
*
