# Supplementary material for: SUMOylation activates ECHS1 for adaptive catabolism in lung cancer
Source: Cell Death Dis. 2026 Jul 9;17(1):854. doi: 10.1038/s41419-026-09078-1 (PMC13631240; doi:10.1038/s41419-026-09078-1)
Supplement: Supplementary file 7 [file 41419_2026_9078_MOESM6_ESM.pdf]

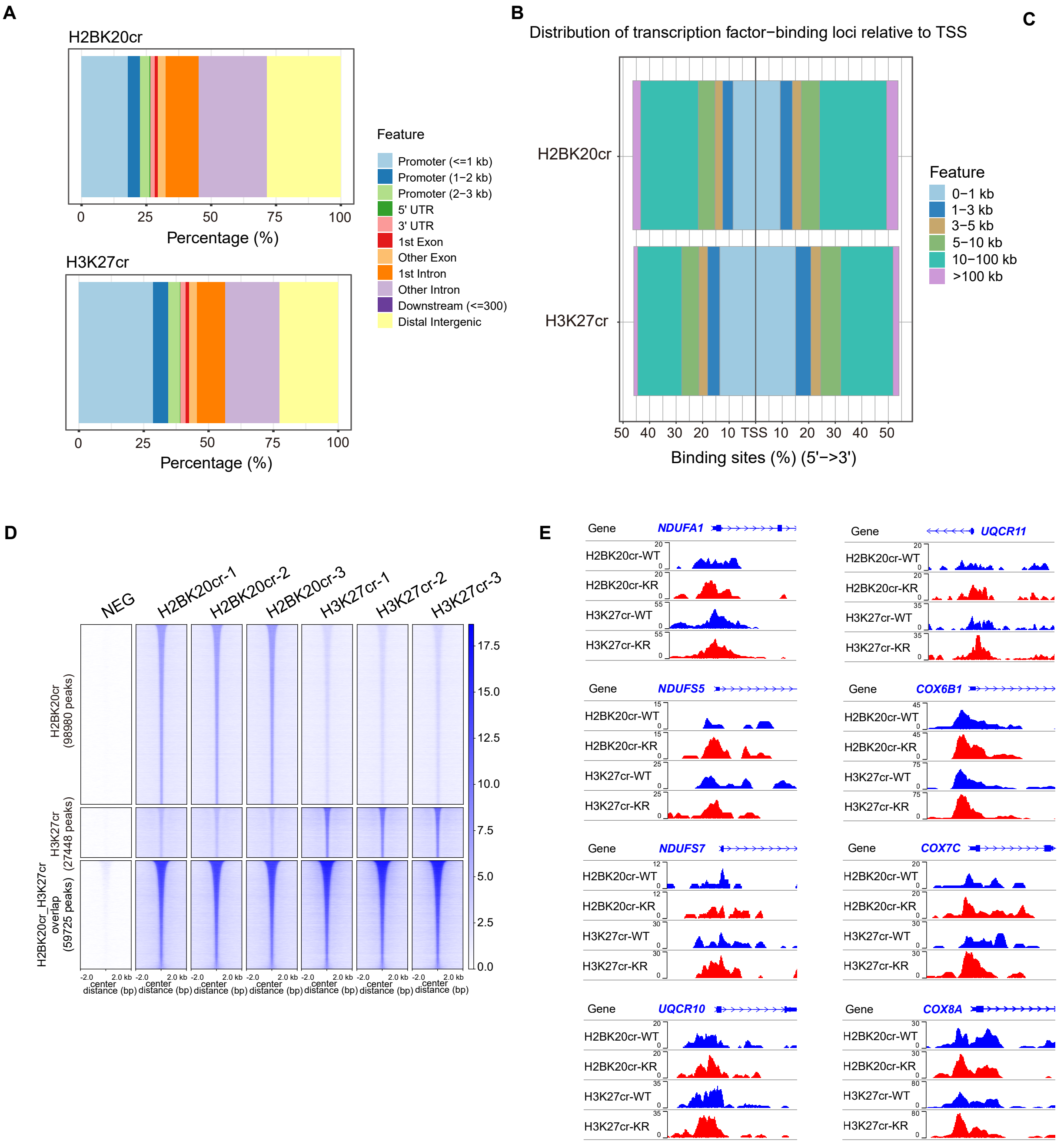
A
H2BK20cr
0
25
50
75
100
Percentage (%)
H3K27cr
Percentage (%)
Feature
Promoter (<=1 kb)
Promoter (1−2 kb)
Promoter (2−3 kb)
5' UTR
3' UTR
1st Exon
Other Exon
1st Intron
Other Intron
Downstream (<=300)
Distal Intergenic
B
Distribution of transcription factor−binding loci relative to TSS
H2BK20cr
H3K27cr
50 40 30 20 10 TSS 10 20 30 40 50
Binding sites (%) (5'−>3')
Feature
0−1 kb
1−3 kb
3−5 kb
5−10 kb
10−100 kb
>100 kb
C
H2BK20cr
H3K27cr
421
(21.0%)
1094
(54.7%)
486
(24.3%)
D
NEG
H2BK20cr-1
H2BK20cr-2
H2BK20cr-3
H3K27cr-1
H3K27cr-2
H3K27cr-3
H2BK20cr
(98980 peaks)
H3K27cr
(27448 peaks)
H2BK20cr_H3K27cr
overlap
(59725 peaks)
17.5
15.0
12.5
10.0
7.5
5.0
2.5
0.0
-2.0 center 2.0 kb
distance (bp)
E
Gene
NDUFA1
UQCR11
NDUFS5
COX6B1
NDUFS7
COX7C
UQCR10
COX8A
H2BK20cr-WT
H2BK20cr-KR
H3K27cr-WT
H3K27cr-KR
