# Supplementary material for: SUMOylation activates ECHS1 for adaptive catabolism in lung cancer
Source: Cell Death Dis. 2026 Jul 9;17(1):854. doi: 10.1038/s41419-026-09078-1 (PMC13631240; doi:10.1038/s41419-026-09078-1)
Supplement: Supplementary file 8 [file 41419_2026_9078_MOESM7_ESM.pdf]

Figure 1A

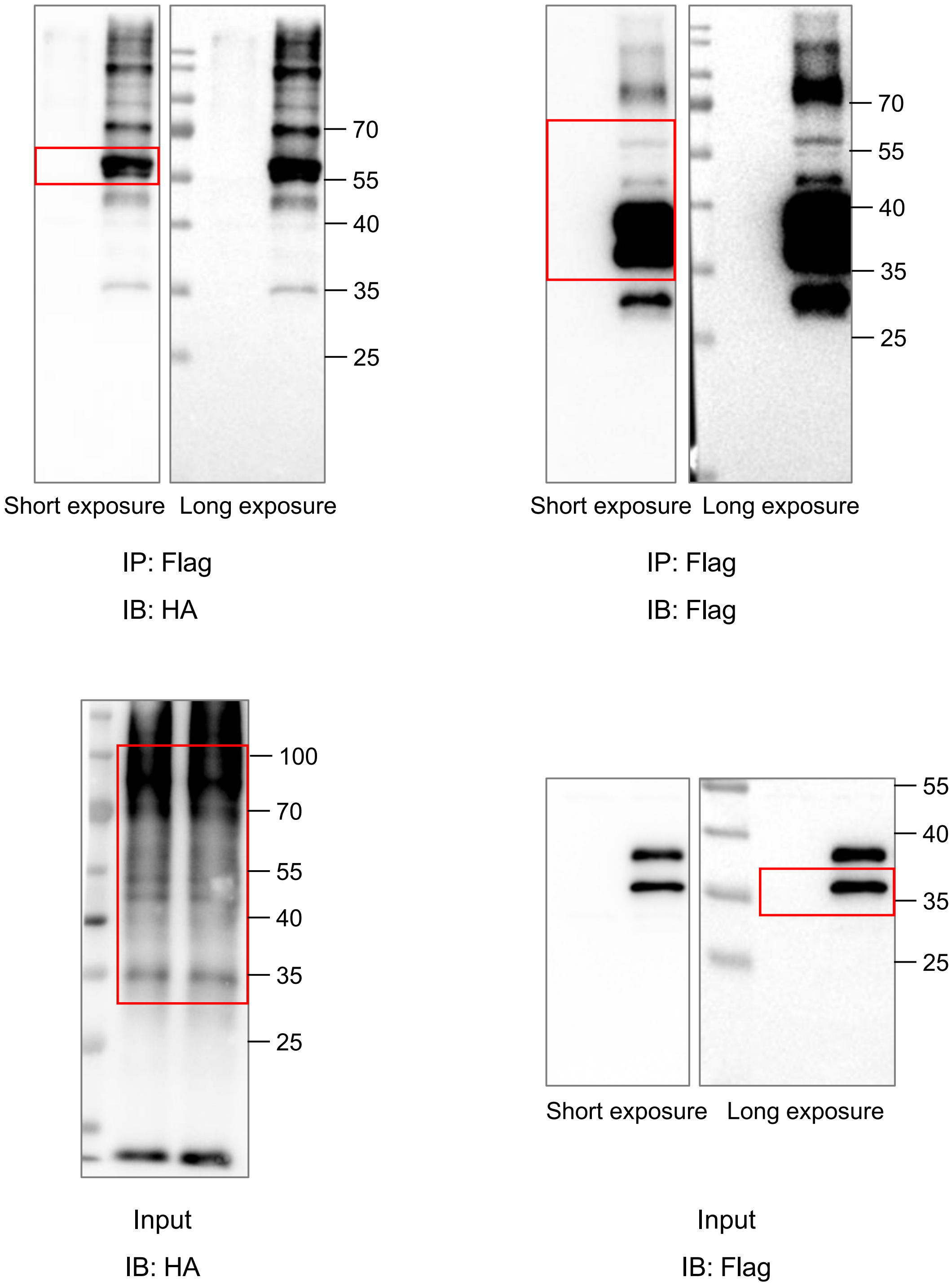

70
55
40
35
25
Short exposure
Long exposure
IP: Flag
IB: HA
70
55
40
35
25
Short exposure
Long exposure
IP: Flag
IB: Flag
100
70
55
40
35
25
Input
IB: HA
55
40
35
25
Short exposure
Long exposure
Input
IB: Flag

Figure 1B

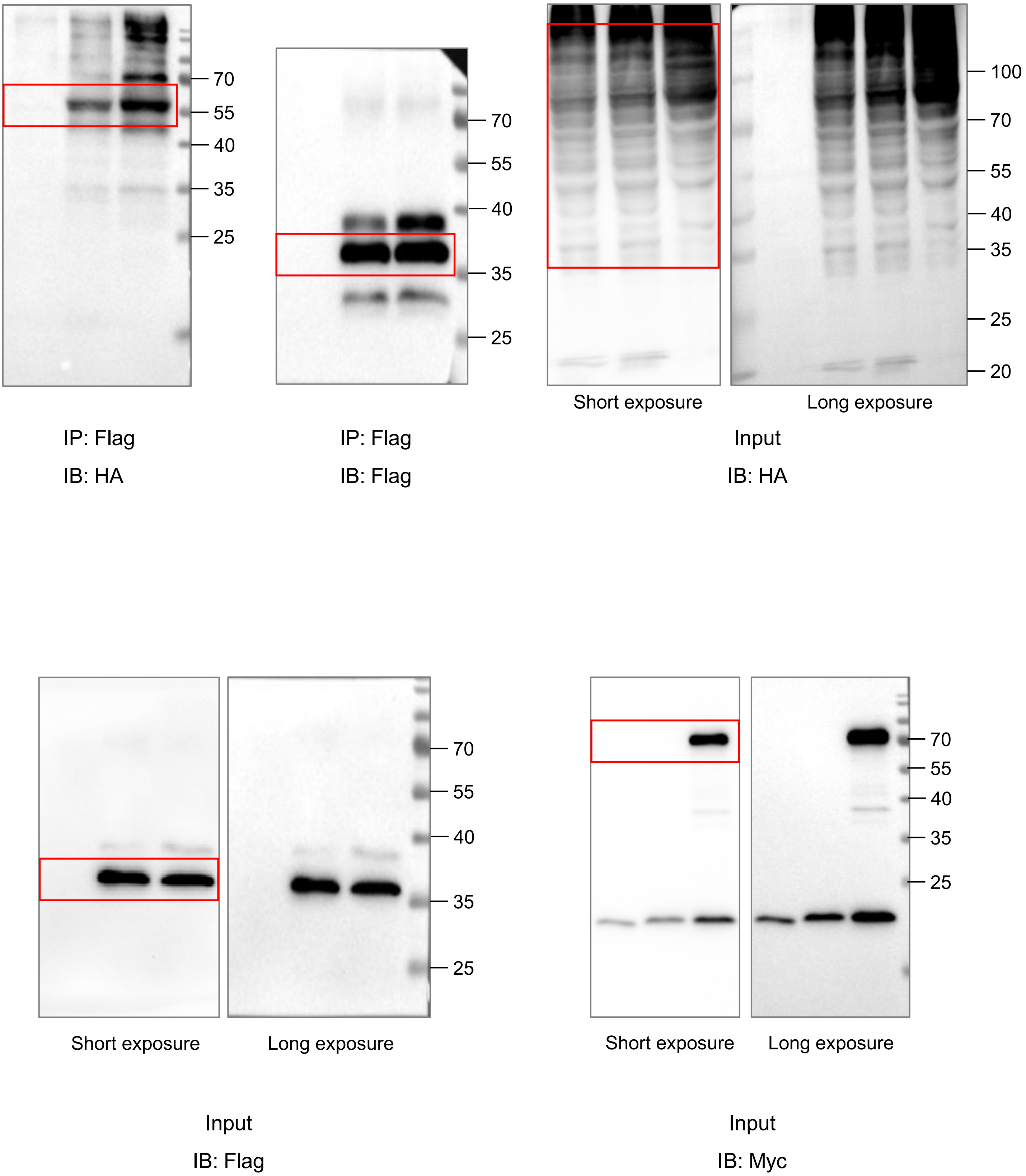
70
55
40
35
25
IP: Flag
IB: HA
70
55
40
35
25
IP: Flag
IB: Flag
Short exposure
Long exposure
100
70
55
40
35
25
20
Input
IB: HA
70
55
40
35
25
Short exposure
Long exposure
Input
IB: Flag
70
55
40
35
25
Short exposure
Long exposure
Input
IB: Myc

Figure 1C

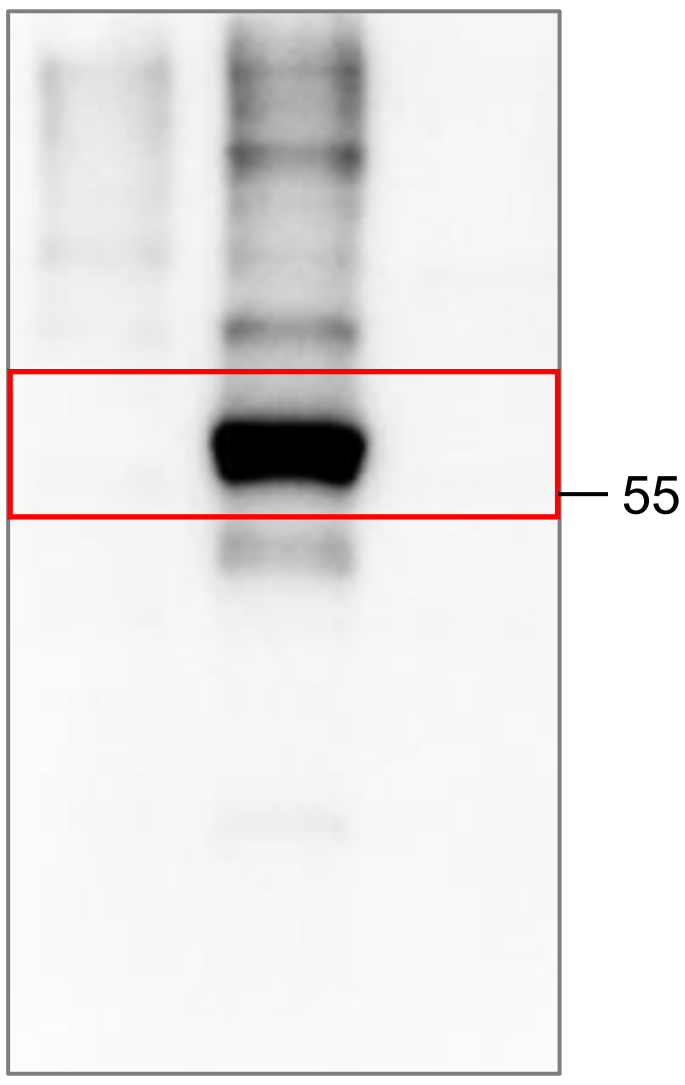

IP: Flag
IB: HA

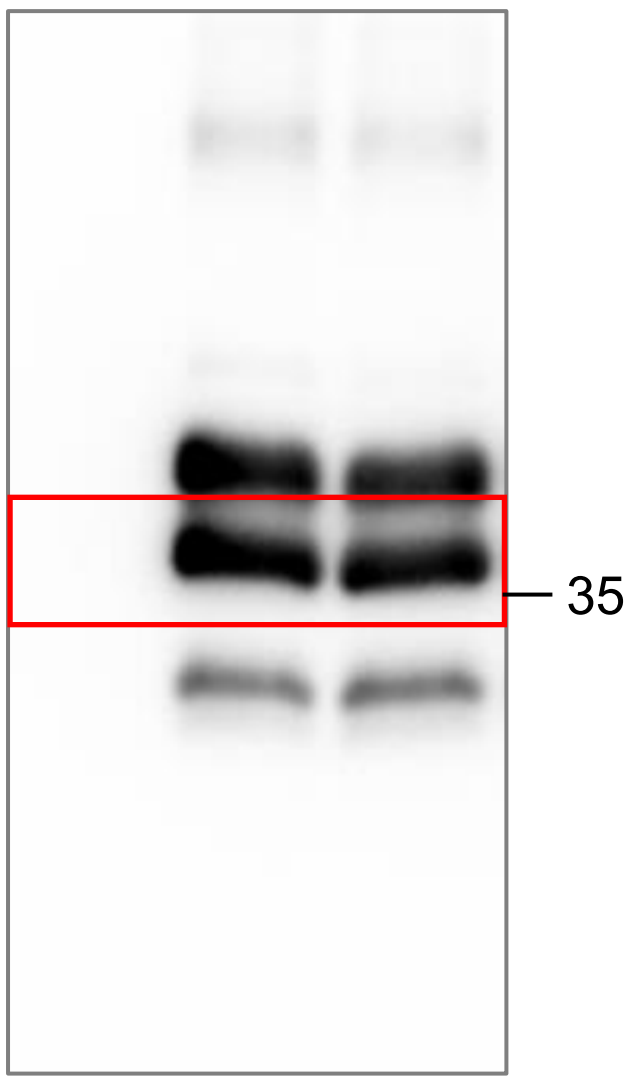

IP: Flag
IB: Flag

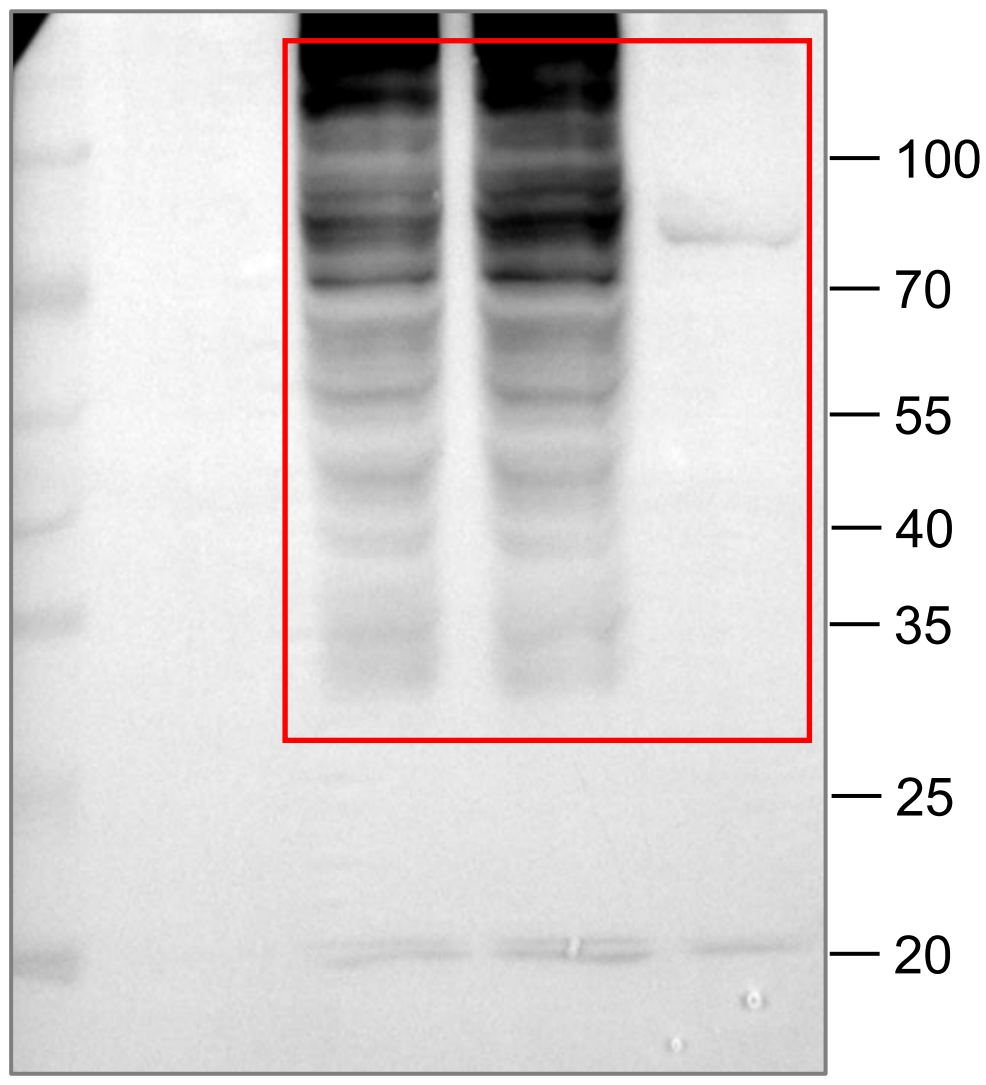

Input
IB: HA

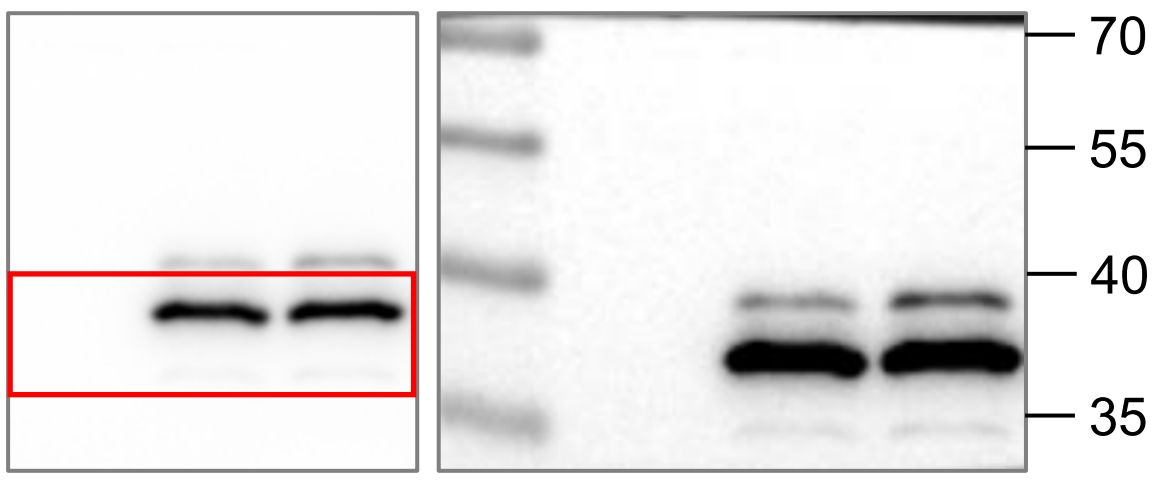

Short exposure Long exposure

Input
IB: Flag

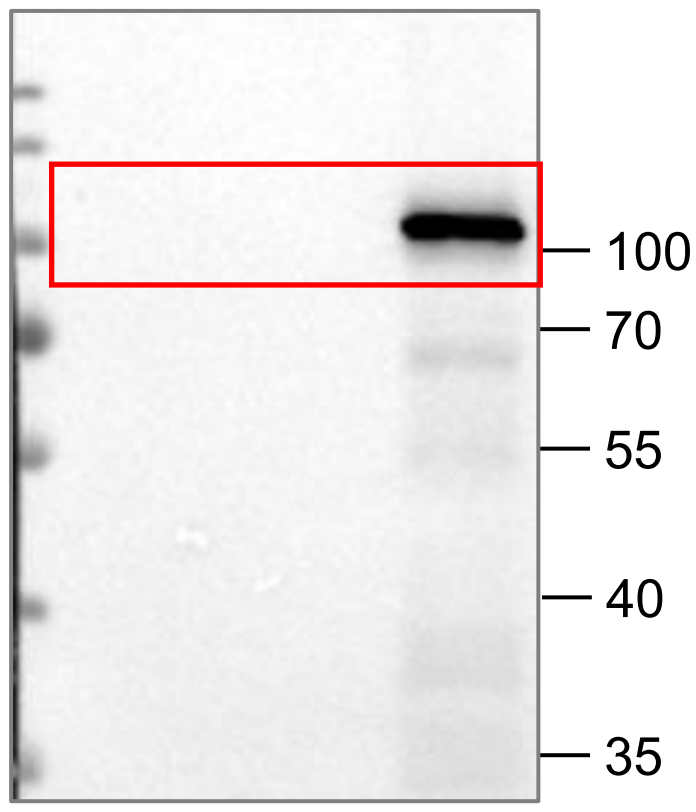

Input
IB: GFP

Figure 1D

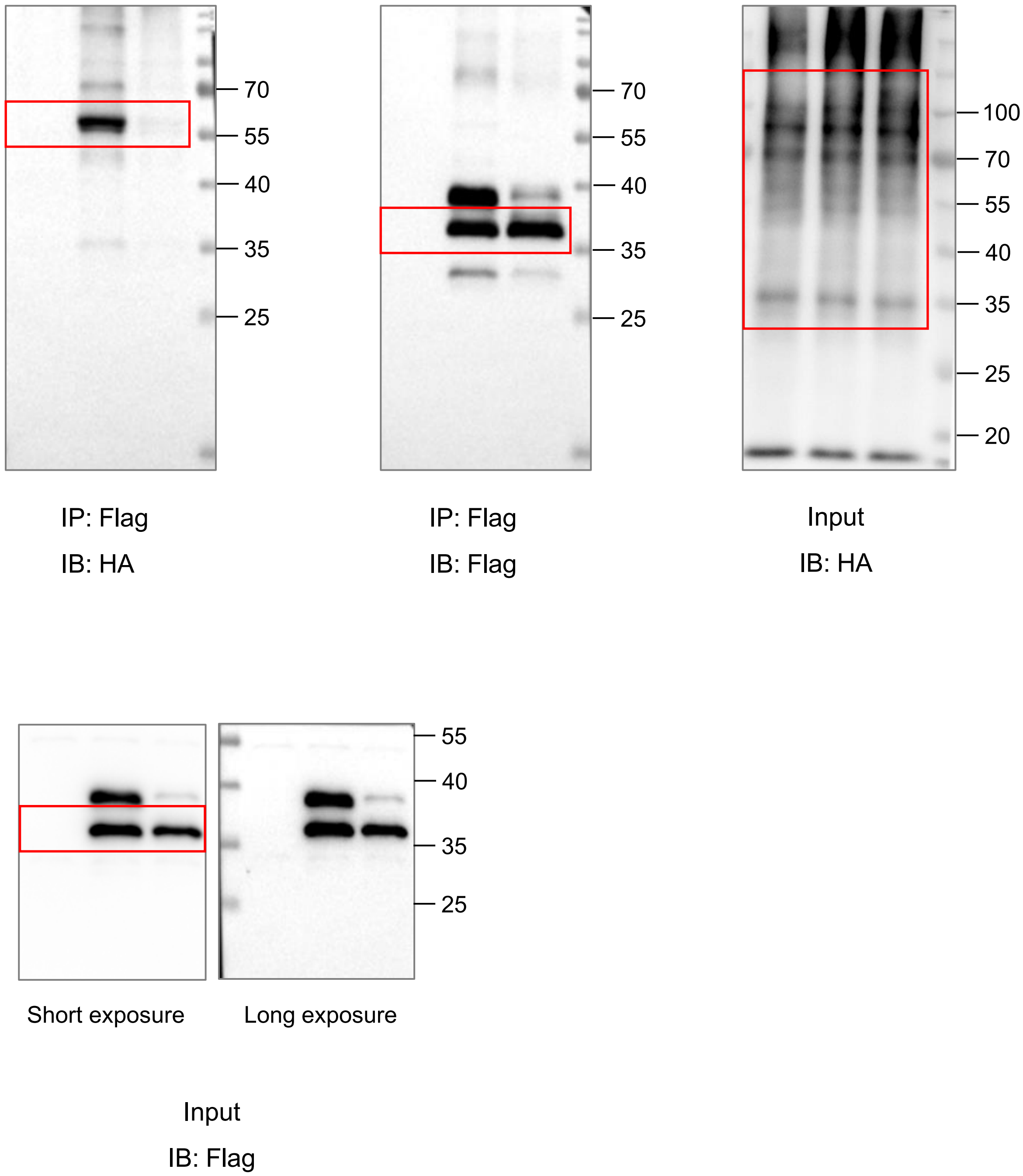
70
55
40
35
25
IP: Flag
IB: HA
70
55
40
35
25
IP: Flag
IB: Flag
100
70
55
40
35
25
20
Input
IB: HA
55
40
35
25
Short exposure
Long exposure
Input
IB: Flag

Figure 1E

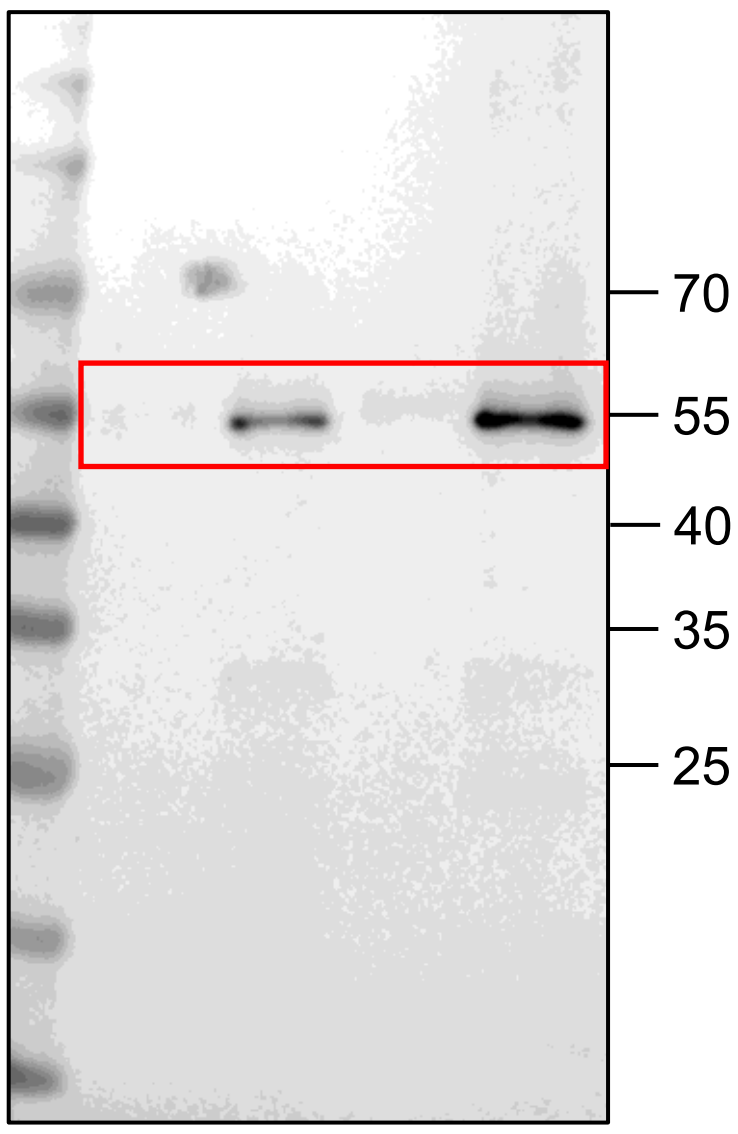

IP: IgG/SUMO1

IB: ECHS1

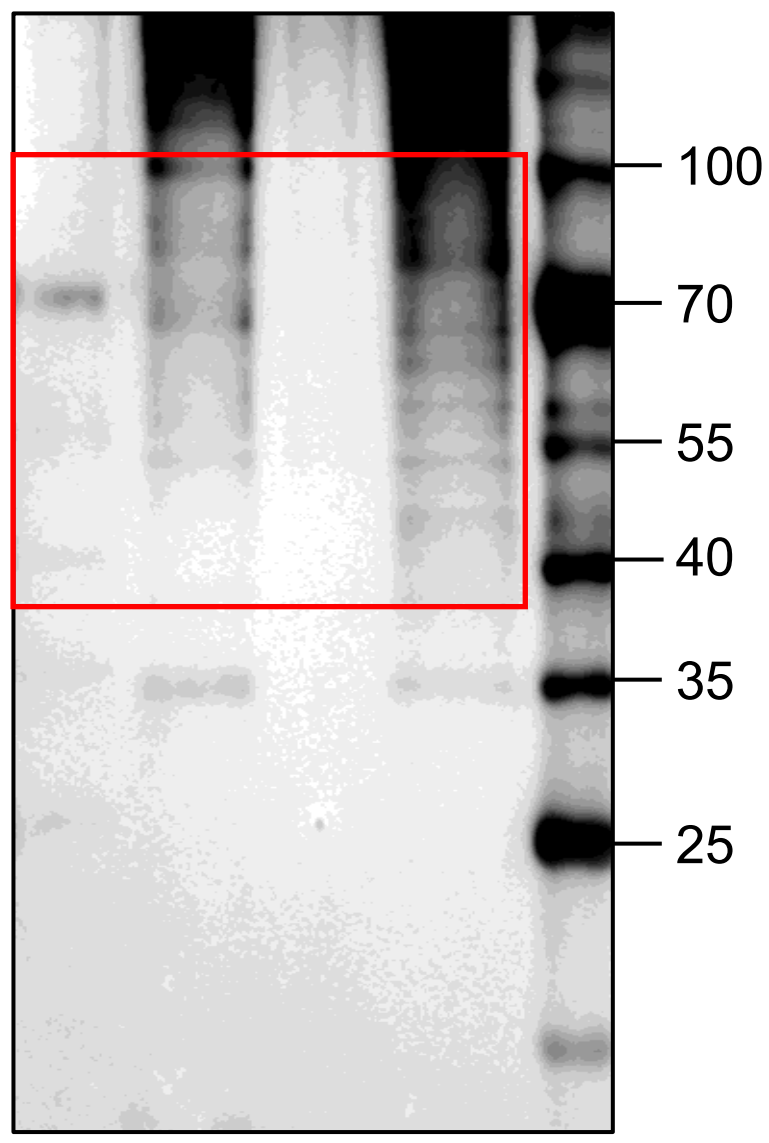

IP: IgG/SUMO1

IB: SUMO1

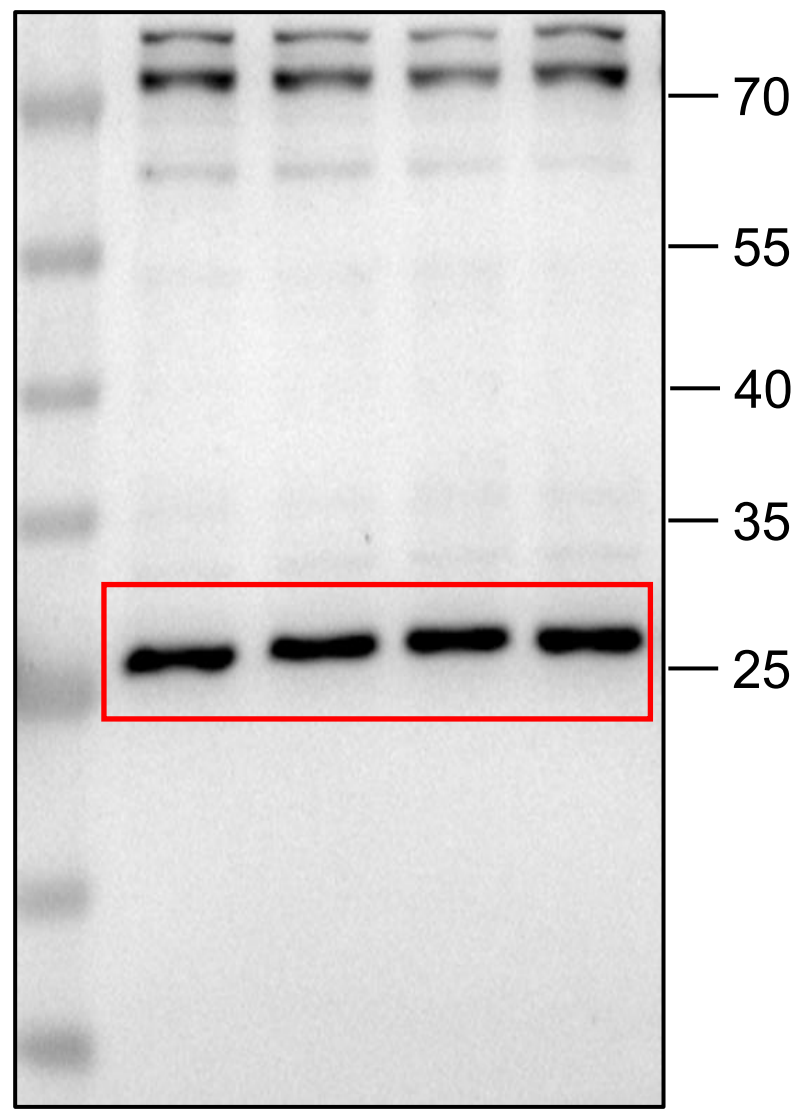

Input

IB: ECHS1

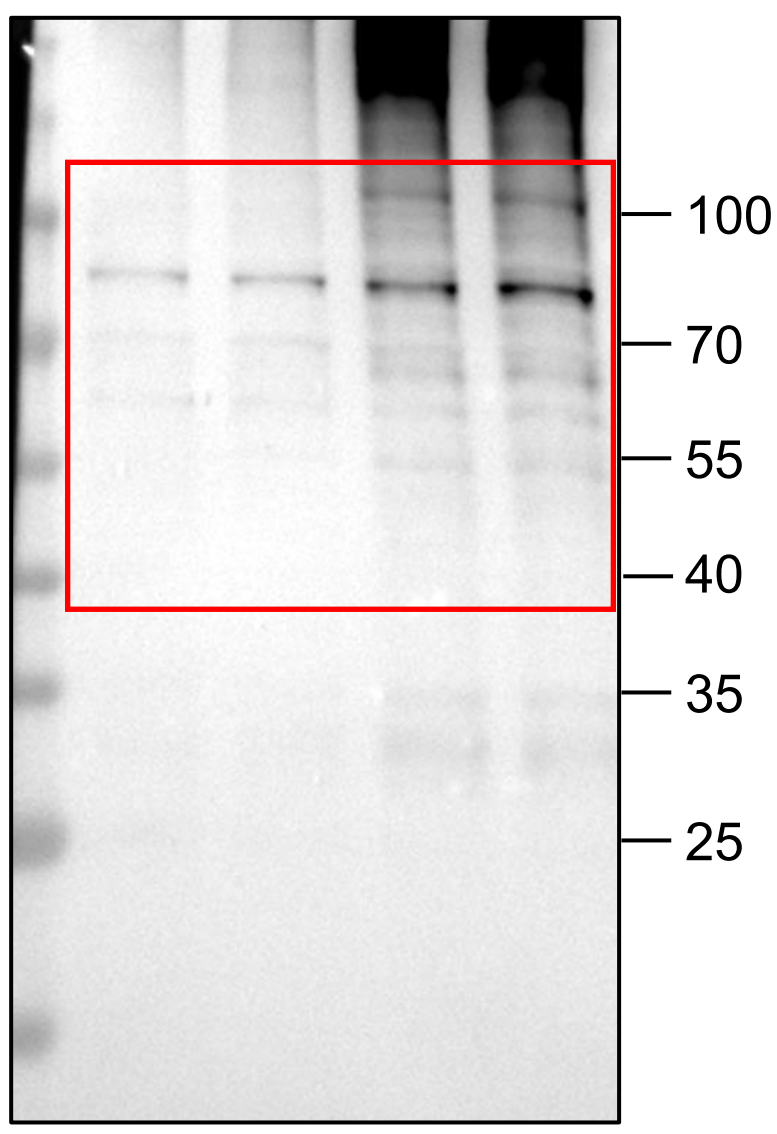

Input

IB: SUMO1

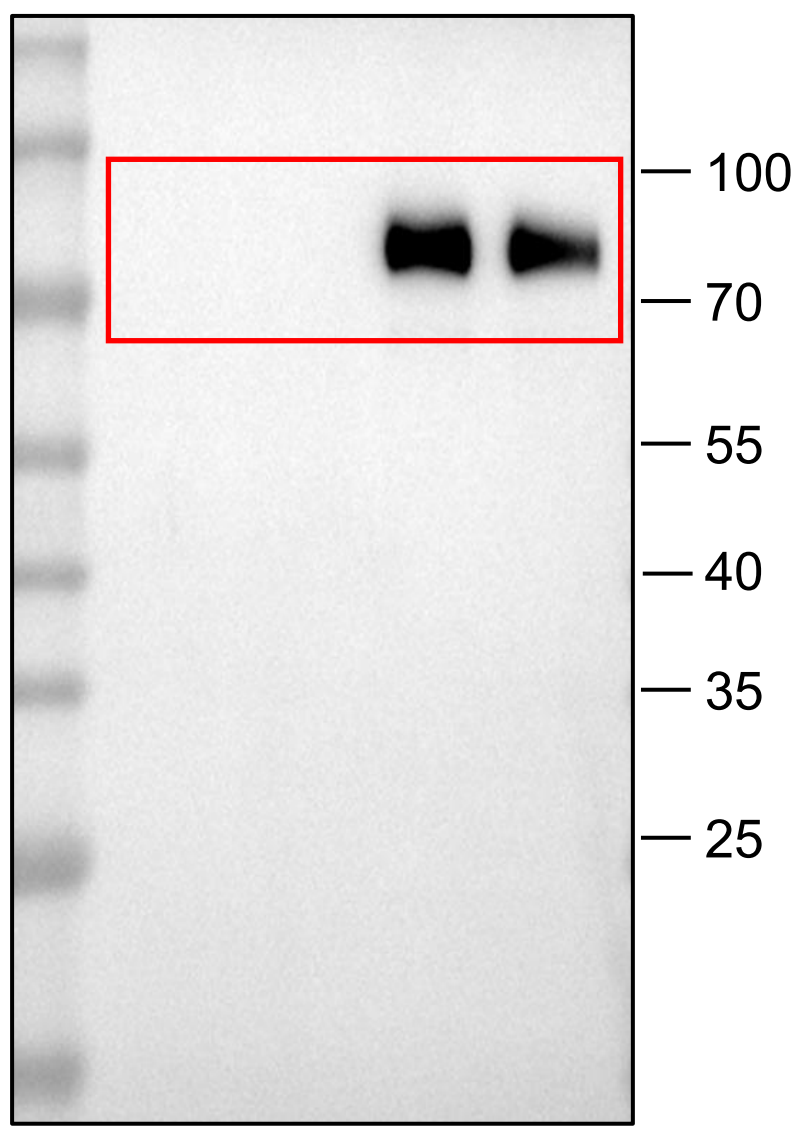

Input

IB: Flag

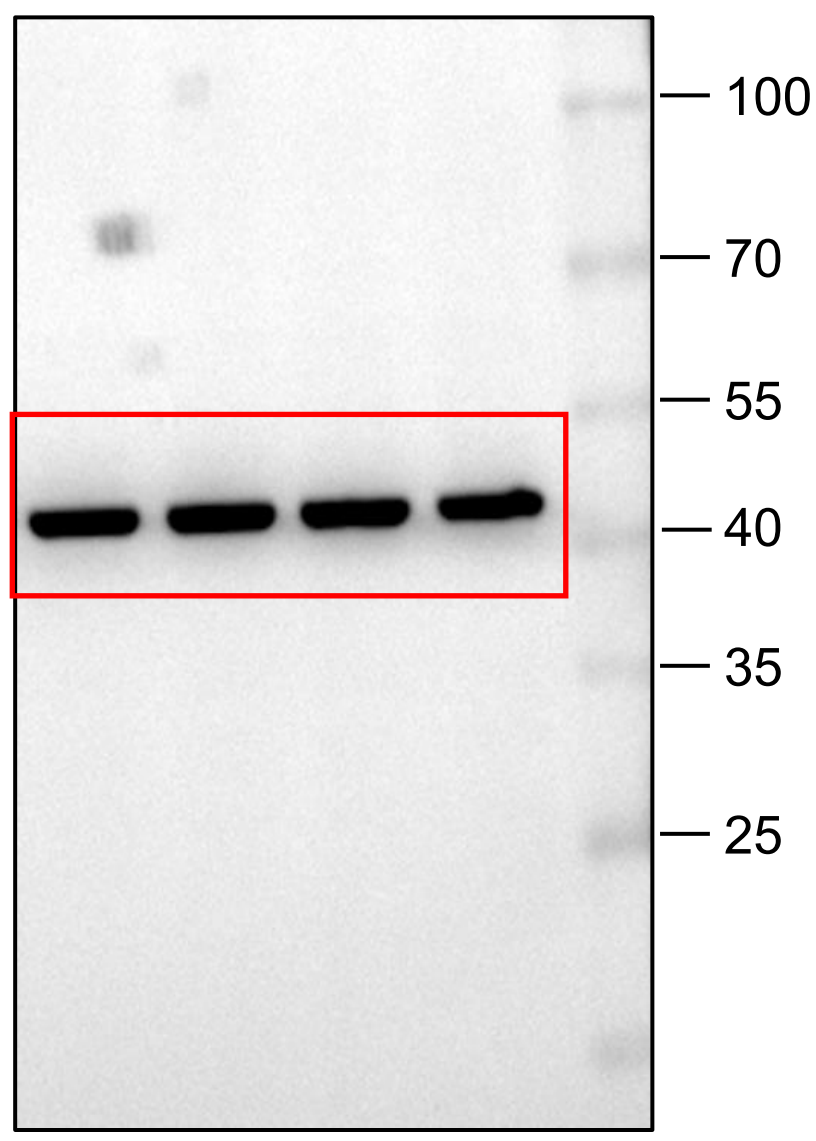

Input

IB: Actin

Figure 1G

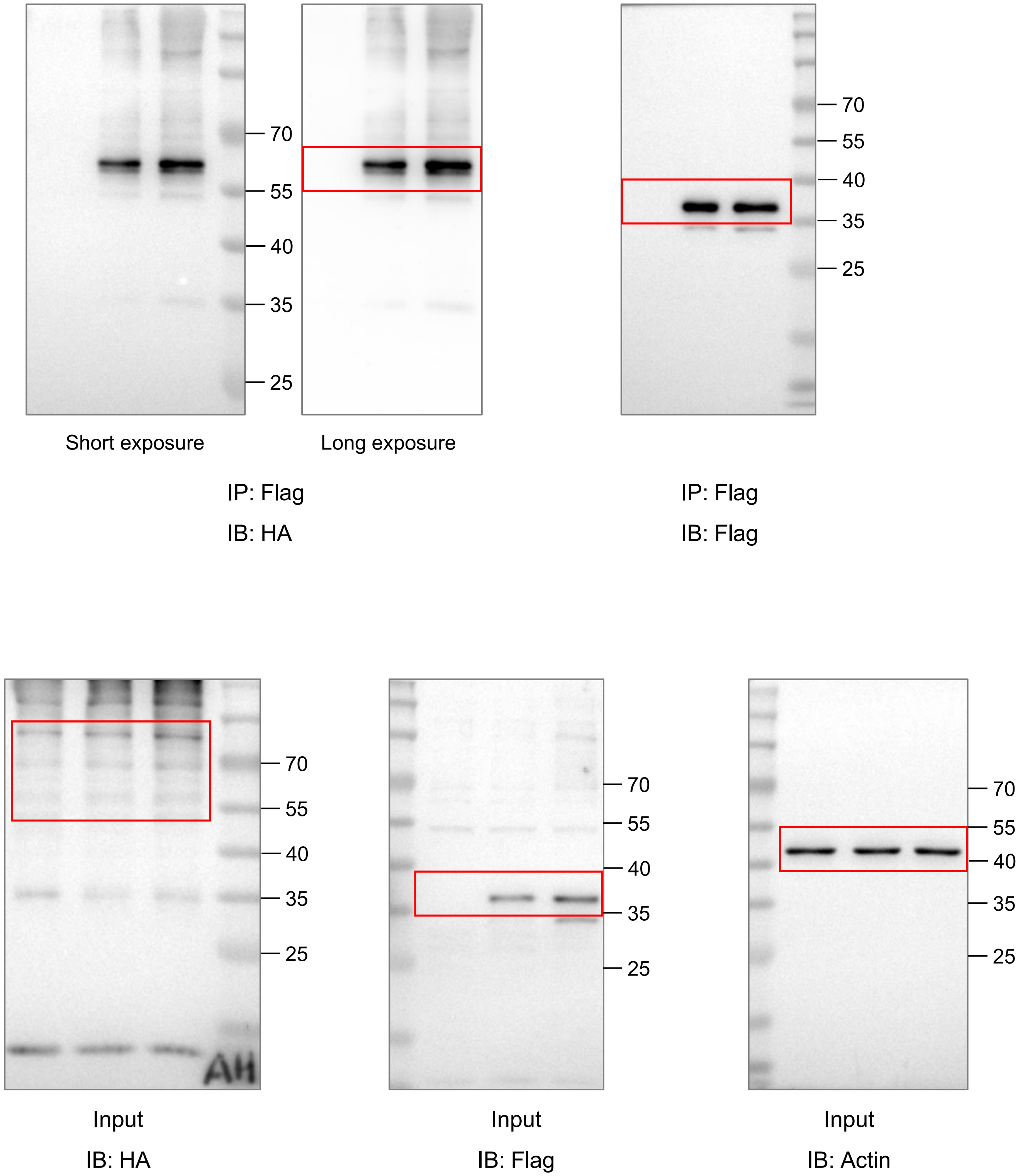

70
55
40
35
25
Short exposure
Long exposure
IP: Flag
IB: HA
70
55
40
35
25
IP: Flag
IB: Flag
70
55
40
35
25
Input
IB: HA
70
55
40
35
25
Input
IB: Flag
70
55
40
35
25
Input
IB: Actin

Figure 1H

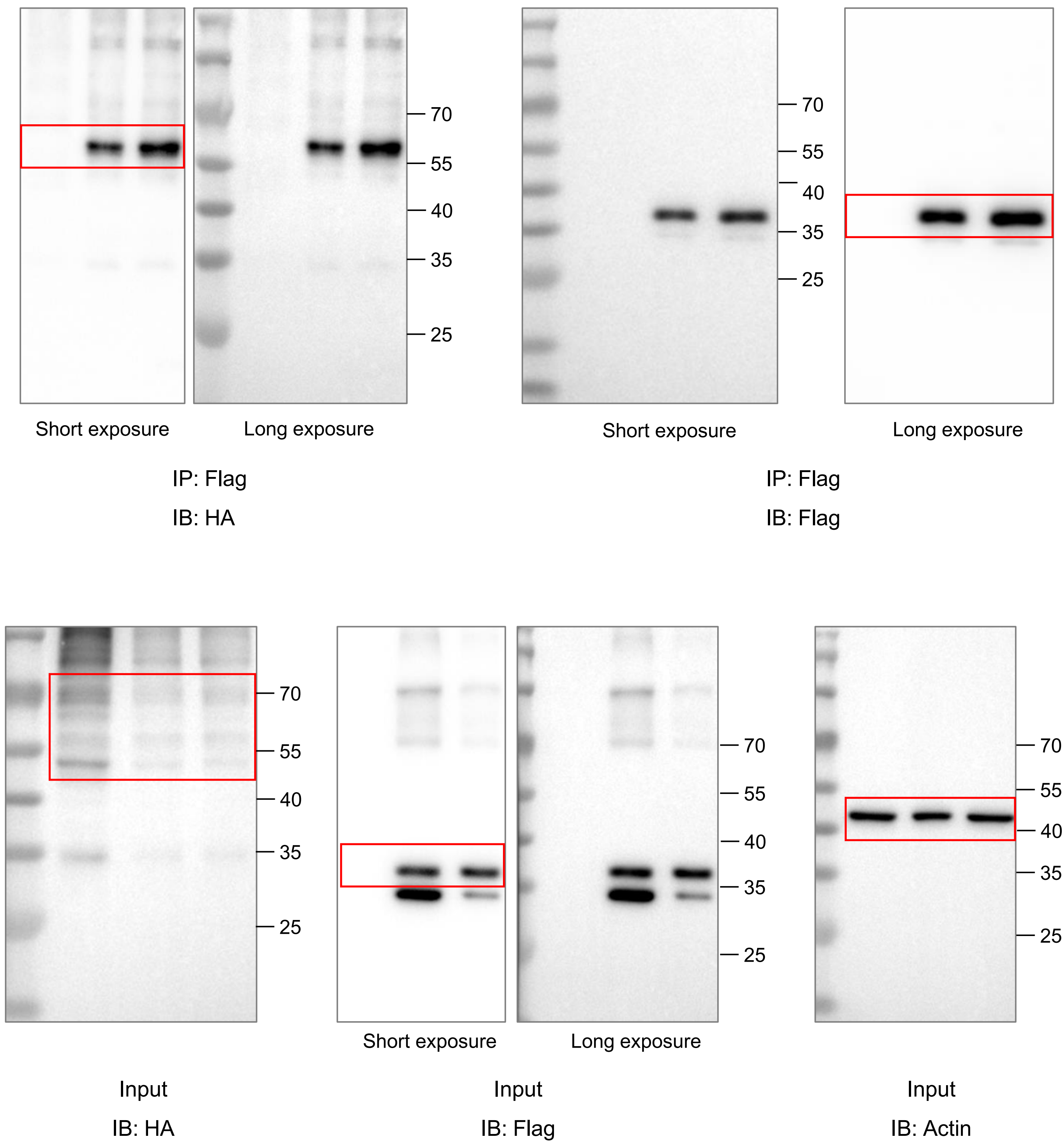
70
55
40
35
25
Short exposure
Long exposure
IP: Flag
IB: HA
70
55
40
35
25
Short exposure
Long exposure
IP: Flag
IB: Flag
70
55
40
35
25
Input
IB: HA
70
55
40
35
25
Short exposure
Long exposure
Input
IB: Flag
70
55
40
35
25
Input
IB: Actin

Figure 1I

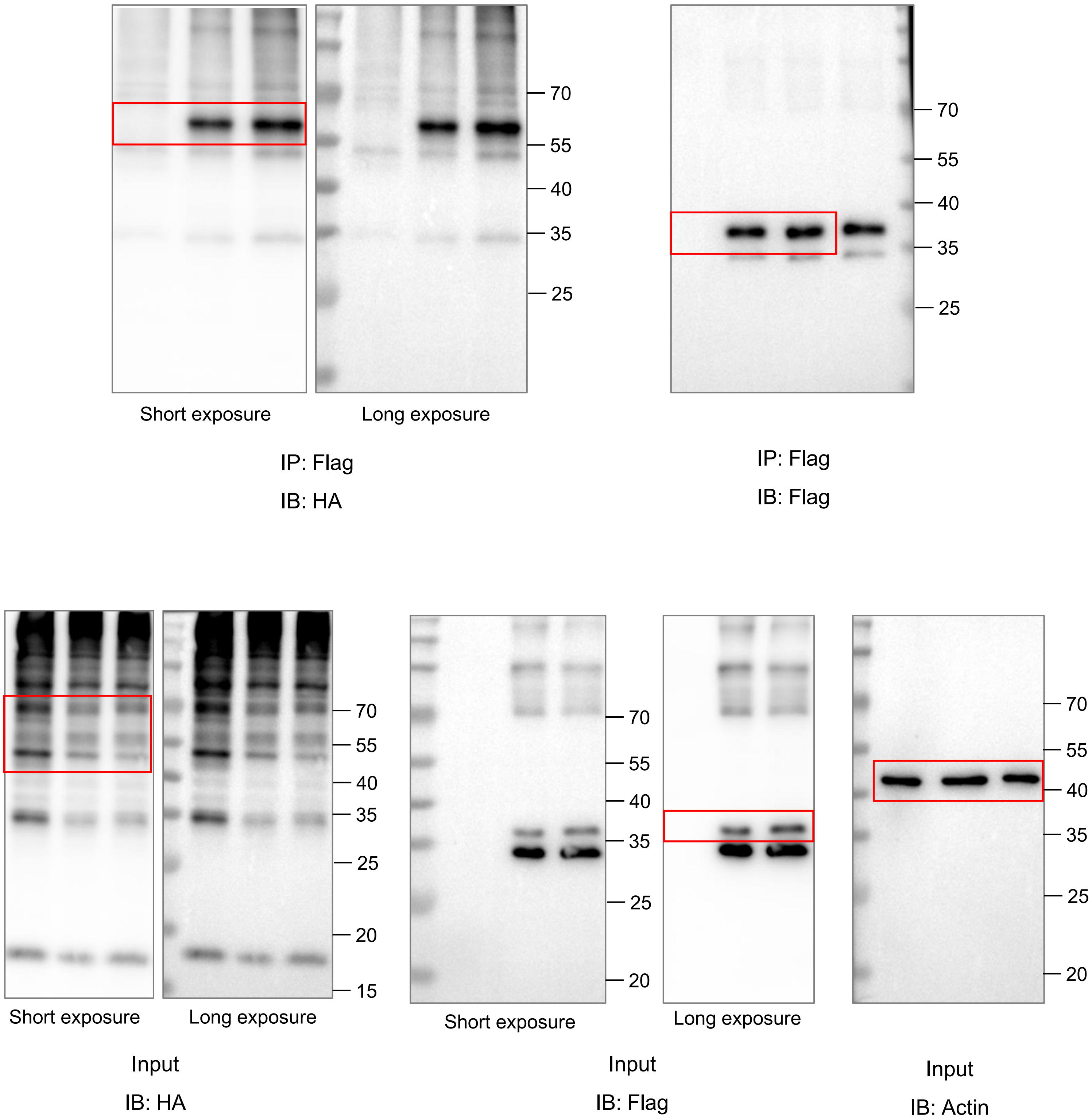

70
55
40
35
25
Short exposure
Long exposure
IP: Flag
IB: HA
IP: Flag
IB: Flag
70
55
40
35
25
20
15
Short exposure
Long exposure
Input
IB: HA
Short exposure
Long exposure
Input
IB: Flag
Input
IB: Actin

Figure 1J

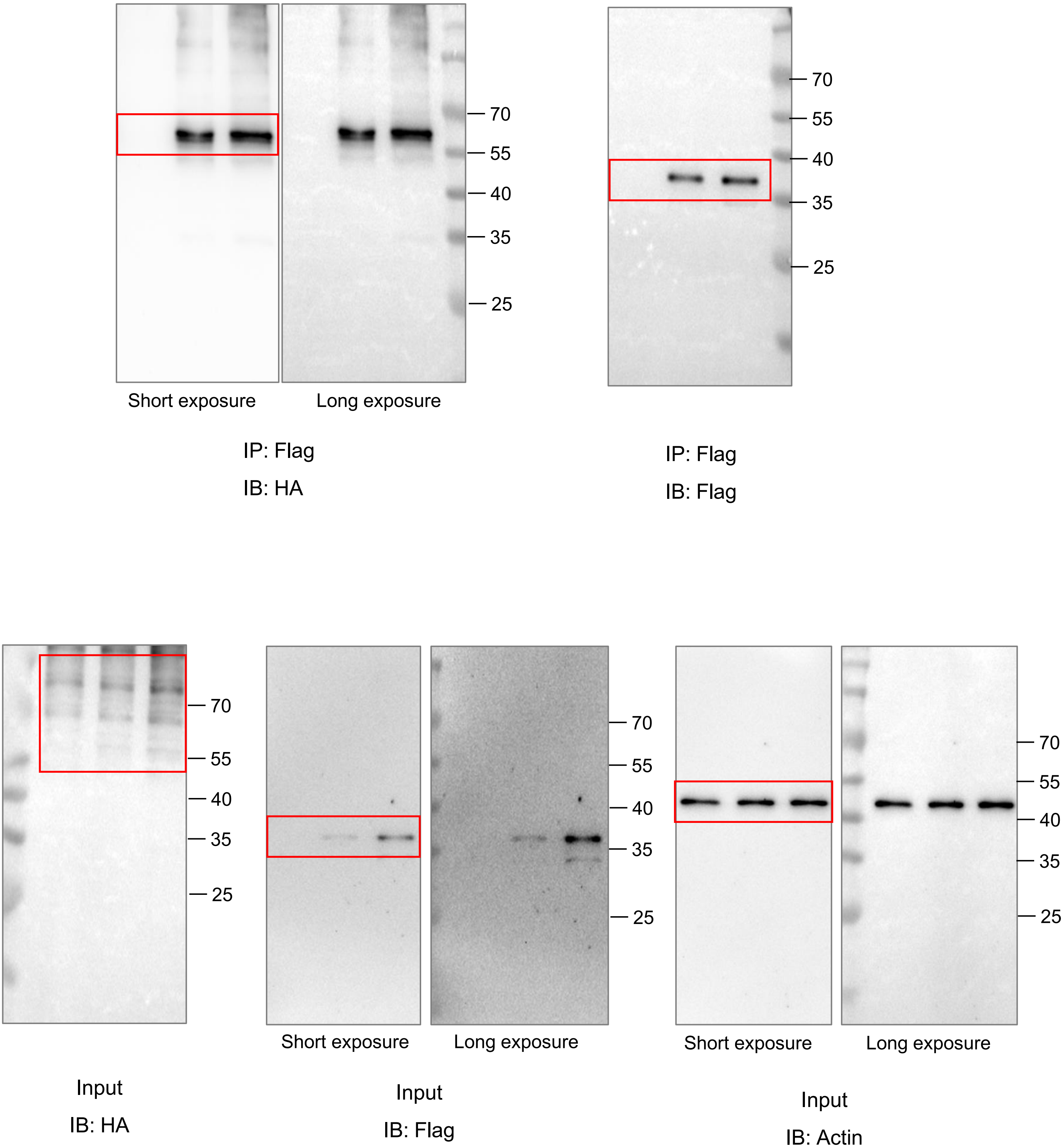

70
55
40
35
25
Short exposure
Long exposure
IP: Flag
IB: HA
70
55
40
35
25
IP: Flag
IB: Flag
70
55
40
35
25
Input
IB: HA
70
55
40
35
25
Short exposure
Long exposure
Input
IB: Flag
70
55
40
35
25
Short exposure
Long exposure
Input
IB: Actin

Figure 2F

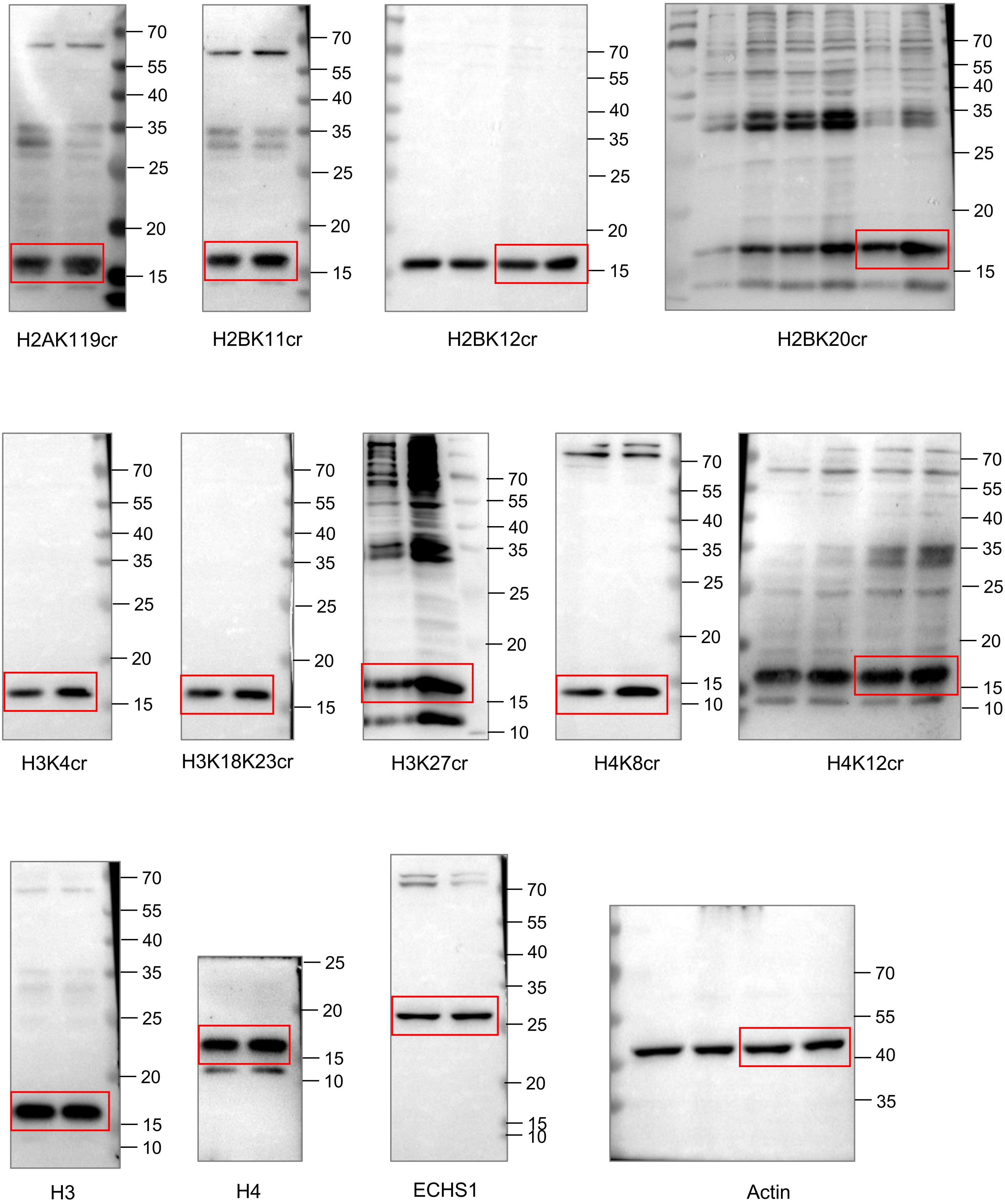
H2AK119cr
70 55 40 35 25 20 15
H2BK11cr
70 55 40 35 25 20 15
H2BK12cr
70 55 40 35 25 20 15
H2BK20cr
70 55 40 35 25 20 15
H3K4cr
70 55 40 35 25 20 15
H3K18K23cr
70 55 40 35 25 20 15
H3K27cr
70 55 40 35 25 20 15 10
H4K8cr
70 55 40 35 25 20 15 10
H4K12cr
70 55 40 35 25 20 15 10
H3
70 55 40 35 25 20 15 10
H4
25 20 15 10
ECHS1
70 55 40 35 25 20 15 10
Actin
70 55 40 35

Figure 2J

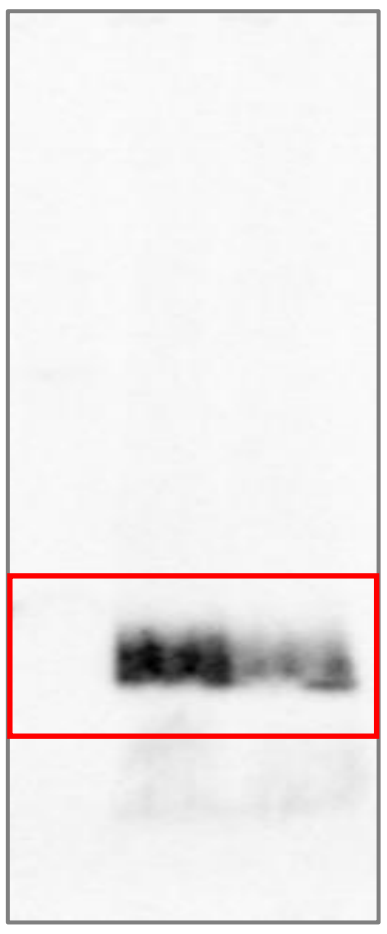

IB: ECHS1

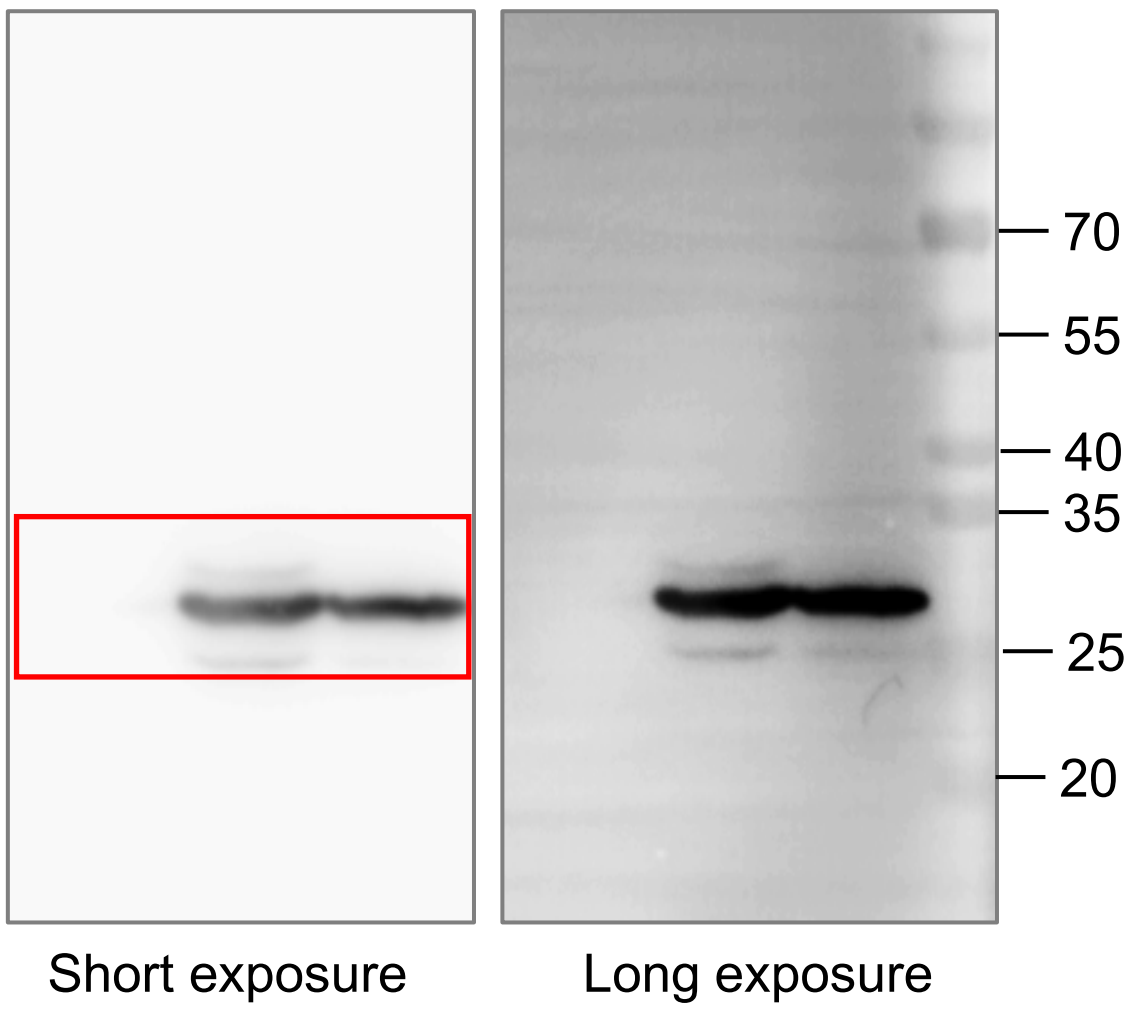

IB: Flag

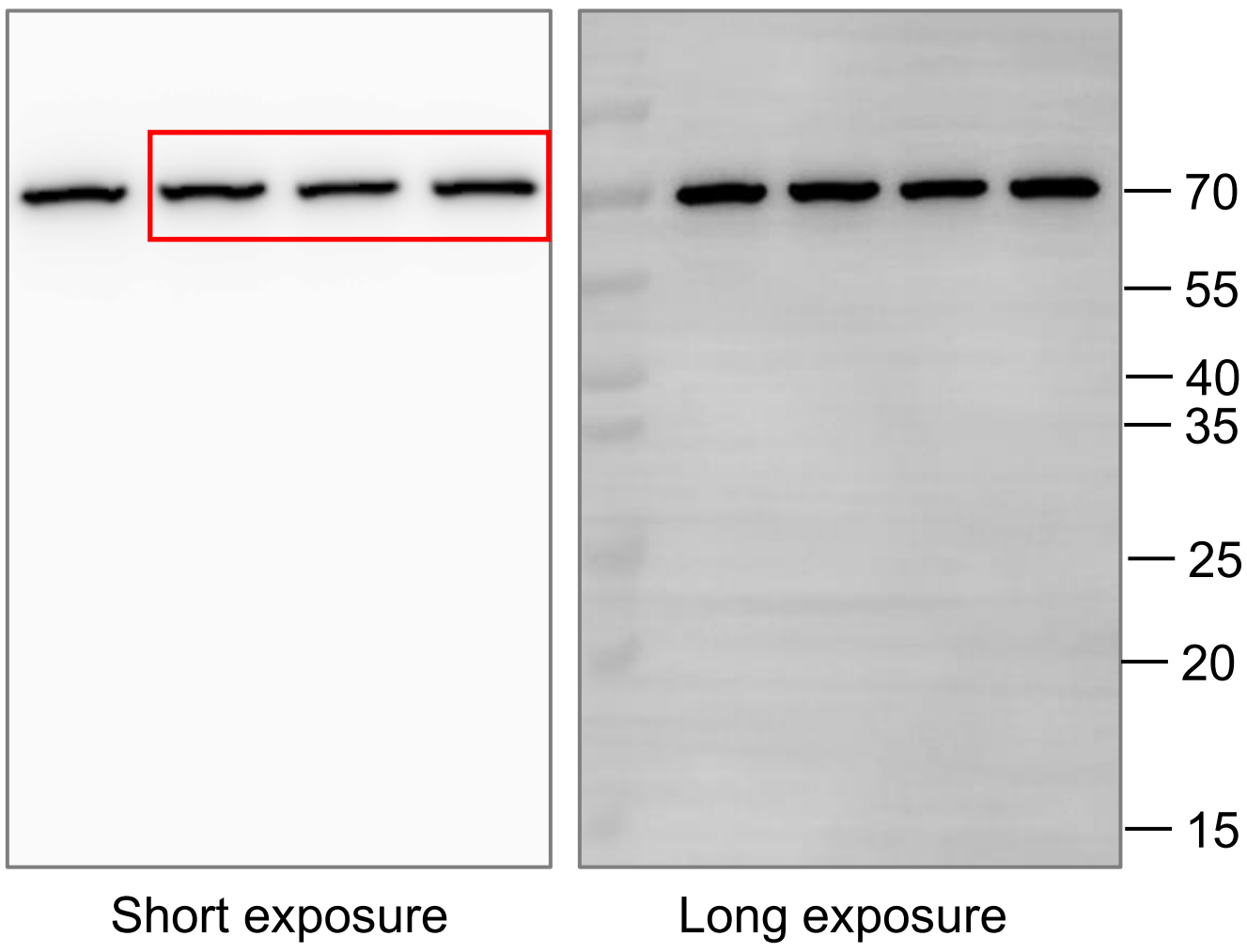

IB: SDHA

Figure 2K

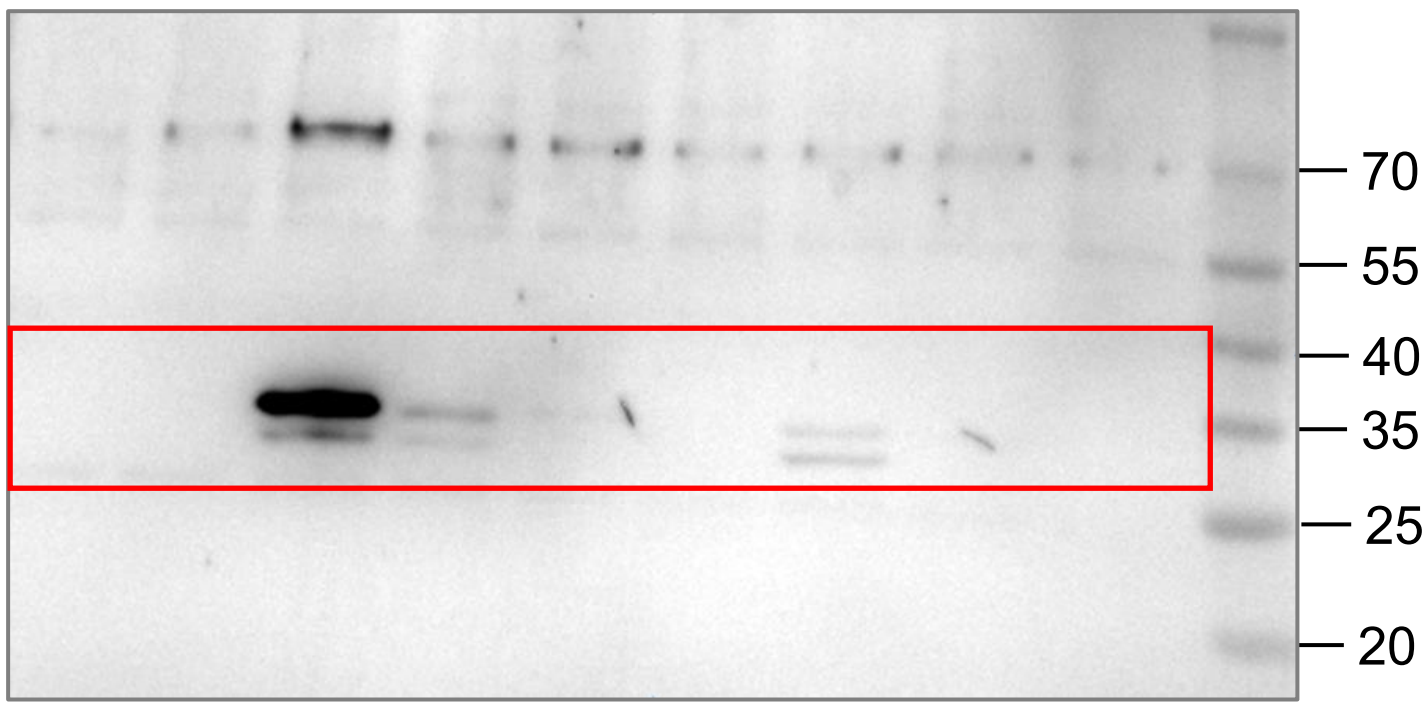

IP: Myc
IB: Flag

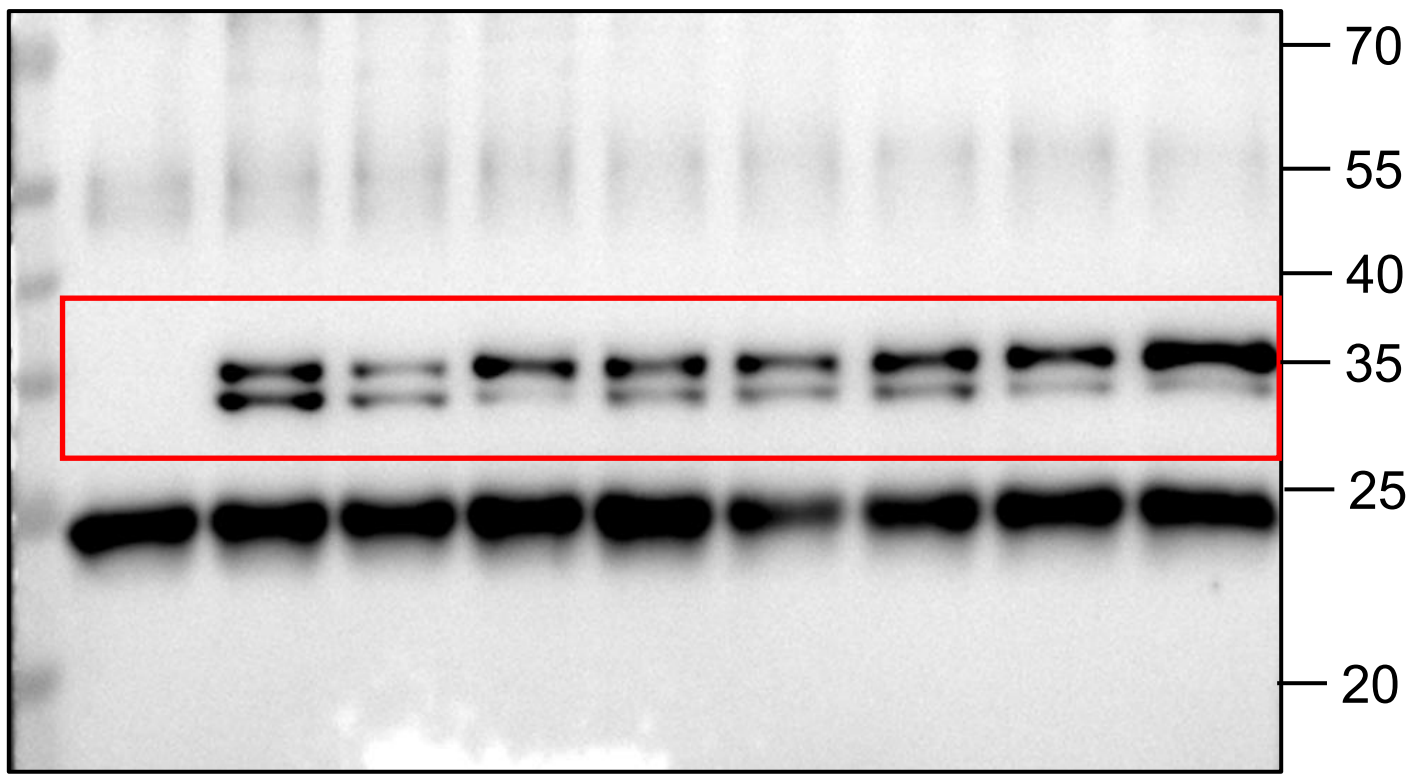

IP: Myc
IB: Myc

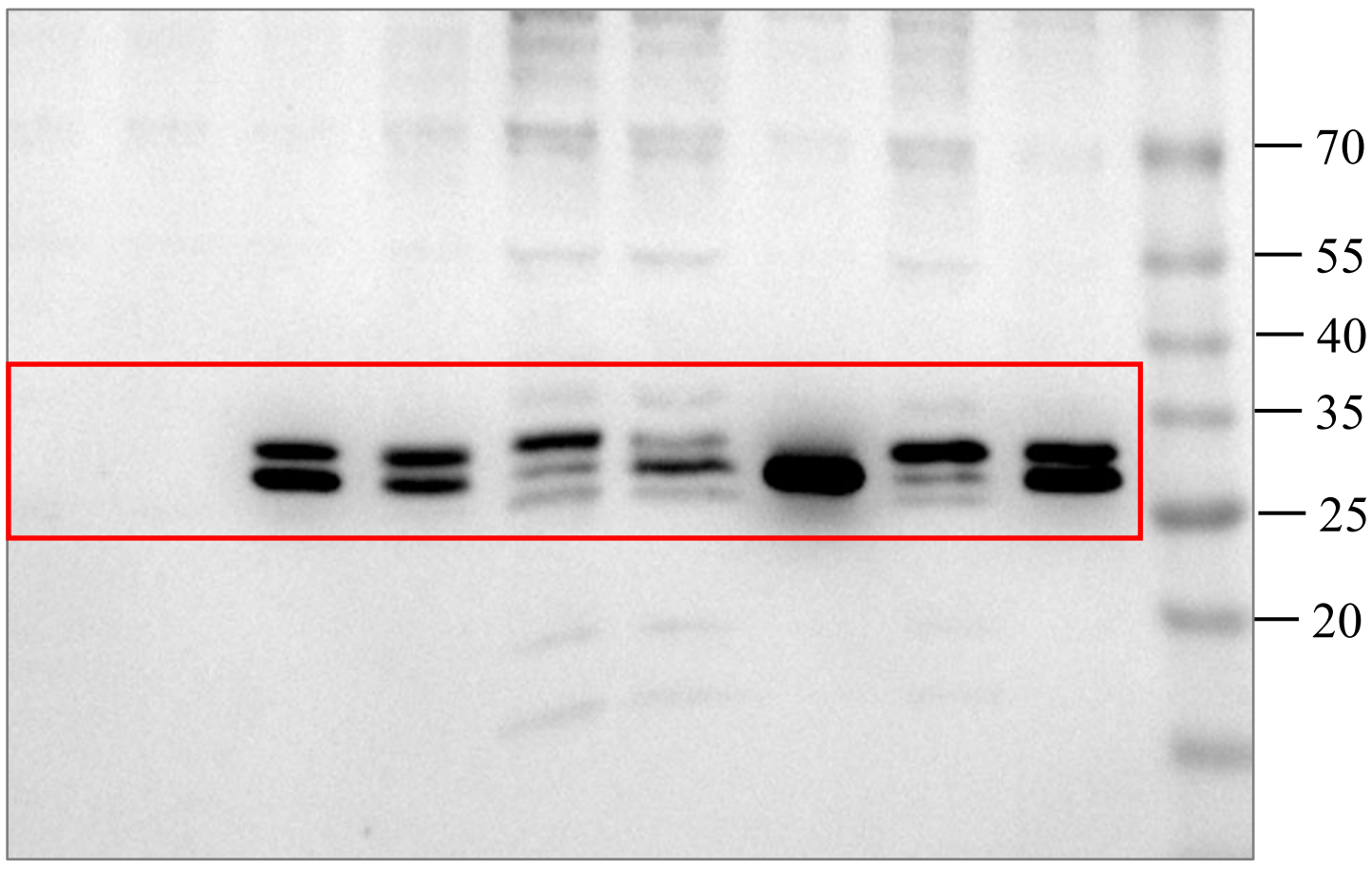

Input
IB: Flag

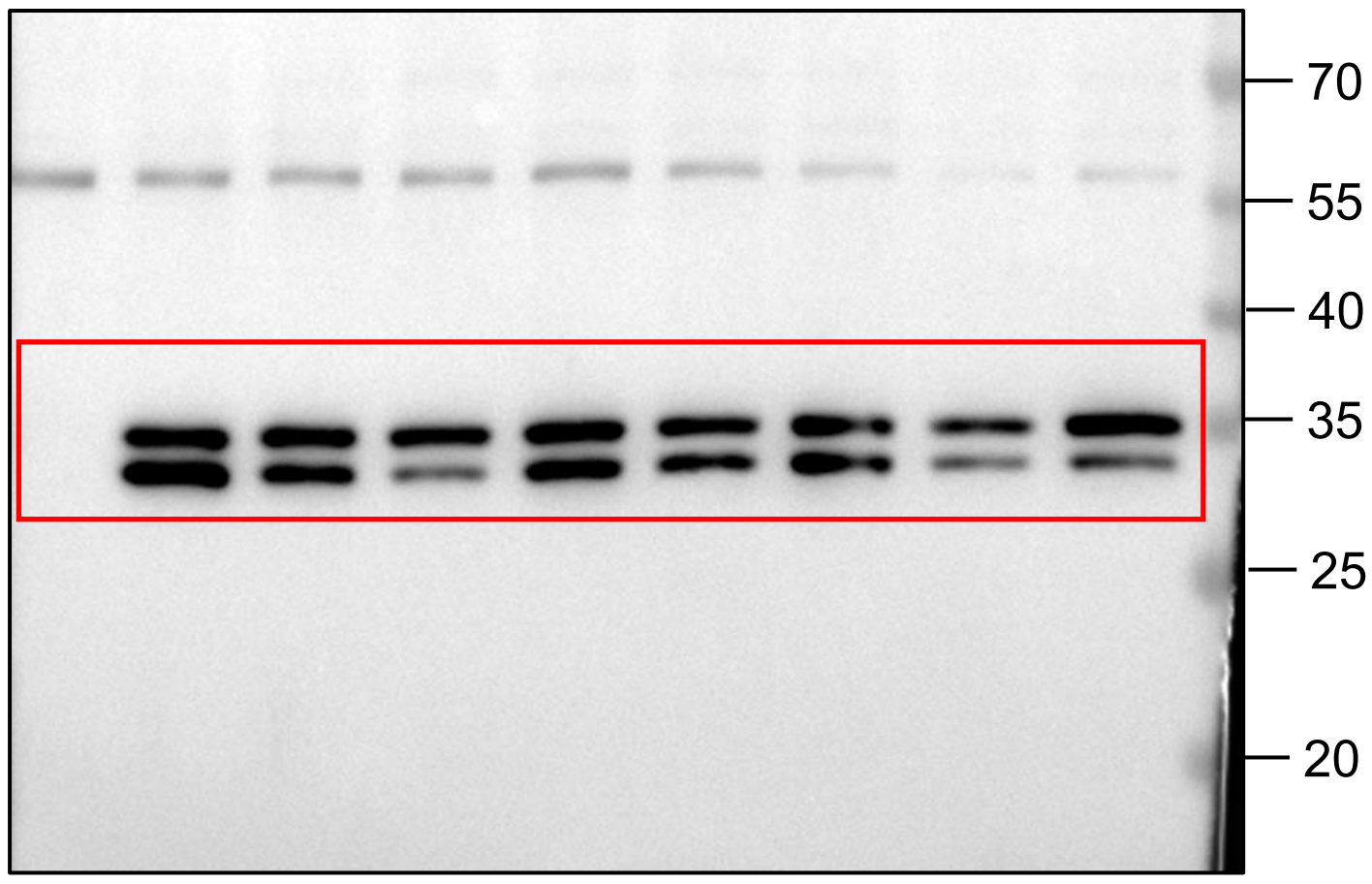

Input
IB: Myc

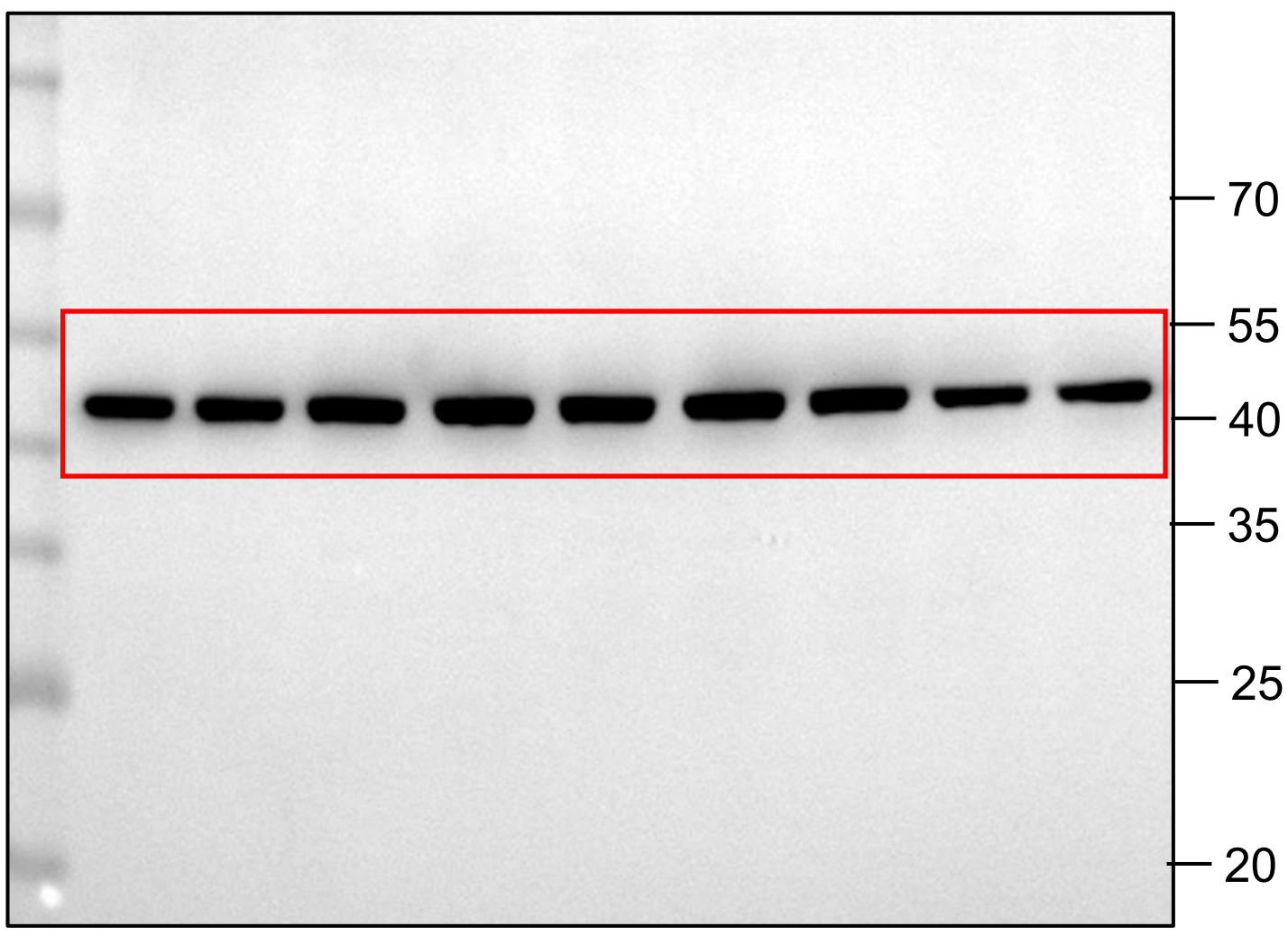

Input
IB: Actin

Figure 3E

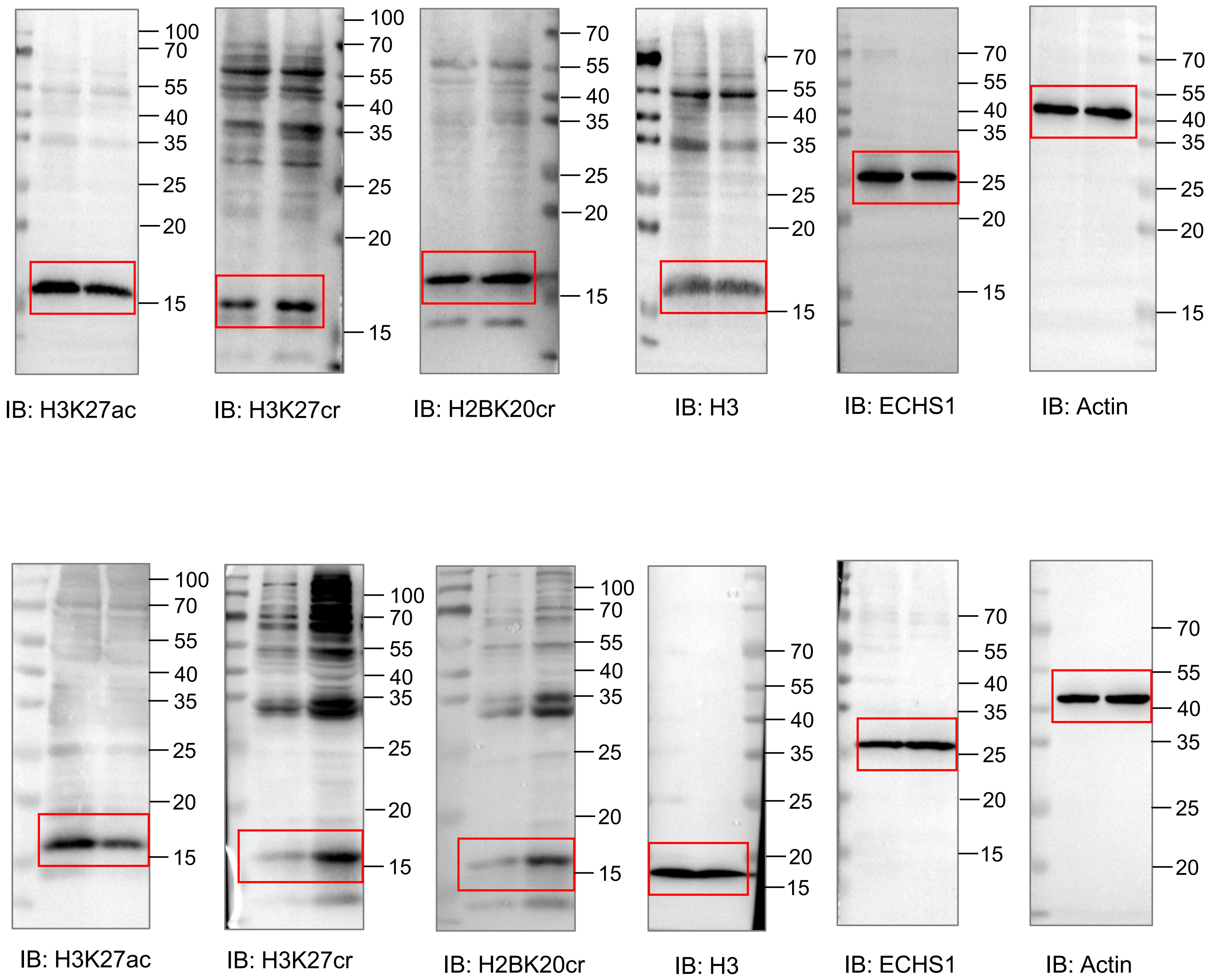

100
70
55
40
35
25
20
15
IB: H3K27ac
IB: H3K27cr
IB: H2BK20cr
IB: H3
IB: ECHS1
IB: Actin
IB: H3K27ac
IB: H3K27cr
IB: H2BK20cr
IB: H3
IB: ECHS1
IB: Actin

Figure 4I

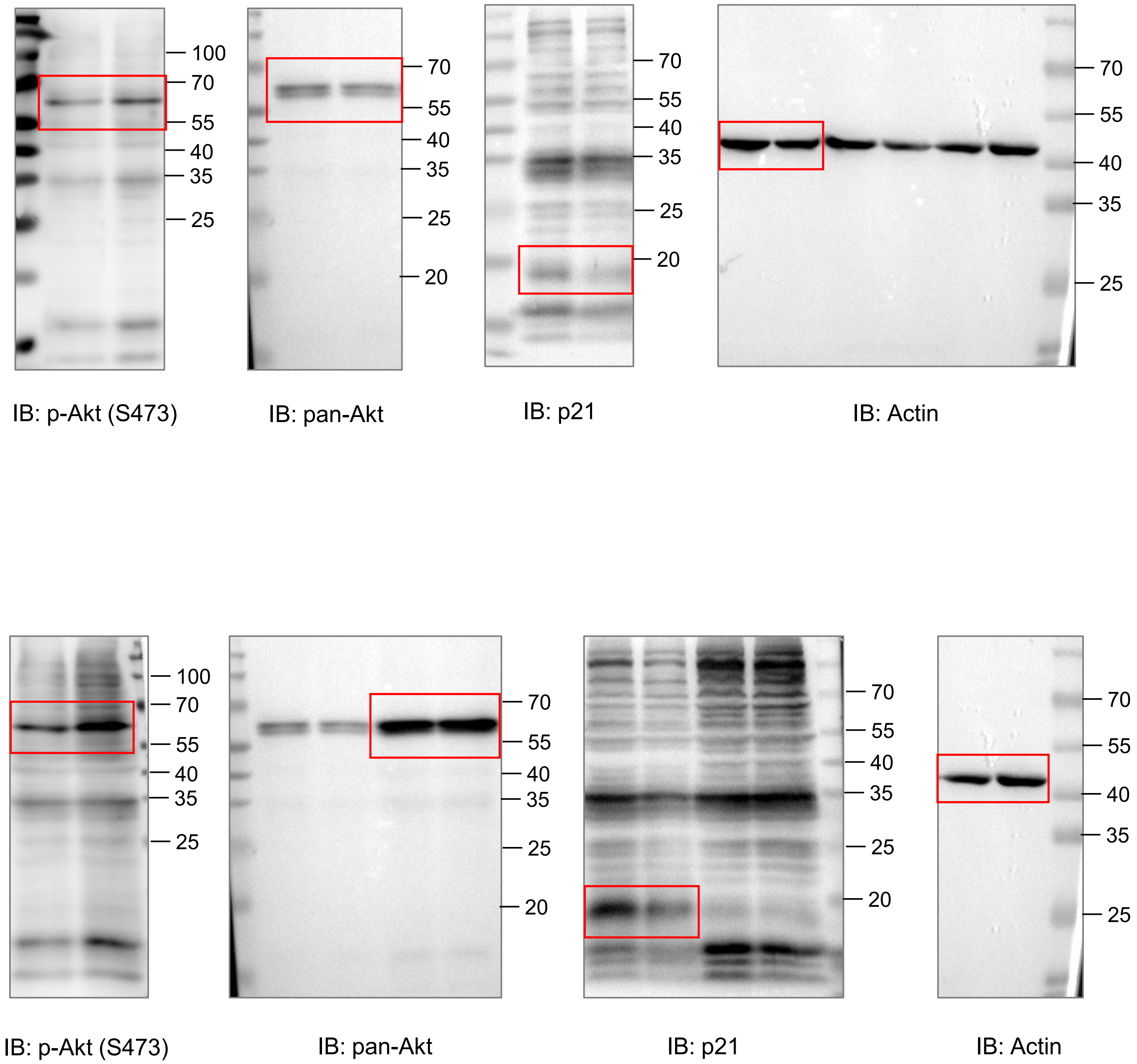
100
70
55
40
35
25
20
IB: p-Akt (S473)
IB: pan-Akt
IB: p21
IB: Actin
IB: p-Akt (S473)
IB: pan-Akt
IB: p21
IB: Actin

Figure 5G

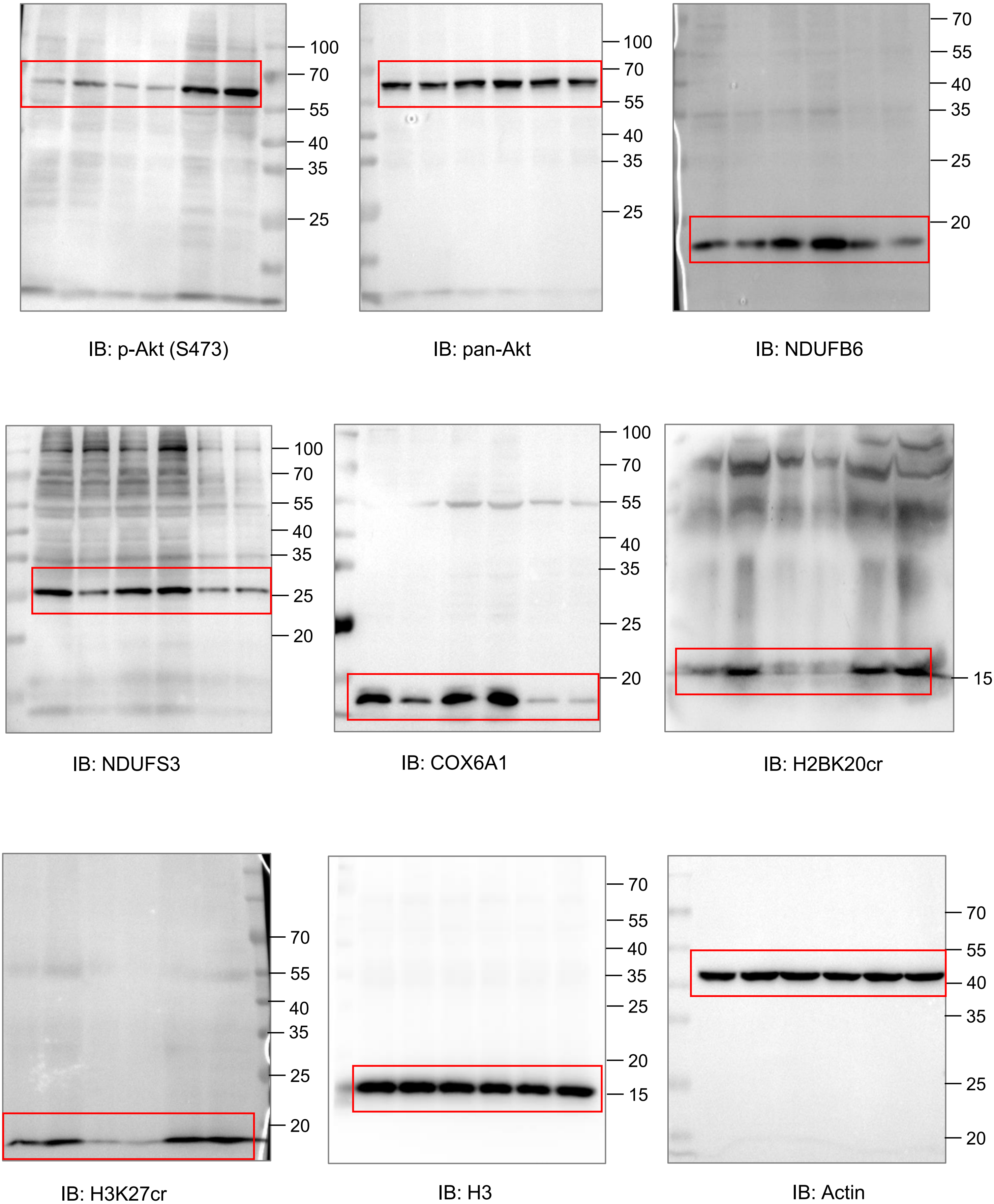
100
70
55
40
35
25
IB: p-Akt (S473)
100
70
55
40
35
25
IB: pan-Akt
70
55
40
35
25
20
IB: NDUFB6
100
70
55
40
35
25
20
IB: NDUFS3
100
70
55
40
35
25
20
IB: COX6A1
15
IB: H2BK20cr
70
55
40
35
25
20
IB: H3K27cr
70
55
40
35
25
20
15
IB: H3
70
55
40
35
25
20
IB: Actin

Supplementary Figure S2A

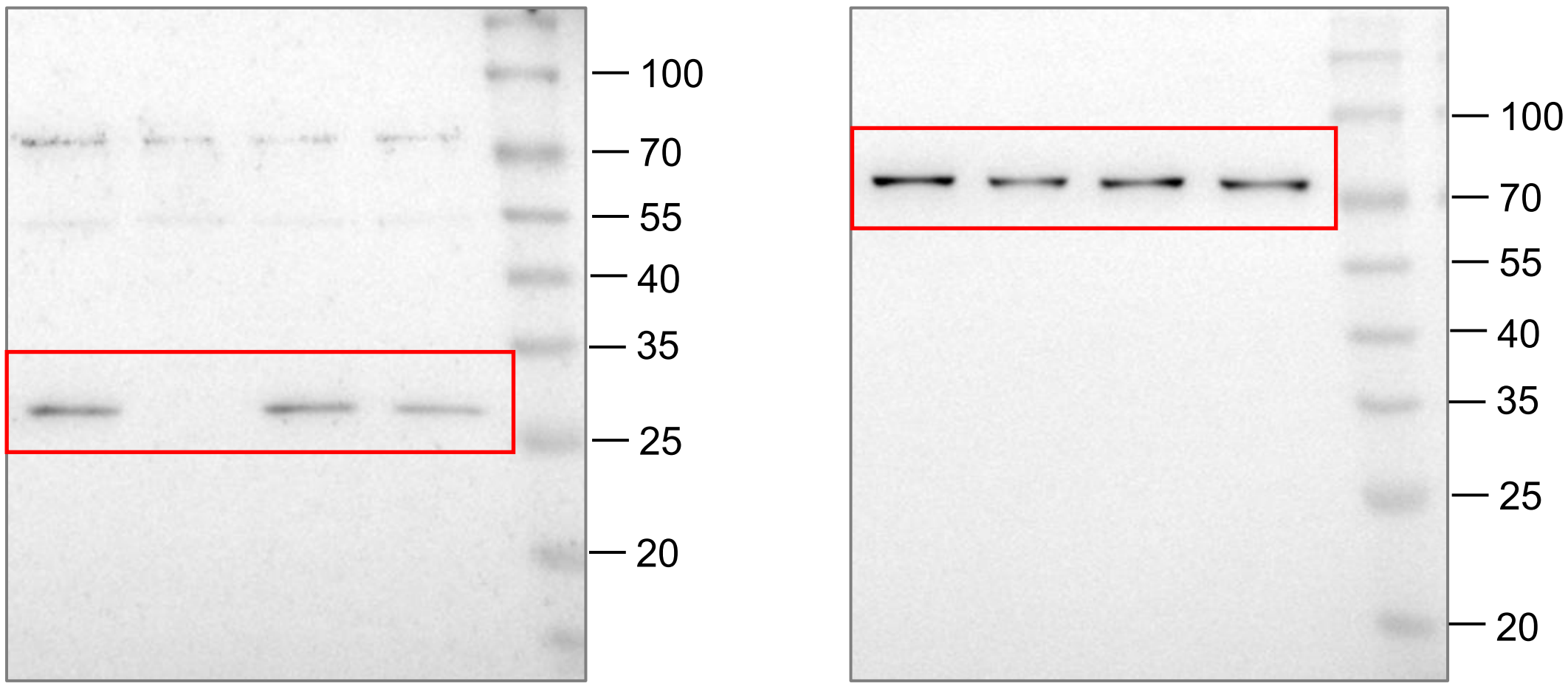

IB: ECHS1

IB: SDHA

Supplementary Figure S2C

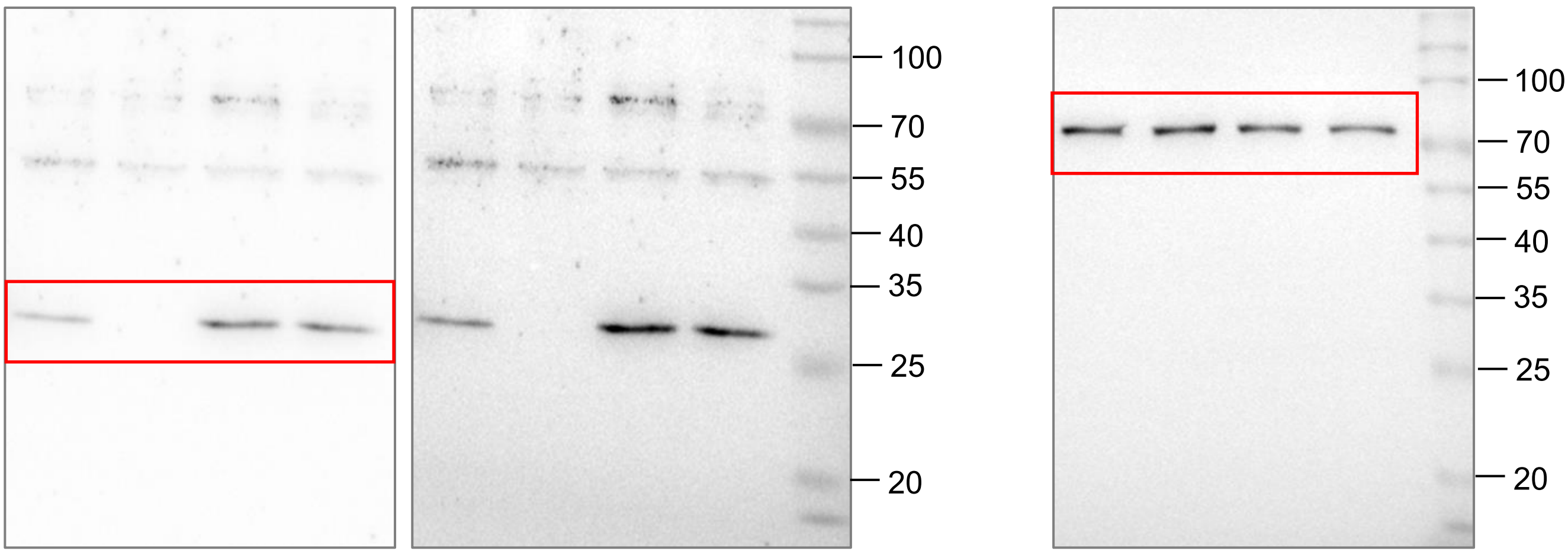

Short exposure

Long exposure

IB: ECHS1

IB: SDHA

Supplementary Figure S2E

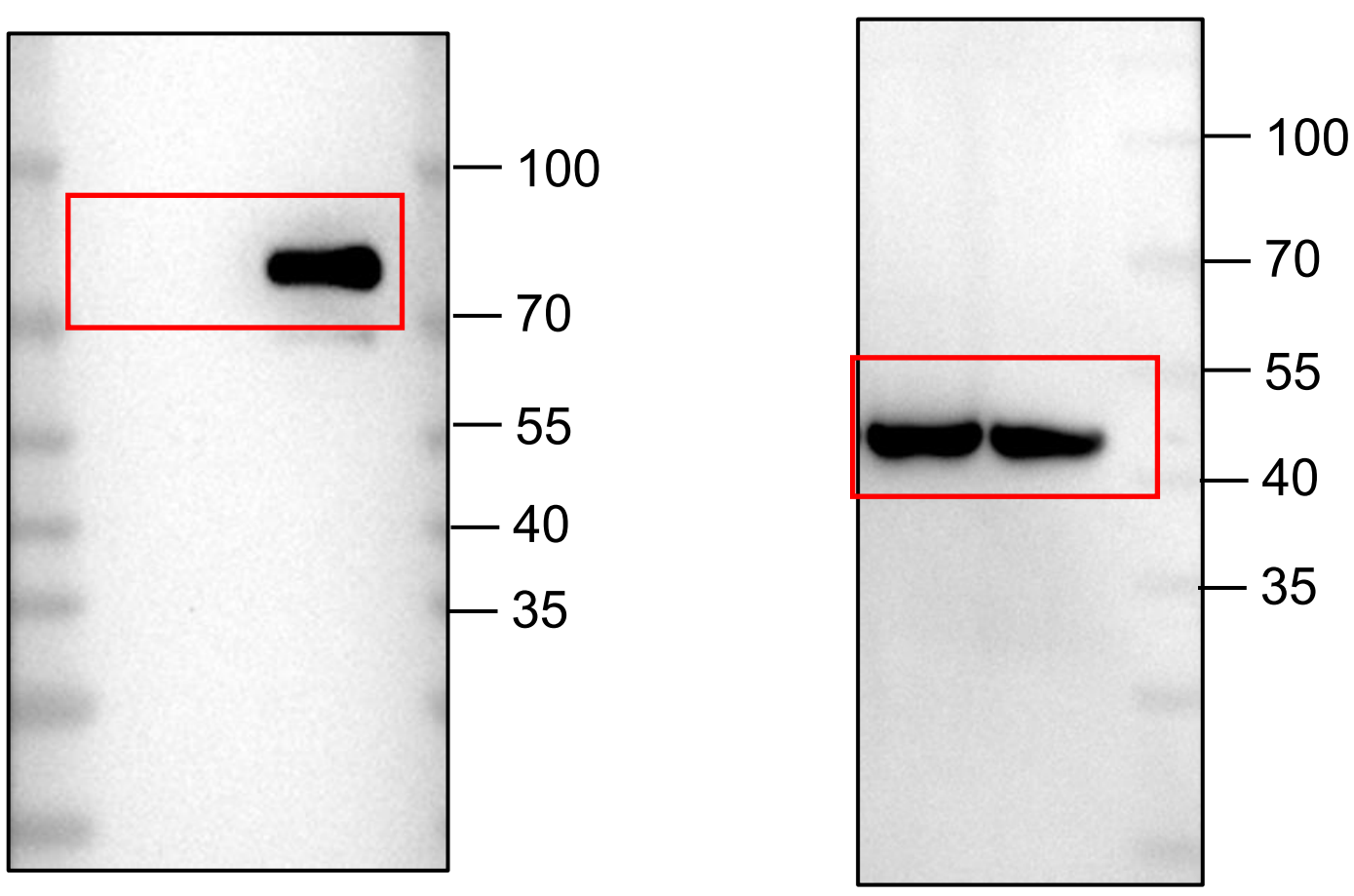

IB: Flag

IB: Actin

Supplementary Figure S2F

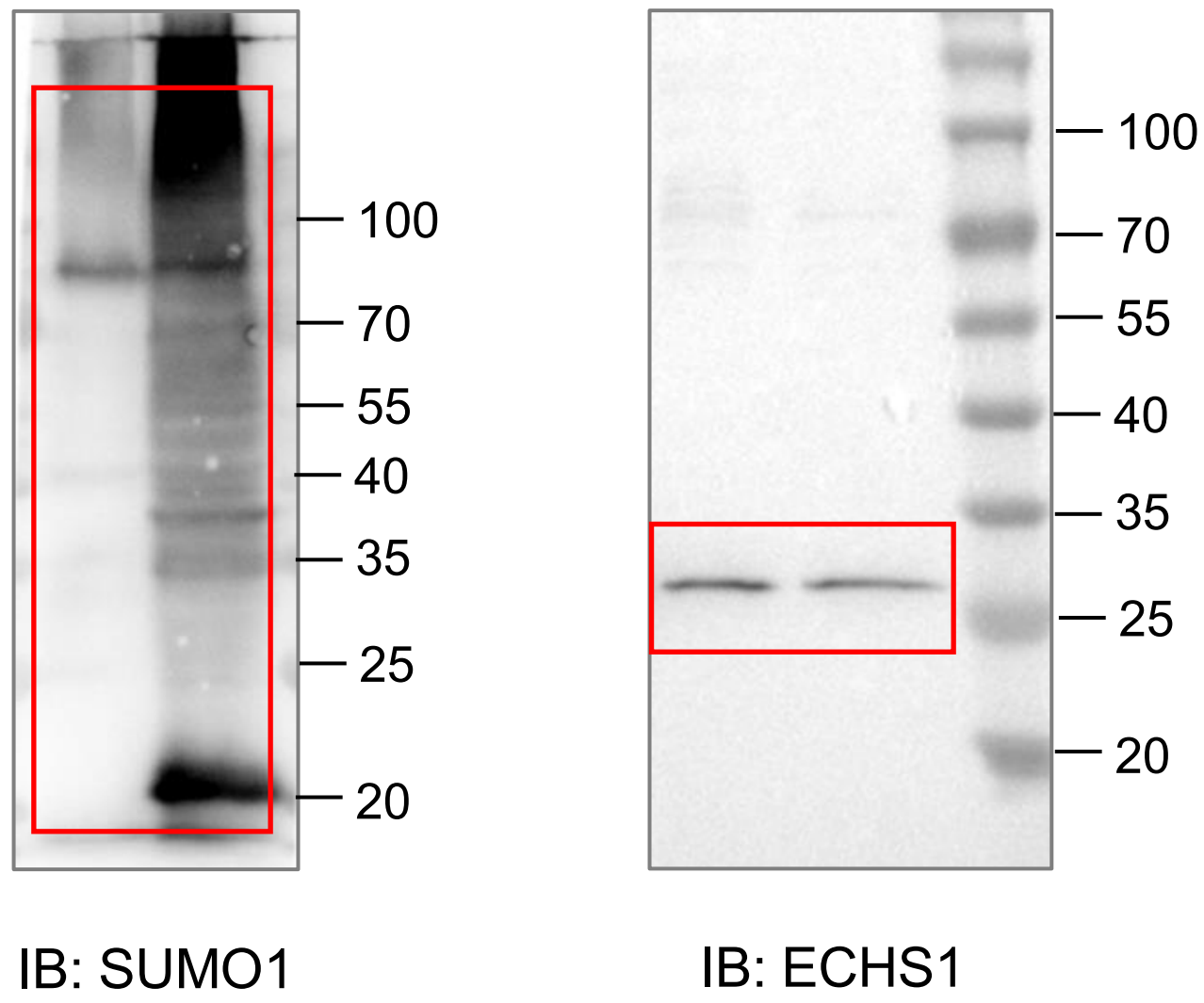

Supplementary Figure S3A

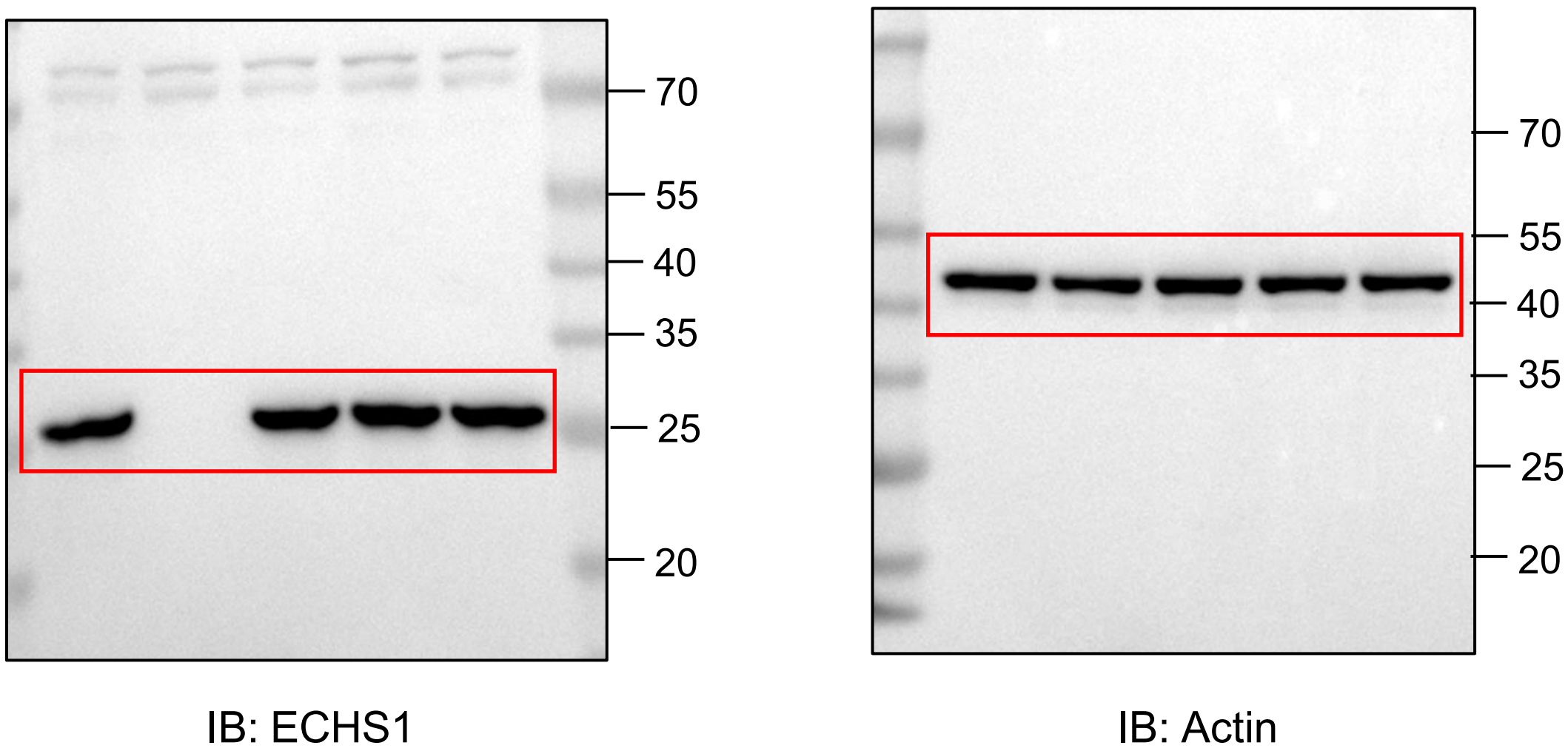

Supplementary Figure S3B

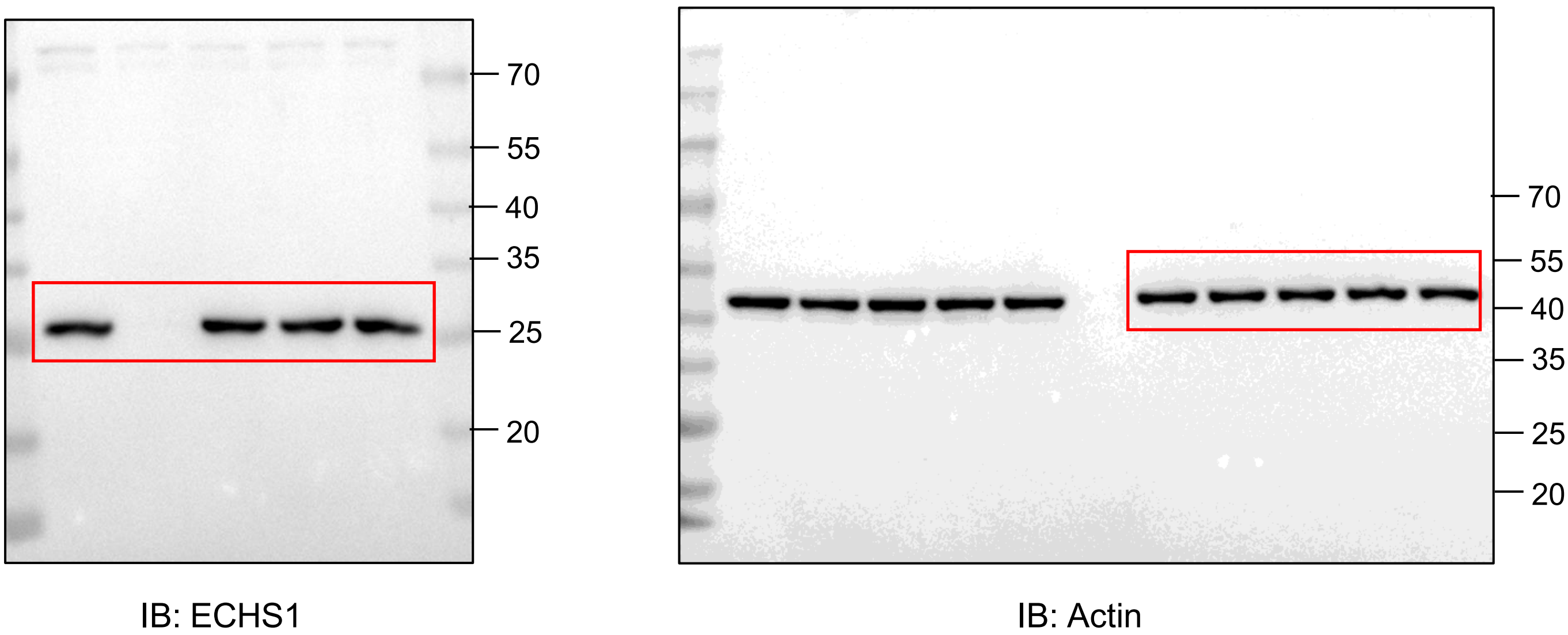

Supplementary Figure S3C

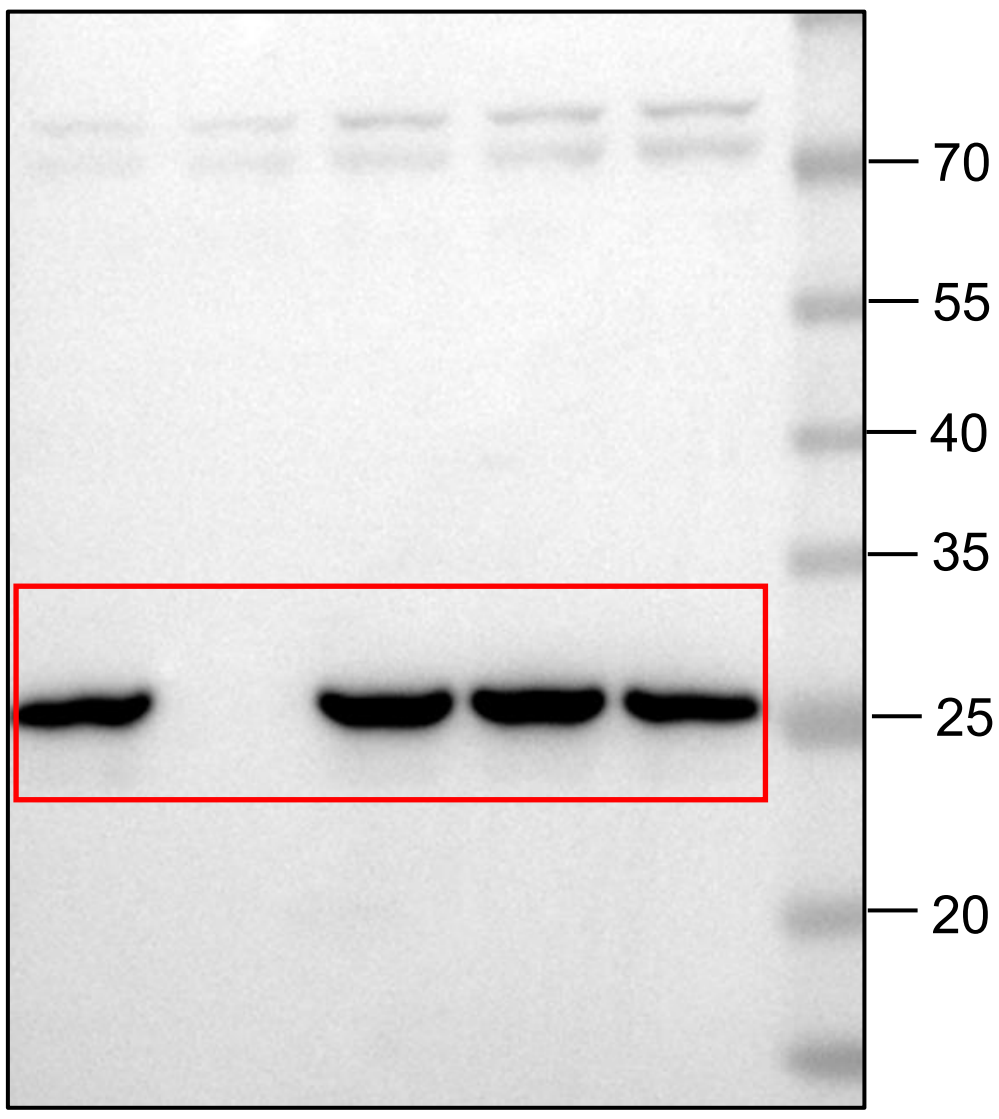

IB: ECHS1

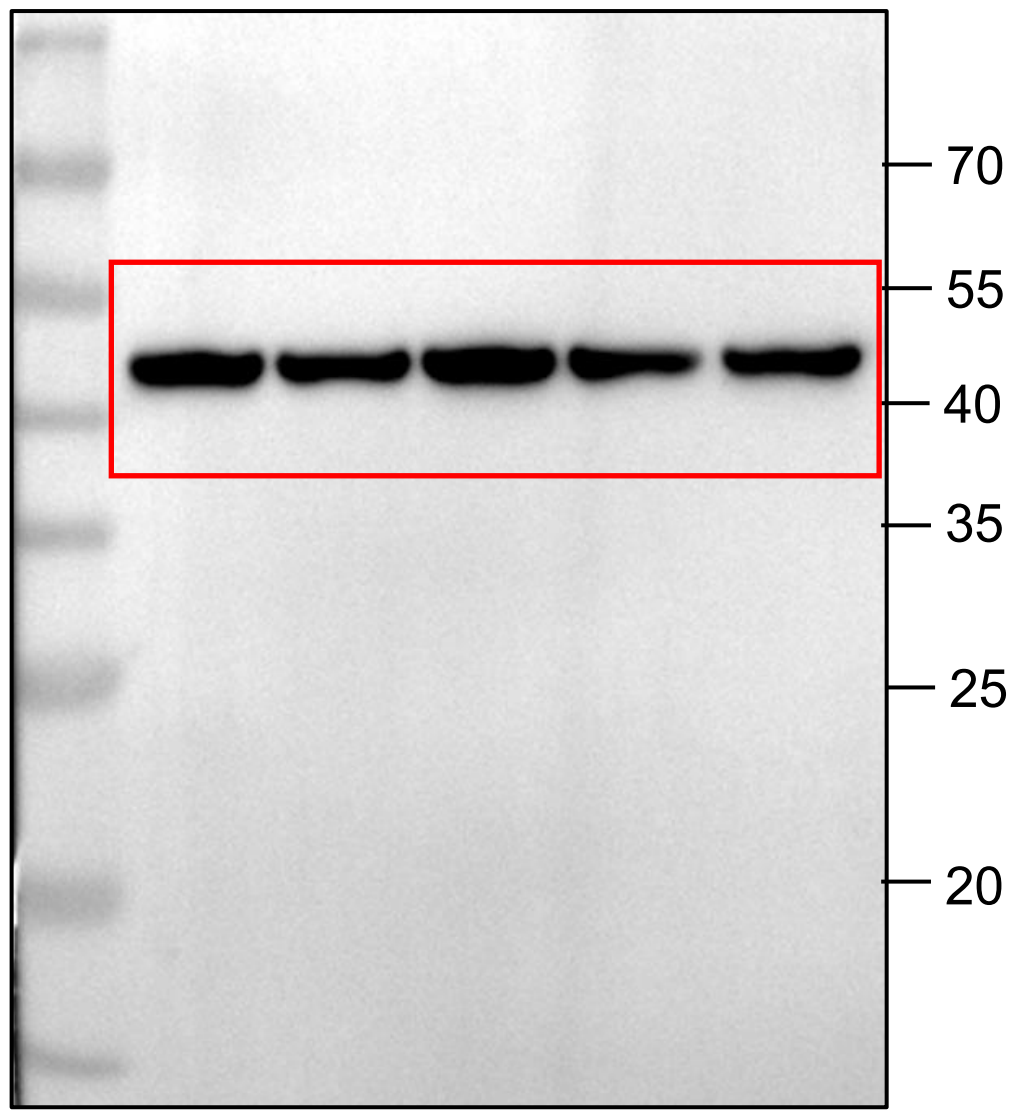

IB: Actin
