# Supplementary material for: SUMOylation activates ECHS1 for adaptive catabolism in lung cancer
Source: Cell Death Dis. 2026 Jul 9;17(1):854. doi: 10.1038/s41419-026-09078-1 (PMC13631240; doi:10.1038/s41419-026-09078-1)
Supplement: Supplementary file 9 [file 41419_2026_9078_MOESM1_ESM.docx]

**Supplementary Figure S1-S5:**

**Supplementary Figure S1. ECHS1 is modified by SUMO1** (Referring to Figure 1)**.** Amino acid sequence of ECHS1. KxΨ motif containing lysine 128 was denoted.

**Supplementary Figure S2. SUMOylation enhances the activity of ECHS1** (Referring to Figure 2)**.** A) Western blotting analysis of indicated cells lysed with NEM. B) Activity analysis of ECHS1 using indicated mitochondria lysed with NEM. C) Western blotting analysis of indicated cells lysed without NEM. D) Activity analysis of ECHS1 using indicated mitochondria lysed without NEM. E) Western blotting confirming overexpression of PIAS4 in HEK293T cells. F) Western blotting confirming overexpression of SUMO1 in HEK293T cells. G) Sketch map of SIMs in ECHS1 with red, blue, and black colors indicating different prediction scores. H-I) Localization of ECHS1 in mitochondria. “+” indicates co-transfection of cells with SUMO1 and PIAS4.

**Supplementary Figure S3. K128R mutation of ECHS1 promotes growth of lung cancer cells** (Referring to Figure 3)**.** A-C) Western blotting analysis of A549, PC-9, and HEK293T stable cell lines with indicated antibodies. D-E) Proliferation of HEK293T stable cell lines cultured in high glucose or low glucose medium. Data on day 4 were analyzed. Error bars represent the SEM values (*n* = 3). Statistical significance (one‐way ANOVA with Dunnett’s multiple comparisons test): ***p* < 0.01, **p* < 0.05.

**Supplementary Figure S4. Mutation of the SUMOylation site of ECHS1 activates PI3K-Akt signaling and suppresses OXPHOS** (Referring to Figure 4)**.** A) PCA of transcripts of ECHS1^WT^ and ECHS1^KR^ cells. B) The volcano plot analysis of differentially expressed genes in ECHS1^KR^ cells compared to ECHS1^WT^ cells. C-D) GSEA reveals significant enrichment of fatty acid degradation and TCA cycle in ECHS1^WT^ cells. E) Q-PCR validation of respiratory chain subunits. Error bars represent the SEM values (*n* = 3). Statistical significance (unpaired *t*-test): *****p* < 0.0001, ****p* < 0.001, **p* < 0.05.

**Supplementary Figure S5. PI3K-Akt signaling and OXPHOS are epigenetically regulated by histone crotonylation** (Referring to Figure 5)**.** A-B) Distribution of H2BK20cr and H3K27cr in genome. C) Overlapping of enriched biological processes of H2BK20cr and H3K27cr biding genes. D) CUT&Tag analysis of specific binding sites of H2BK20cr and H3K27cr in A549 cells. E) Enrichment of H2BK20cr or H3K27cr in promoters of respiratory chain subunits coding genes.

**Supplementary Figure S6. Uncropped Western blot pictures.** The regions adopted in the main manuscript are marked with red boxes.

**Supplementary Tables S1-S3:**

**Supplementary Table S1. Antibodies and primers.** Sheet 1, antibodies used in this study. Sheet 2, primers used in this study.

**Supplementary Table S2.** **Transcriptome of ECHS1-WT and -KR cells.** Sheet 1, upregulated genes in KR cells vs. WT cells. Sheet 2, downregulated genes in KR cells vs. WT cells. Sheet 3, enriched pathways of upregulated genes. Sheet 4, enriched pathways of downregulated genes.

**Supplementary Table S3.** **Functional enrichment of H2BK20cr and H3K27cr targets.** Sheet 1, enriched biological processes of H2BK20cr and H3K27cr overlapped targets. Sheet 2, enriched biological processes of H2BK20cr specific targets. Sheet 3, enriched biological processes of H3K27cr specific targets.
